# Diversity oriented biosynthesis via accelerated evolution of modular gene clusters

**DOI:** 10.1038/s41467-017-01344-3

**Published:** 2017-10-31

**Authors:** Aleksandra Wlodek, Steve G. Kendrew, Nigel J. Coates, Adam Hold, Joanna Pogwizd, Steven Rudder, Lesley S. Sheehan, Sarah J. Higginbotham, Anna E. Stanley-Smith, Tony Warneck, Mohammad Nur-E-Alam, Markus Radzom, Christine J. Martin, Lois Overvoorde, Markiyan Samborskyy, Silke Alt, Daniel Heine, Guy T. Carter, Edmund I. Graziani, Frank E. Koehn, Leonard McDonald, Alexander Alanine, Rosa María Rodríguez Sarmiento, Suzan Keen Chao, Hasane Ratni, Lucinda Steward, Isobel H. Norville, Mitali Sarkar-Tyson, Steven J. Moss, Peter F. Leadlay, Barrie Wilkinson, Matthew A. Gregory

**Affiliations:** 1 Isomerase Therapeutics Ltd., Chesterford Research Park, Cambridge, CB10 1XL UK; 2 Biotica Technology Ltd., Chesterford Research Park, Cambridge, CB10 1XL UK; 3 Department of Biochemistry, University of Cambridge, Tennis Court Road, Cambridge, CB2 1QW UK; 4 Department of Molecular Microbiology, John Innes Centre, Norwich Research Park, Norwich, NR4 7UH UK; 5 Chemical and Screening Sciences, Wyeth Pharmaceuticals, 401 North Middletown Road, Pearl River, NY 10965 USA; 6 Roche Innovation Center Basel, Pharmaceutical Research and Early Development (PRED), Basel, CH-4070 Switzerland; 7 Defence Science and Technology Laboratory, Porton Down, PO17 6AD UK; 8 Present Address: Engineered Biodesign Limited, Cambridge, CB22 3GN UK; 9 Present Address: Department of Pharmacognosy, College of Pharmacy, King Saud University, Riyadh, 12372 Saudi Arabia; 10 Present Address: BASF SE, Speyerer Str. 2, Limburgerhof, 67117 Germany; 11 Present Address: Medicine Discovery Network-Synthetic Biology, Pfizer Worldwide R&D, 445 Eastern Point Rd., Groton, CT 06340 USA; 12 Present Address: Marshall Centre for Infectious Diseases, School of Biomedical Sciences, University of Western Australia, Monash Avenue, Nedlands, WA 6009 Australia

## Abstract

Erythromycin, avermectin and rapamycin are clinically useful polyketide natural products produced on modular polyketide synthase multienzymes by an assembly-line process in which each module of enzymes in turn specifies attachment of a particular chemical unit. Although polyketide synthase encoding genes have been successfully engineered to produce novel analogues, the process can be relatively slow, inefficient, and frequently low-yielding. We now describe a method for rapidly recombining polyketide synthase gene clusters to replace, add or remove modules that, with high frequency, generates diverse and highly productive assembly lines. The method is exemplified in the rapamycin biosynthetic gene cluster where, in a single experiment, multiple strains were isolated producing new members of a rapamycin-related family of polyketides. The process mimics, but significantly accelerates, a plausible mechanism of natural evolution for modular polyketide synthases. Detailed sequence analysis of the recombinant genes provides unique insight into the design principles for constructing useful synthetic assembly-line multienzymes.

## Abstract (web summary)

Reengineering polyketide synthase encoding genes to produce analogues of natural products can be slow and low-yielding. Here the authors use accelerated evolution to recombine the gene cluster for rapid production of rapamycin-related products.

## Introduction

Reduced polyketide natural products (NPs) produced by modular polyketide synthase (PKS) assembly lines are a rich source of clinically approved products including antibacterial (erythromycin, fidaxomicin), immunosuppressive (rapamycin, FK506) and anthelminthic (avermectin) agents^1^. These structurally complex NPs are biosynthesized through the decarboxylative condensation of (alkyl)malonyl-CoA extender units on modular PKSs in a process similar to that of fatty acid biosynthesis^2^. The genetic basis of reduced polyketide biosynthesis was first described in 1990–1991^3, 4^ and shown to depend upon the expression of highly repetitive genes encoding large multifunctional enzymes containing a separate module of enzymatic activities for every round of polyketide chain extension. Each module comprises the individual catalytic domains required for each chemical step in the process. This direct relationship between the sequence of PKS-encoding genes and the structure of the product molecule immediately suggested the potential for rational genetic modification to yield strains capable of producing analogues of predictable structure, and although then many groups have attempted to bioengineer these systems with varying success^5–7^. Several examples of targeted in vivo analogue production at reasonable titre have been described^8–10^, although a reduction in productivity often accompanies the generation of hybrid or modified PKSs^11–13^. This approach is most suited to producing focussed libraries closely related in structure to the parent molecule, limiting its industrial application to lead optimisation. There are few published examples of its use for generating novel products capable of hitting new targets^14^. Despite the publication of detailed structural information for these enzymes^15, 16^, one of the major barriers to progress in engineering modular PKSs has been the lack of an experimental knowledge base to guide the optimal choice of splice points to generate hybrid PKSs^17, 18^.

During an experiment to rationally bioengineer the rapamycin biosynthetic gene cluster (BGC), by replacing enzymatic domains within the PKS, we made the serendipitous observation that, instead of the expected product, a variety of unexpected analogues (rapalogs) were produced at good to excellent titres by the various progeny isolated from the experiment after selecting for a secondary crossover event (allelic exchange). Following isolation and structure elucidation it became clear that these compounds were related to rapamycin, but consisted of molecules lacking between 2 and 12 carbon atoms from the polyketide chain backbone, or, in one instance, containing 2 carbons extra. Biosynthetic considerations suggested that these would arise from truncated or expanded PKS multienzymes, respectively, and this was confirmed by whole-genome sequencing of the relevant mutant strains. These observations were reproducible for the rapamycin PKS, and could be recapitulated in a second modular PKS-encoding BGC (for the antibiotic tylosin).

We now report the structures of these rapalogs and the gene sequences encoding their biosynthesis. A mechanism for this process is proposed based on stochastic recombination between repeating regions of high-sequence homology that occur in the genes encoding for modular biosynthetic systems. This is driven by insertion, through homologous recombination, of a plasmid encoding a temperature sensitive, high-copy replicon into the modular PKS-encoding genes under non-permissive conditions. On lowering the temperature to that permissive of replication, the integrated replicon causes an otherwise catastrophic event that is resolved by loss of the region comprising the integrated replicon after homologous recombination between flanking PKS-encoding genes.

## Results

### Bioengineering of the rapamycin PKS

**Fig. 1:**
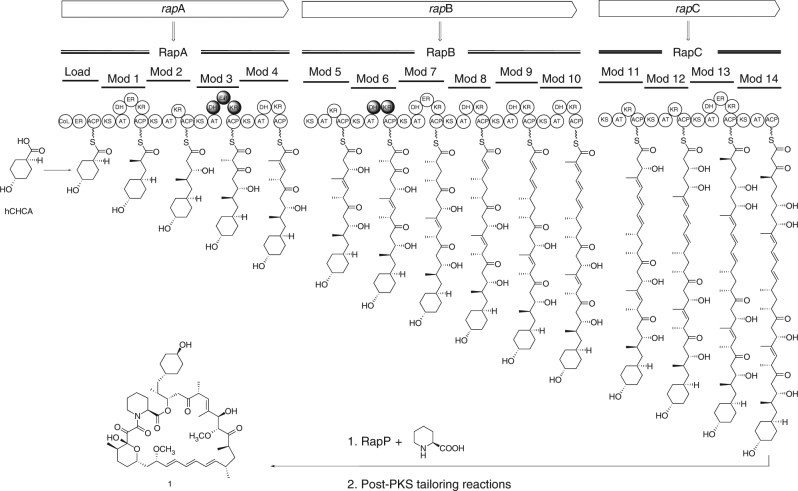
Pathway for the biosynthesis of 1 and organisation of the rapamycin PKS. The repetitive, modular nature of each PKS protein is clearly evident. The circles represent individual catalytic domains: CoL = CoA ligase like; KS = β-ketoacyl-ACP synthase; AT = acyltransferase; DH = dehydratase; ER = enoyl reductase; KR = β-ketoacyl-ACP reductase; ACP = acylcarrier protein. Filled circles represent inactive domains. hCHCA = *trans*-4-hydroxycyclohexanecarboxylic acid which is fed to the strain ISOM-4010 to initiate the biosynthesis of **1** and rapalogs **2**–**8**

Our initial target was to replace inactive domains from module 3 of the rapamycin PKS with active domains from module 11 and 13 to yield strains capable of producing 32-desketorapamycin. This experiment was performed using the rapamycin Δ*rapK* mutasynthesis strain *Streptomyces rapamycinicus* ISOM-4010 (previously BIOT-4010)^19^. This strain produces rapalogs only in the presence of exogenously added non-native starter units, for example feeding *trans*-4-hydroxycyclohexanecarboxylic acid (hCHCA) leads to production of 39-desmethoxyrapamycin, **1** (Fig. 1)^19, 20^. To carry out the experiment, we made use of the *Escherichia coli-Streptomyces* shuttle plasmid pKC1139^21^. This contains the temperature sensitive pSG5 replicon^22^, which enables a three-step protocol whereby the plasmid (containing the appropriate DNA to enable the desired domain replacement via an allelic replacement approach) is first conjugated into the actinomycete strain from *E. coli*. Maintenance of the apramycin resistant ex-conjugants at a higher, non-permissive temperature (at which the pSG5 replicon is essentially non-functional) then allows selection of strains where the plasmid has recombined via a region of homology into the target PKS gene. Finally, the strains are maintained at a lower (permissive) temperature allowing selection of apramycin sensitive colonies which have undergone a second recombination event to lose the plasmid. However, instead of the expected mix of domain replacement mutants and wild-type revertants, we discovered a variety of progeny producing unexpected products.

Following fermentation of these mutants, analysis of their culture extracts using LCMS/UV indicated a variety of rapamycin-like products that were only generated in the presence of exogenous hCHCA. All compounds exhibited the distinctive rapamycin UV chromophore but had *m/z* values inconsistent with the originally targeted rapalog. This phenomenon was readily reproducible and the mutants were stable. To quantify the distribution of phenotypes we repeated the experiment, using the same plasmid, and 667 progeny were isolated, fermented with hCHCA and their culture extracts analysed. Of these, 22.5% (150) produced new rapalogs, 63.1% (421) produced **1** and 14.4% (96) produced no obvious molecules with the rapamycin chromophore. Among the mutants producing new rapalogs, we identified twenty apparent variations on the rapamycin structure on the basis of HPLC retention times, *m/z* values and MS/MS fragmentation patterns.

**Fig. 2:**
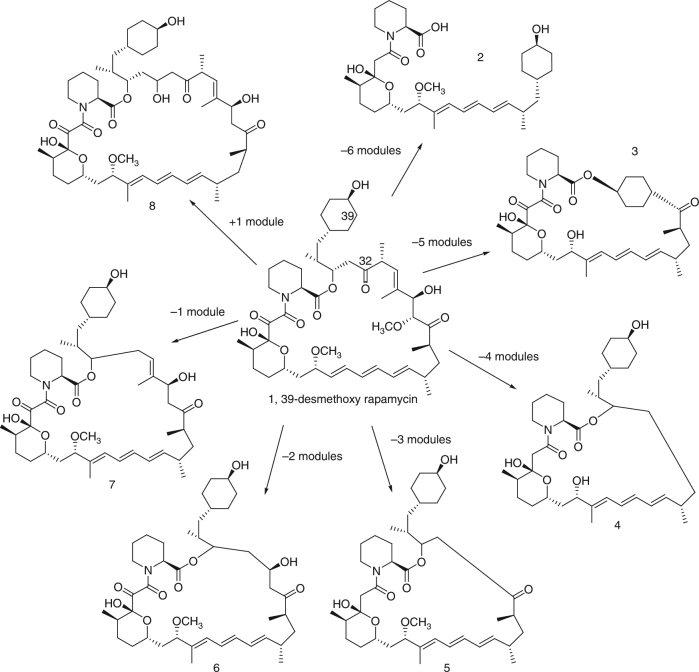
Structures of isolated rapalogs (2–8) encompassing a range of macrolactone ring sizes. C32 of rapamycin is labelled and is the site of the chemical change expected from the attempted rational bioengineering experiment via a reductive loop swap

Based on this analysis, the fermentation of seven mutants, each producing a different rapalog, was scaled up and the products isolated at the 10−100s of milligram scale. Their structures were then determined through a combination of 2D NMR and high-resolution LCMS/MS analysis (Supplementary Note 1). As shown in Fig. 2, these rapalogs include molecules with both truncated (**3**–**7**) and expanded (**8**) macrolactone rings, alternative cyclization patterns (**3**), and linear molecules (**2**). The structures were consistent with biosynthesis occurring on altered rapamycin PKSs consisting of reduced or increased numbers of extension modules. Given the tandem repetitive nature of genes encoding modular PKSs, this immediately suggested a recombination-based process in which the genes encoding the rapamycin PKS had undergone a range of deletion or duplication events.

To further probe the utility of these mutants, a selection of five, each generating a different rapalog, were fermented in the presence of hCHCA or one of 32 further starter unit acid analogues leading to the mutasynthesis of 100s of new rapalogs as ascertained by LCMS/UV analysis (Supplementary Note 2).

### Biological activity of new rapalogs

The unexpected observation that a single experiment could generate rapalogs of diverse chemical structure prompted us to investigate, first, whether these unique molecules might bind to a new target or show altered, yet valuable, biological activity. Selected rapalogs were first examined for their ability to inhibit both the peptidyl-prolyl isomerase (PPIase) inhibitory activity of the FK506-binding protein (FKBP12)^23^, using a well understood biochemical assay, and modulation of the mTORC1 pathway activity, via the validated inhibition assay of p-S6 formation^24^ (Table 1).

**Table 1:** Biological activity of rapalogs

**Compound ID**	**Module organisation**	**FKBP12** ***K***_**i**_ **(nM)**	**p-S6 IC**_**50**_ **(nM)**	***S. cerevisiae*** **MIC (μg mL** ^**−1**^ **)**
Rapamycin	Natural	5.4	<1	<0.25
**1**	Natural	2.5	N/A	<0.25
**8**	+1	6.5	N/A	<2
**7**	−1	7.9	100	<2
**5**	−3	370	300	<64
**3**	−5	460	>1900	>64
**2**	−6	>1000	1000	>64

Comparison of FKBP12 PPIase inhibition, mTORC1 pathway inhibition (p-S6) and *S. cerevisiae* MIC values for a selection of rapalogs.

As anticipated, the potency of mTORC1 pathway inhibition decreased as the size of the macrocycle ring was reduced. Despite all of the tested molecules retaining the triene moiety, which forms a key interaction with the FRAP domain of mTOR^25^, reducing ring size will significantly affect the triene geometry which is known to be critical for this interaction. However, despite a reduction in inhibitory activity of 3–4 log units, some of the rapalogs still retained sub-micromolar inhibitory activity, suggesting they may be useful for enabling more subtle modulation of mTORC1 functions.

In contrast, the less-immunosuppressive rapalogs retained potent inhibition of FKBP12 until six or more carbon atoms were removed from the macrocyclic ring. This is consistent with the observation that all of these compounds retain the key tricarbonyl region adjacent to the pipecolate moiety which, together, encompass the FK506-binding domain of rapamycin^25^. Based on these observations we examined the ability of AE-derived rapalogs to inhibit the FKBP12-like macrophage infectivity potentiator (MIP) proteins which are key virulence determinants for several pathogenic gram-negative bacteria including *Legionella pneumophila*, *Burkholderia pseudomallei* and *Coxiella burnetii* ^26–28^. These experiments identified **7** as a potent inhibitor (IC_50_ < 25 nM) of the PPIase activity of the MIP protein BPSS1823 of *B. pseudomallei* ^28^ (Table 1). Furthermore, **7** was shown to increase the survival of human macrophages challenged with *B. pseudomallei*.

We additionally assessed the rapalogs for antifungal activity using a standard *Saccharomyces cerevisiae* MIC assay. As expected, activity was similar to that observed for the mTORC1 pathway assay, likely through inhibition of TOR1 and TOR2.

### Genetic basis of new rapalog biosynthesis

We next sought to understand the genetic basis of the AE-generated chemical diversity. To achieve this, we subjected 17 mutants to whole-genome sequencing using either single-molecule real-time (SMRT) technology on the PacBio RSII platform, or through sequencing of TruSeq PCR-free shotgun and Nextera matepair libraries on an Illumina MiSeq platform. In order to understand variation in the putative recombination events, the 17 mutants chosen for sequencing included multiple examples producing each of the rapalogs **2**–**8** at a range of titres (Supplementary Table 1).

**Fig. 3:**
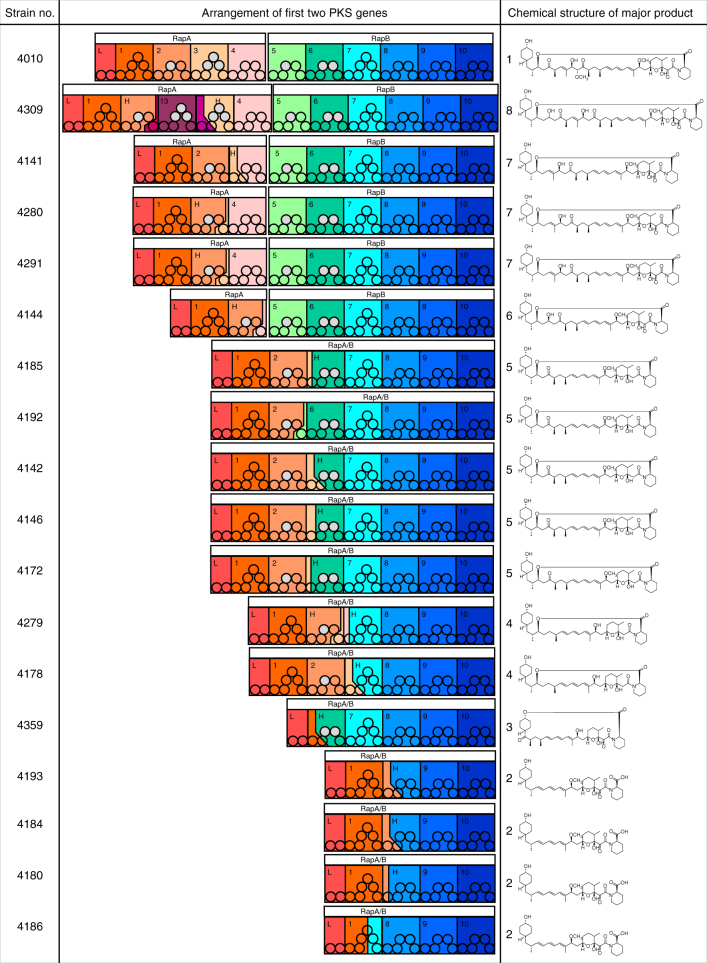
Comparison of the chemical structures isolated from each of the novel strains and the rapamycin PKS modular alignment for each of the sequenced PKS genes. Circles show enzymatic domains in each module. Modules 11–14 encoded by RapC are not shown

Analysis of the resulting assemblies revealed that any genomic changes were confined to the PKS region of the rapamycin BGC^29^. In each case, new PKS architectures observed were consistent with recombination between modules in regions of high sequence similarity, including both deletion and duplication events. In several examples an apparently simple deletion event had produced a new PKS-encoding gene, frequently via conflation of *rapA* and *rapB* genes, while in others the process was more complex (Fig. 3). For example, the strain producing the chain-extended product **8** (ISOM-4309) encodes a new PKS gene with almost two modules deleted (most of modules 2 and 3) while three extra modules (derived from part of module 11 and modules 12 and 13 from *rapC*) are inserted into *rapA*. None of the 17 sequenced PKS gene clusters had identical DNA sequences. In some cases, the overall architecture was similar, but with different junctions seen at the contraction point, for example comparison of ISOM-4180 and ISOM-4184, which have junctions 843 bp apart. In another case (ISOM-4291 and ISOM-4280), the 63 bp region of the likely junction was identical, although as both strains were progeny from different lines they had arisen through independent recombination events.

**Fig. 4:**
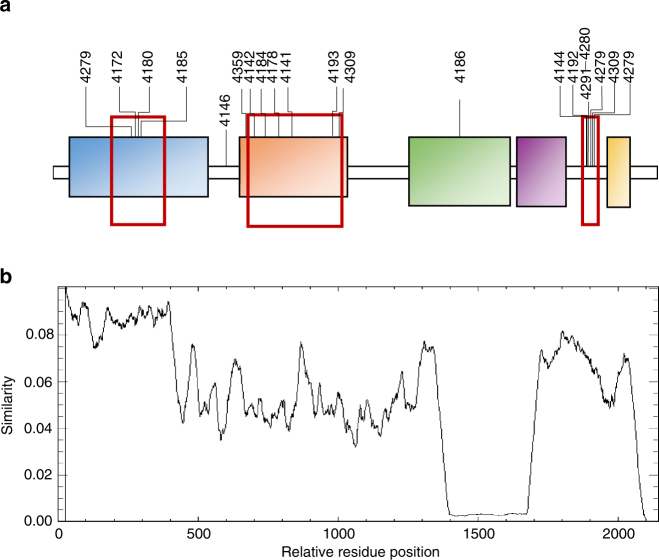
**a** Analysis of junctions at contraction regions found following analysis of the rapamycin PKS genes present in strains producing high titres of ring contracted rapalogs. Potential recombination hotspots are shown with red boxes, strain numbers are shown above the junctions. Domain boundaries corresponding to the Ketosynthase (KS), Acyltransferase (AT), Enoyl Reductase (ER), Ketoreductase (KR) and Acylcarrier Protein (ACP) are highlighted. **b** A similarity graph generated using Plotcon (http://emboss.bioinformatics.nl/cgi-bin/emboss/plotcon, generated via comparison of all of the modules in the native rapamycin PKS

On closer examination, it became clear that the inter-modular PKS junction sites of the higher-titre mutants were frequently located either internal to DNA coding for the ketosynthase (KS) or acyltransferase (AT) domains, or in a region just upstream of DNA coding for the acylcarrier protein (ACP) domain that corresponds to a flexible linker region (Fig. 4). Analysis of rapalog titres revealed that 14 of the 17 mutants had titres >60 μM, as compared to the mean titre of **1** (193.3 ± 49.2 μM) from control fermentations (Supplementary Table 1). One notable strain that fell outside this window was ISOM-4309 (11.7 ± 0.2 μM), the only strain producing a ring expanded product. Interestingly, the junctions which fall within the region encoding the AT domains join modules that both contain either malonate or methylmalonate encoding ATs. We anticipate that analysis of these and future AE-generated hybrid modules will provide valuable guidelines for the rational bioengineering of modular PKSs.

### Generality and mechanism of the AE process

Encouraged by these results, we attempted to replicate the AE process in a second modular PKS encoding BGC. For this we chose the tylosin PKS of *Streptomyces fradiae* NRRL 2702, as the organism is amenable to genetic modification and tylosin has a strong UV chromophore to aid preliminary chemical analysis.

To simplify the AE procedure, and in contrast to the rapamycin example, pKC1139-based plasmids containing single regions of homology (~ 2 kb) to each of the modules in the tylosin PKS were generated, rather than constructs designed to mediate an allelic replacement. We then carried out an analogous genetic procedure to that used for modification of ISOM-4010 above (Methods section) and identified progeny that produced tylosin-related analogues instead of tylosin as determined by LCMS/UV analysis. Following whole-genome sequencing (Illumina sequencing) we identified ten different examples of tylosin PKS rearrangements that were analogous to those observed for the rapamycin PKS, including deletion of modules 4−6 (in five cases), deletion of modules 1 to 4 (in two cases), deletion of modules 2−6 (in three cases), and a deletion between the loading domain and module 6 in one case (Supplementary Fig. 1).

At this point, we had come to believe that the presence of the temperature sensitive, high-copy replicon of pKC1139 was likely to be an essential part of the AE process. Therefore, we repeated the AE procedure in the tylosin system but included a control plasmid based on the suicide vector pKC1132^21^, which is similar to pKC1139 but lacks the pSG5-derived replicon. Both plasmids contained the same ~ 2 kbp fragment of DNA from module 2 of the tylosin PKS encompassing the KR and ACP domains. After performing the AE procedure with these plasmids in parallel, 67% of the progeny isolated after transformation with the pKC1139-based plasmid (*n* = 30) produced new tylosin analogues analogous to those described above. In contrast, when the pKC1132-based control plasmid was used, 70% (21) reverted to tylosin production and 30% (9) were non-producers; no progeny from the control experiment produced new tylosin analogues. These data lead us to conclude that the pSG5-based replicon of pKC1139 may be critical to the AE process we describe.

## Discussion

In nature, modular PKSs evolve via processes that comprise gene and intra-gene (e.g., module) duplications, speciation including mutation and homologous recombination (both inter-BGC and intra-BGC), and horizontal gene/BGC transfer events^18, 30–32^. Indeed, there are several families of known natural products, characterised by structurally related analogues comprising a range of macrocyclic ring sizes, for which the biosynthetic, evolutionary origins are likely to involve a process involving the deletion or duplication of PKS modules. Examples include the polyenes, marginolactones and macrodiolides^33^. Recently, Abe and co-workers examined the evolutionary relationship of giant modular PKS gene sets that encode for the biosynthesis of several structurally related aminopolyol polyketides (each between 25–30 modules)^34^. The authors concluded that, during evolution, the modular unit for duplication or deletion is likely to follow the non-canonical domain order AT through to KS, rather than the canonical KS through to AT. This should not be surprising given the knowledge that *trans*-AT PKSs (which do not have embedded AT domains), show a strong correlation between the chemical structure of the substrate produced by the processing enzymes of a module and the nature of the downstream KS domain^35, 36^. It is also consistent with observations of downstream KS selectivity made during PKS bioengineering^37, 38^ and morphing^39, 40^ experiments.

The process we report here mimics, on a laboratory timescale, a plausible mechanism of natural evolution for modular PKS genes involving recombination between separate biosynthetic genes or BGCs. We find that the majority of new PKSs formed are functional, and that the module junction sites for the most productive PKSs are not canonical but occur intra-module, generally within KS or AT sequences, or in the inter-domain linker region upstream of ACP domains. Given these considerations we refer to the laboratory induced process as Accelerated Evolution (AE). Moreover, it is important to stress that in a single experiment the AE process generates a multiplicity of new PKS-encoding progeny and therefore enables the diversity orientated biosynthesis of numerous new polyketide molecules.

Recombination and repair in *Streptomyces* bacteria are relatively poorly understood^41, 42^. It is our current hypothesis that an essential component of the AE process is localised genetic instability caused by activation of the pSG5 temperature sensitive replicon while still integrated into the genome. Lowering the temperature, so that it is permissive for function of the pSG5 replication, likely generates a new replication fork which can collide with the natural fork leading to temporary arrest. Such events can be catastrophic, and in bacteria and eukaryotes are often associated with genome instability and rearrangements^43–46^. Several mechanisms can act to resolve this kind of damage including homologous recombination and non-homologous end joining^42, 47, 48^. For the case in point, the highly repetitive nature of the flanking PKS genes provide the ideal template for repair via homologous recombination with concomitant loss of the genomic region encoding the pSG5 replicon. This mechanism is entirely consistent with the data obtained from sequencing the 17 rapalog producing mutants.

In summary, although the precise molecular mechanism of the AE process remains to be determined, our findings represent a new paradigm in the search for new and valuable chemical entities based on natural products. Experiments to extend the AE approach to many additional natural product biosynthetic pathways are in progress.

## Methods

### Bacterial strains and plasmids

*Streptomyces rapamycinicus* ISOM-4010 (previously BIOT-4010; *Streptomyces rapamycinicus* NRRL5491 Δ*rapK* ^19^), *S. fradiae* NRRL 2702, and their derivatives were maintained on MAM agar. The pKC1139-derived^21^ constructs able to induce deletion or expansion of the PKS modules were maintained in *E. coli* DH10B and transferred from *E. coli* ET12567/pUZ8002 to *S. rapamycinicus* ISOM-4010 or *S. fradiae* NRRL 2702 according to standard conjugal transfer methods^49^.

### Plasmid generation for *Streptomycesrapamycinicus*

The AE plasmids for the rapamycin PKS contained the particular regions of the gene cluster intended to act as homology regions for recombination and were amplified from Cos25^29^ (primers listed in Supplementary Table 2). Each of the amplified products was cloned into a separate pUC19 backbone. The first fragment was then excised using *Eco*RI and *Apa*I, and the second fragment using *ApaI* and *Xba*I. Both were then ligated with pUC19 between *Eco*RI and *Xba*I, to form an intermediate plasmid 1. The third fragment was excised using *Xba*I and *Pst*I, and the fourth using *Pst*I and *Hin*dIII, and ligated together with pUC19 between *Xba*I and *Hin*dIII sites. This plasmid was designated intermediate plasmid 2.

Intermediate plasmid 1 was digested with *Eco*RI and *Xba*I, to form the first region of homology. The second region of homology was retrieved from intermediate plasmid 2 through digestion with *Xba*I and *Hin*dIII. These two fragments were ligated together with pUC19 that had been digested with *Eco*RI and *Hin*dIII. The resulting vector was then digested with *Nhe*I and *Bgl*II to insert the desired reductive loop. Two alternative loops were used, one from module 13 of the rapamycin PKS, and one from module 11. The reductive loop of module 13 of the rapamycin cluster was excised from plasmid pPF137^50^ using *Nhe*I and *Bgl*II, and the reductive loop of module 11 was excised from plasmid pWV165^51^. The resulting cassettes were cloned into to pKC1139 between *Eco*RI and *Hin*dIII sites. The resulting plasmids were named pSGK210 (containing rapamycin PKS module 13 loop) and pSGK212 (containing rapamycin PKS module 11 loop). These plasmids were transferred to *S. rapamycinicus* ISOM-4010 as described below.

### AE procedure in *Streptomyces rapamycinicus*

Plates of *S. rapamycinicus* ISOM-4010 transformed^49^ with plasmids pSGK210 and pSGK212 were incubated at 37 °C until single exconjugant colonies were visible. Colonies were transferred to MAM agar (Wheat Starch, 10 g L^−1^; Corn steep powder, 2.5 g L^−1^; Yeast extract, 3 g L^−1^; CaCO_3_, 3 g L^−1^; FeSO_4_, 0.3 g L^−1^; Agar, 20 g L^−1^) containing apramycin (50 μg mL^−1^) and nalidixic acid (50 μg mL^−1^), and were incubated at 37 °C for 7 days. Approximately 10–15 of the strains were then transferred to solid ISP3 agar (Oatmeal, 20 g L^−1^; Bacto Agar, 18 g L^−1^; Trace element solution, 1 mL L^−1^ (1 g L^−1^ each of FeSO_4_·7H_2_O, MnCl_2_·4H_2_O and ZnSO_4_·7H_2_O)) lacking antibiotics and incubated at 37 °C for ~3 days before transferring to ISP3 agar for a further 3 or 4 days. A final round of subculture at 37 °C on ISP3 agar was performed before transferring again to ISP3 agar and incubating at 28 °C to allow the strain to sporulate (~10–14 days). Spores were harvested in 20% glycerol and a dilution series prepared in water and spread onto ISP3 agar before incubating at 28 °C. Once individual sporulating colonies were visible they were replica plated to ISP3 agar with and without apramycin to assess loss of plasmid. Strains that had lost the marker were then tested by fermentation, extraction and HPLC analysis as described below, to assess whether they produced **1** (i.e., had reverted to original strain). Among the strains that no longer produced **1**, strains producing new compound peaks with the rapamycin chromophore were identified.

### Generation of AE plasmids for use in *Streptomyces fradiae*

For tylosin AE experiments regions of the tylosin PKS were amplified from genomic DNA (isolated using FastDNA SPIN Kit for Soil—MP Biomedicals) using the primer pairs IR001_3 to IR001_8 listed in Supplementary Table 2. Each of the amplified products was introduced into pKC1139 which had been digested with *Hin*dIII and *Eco*RI using InFusion cloning (Clontech). Construct pIR001_3 contains a 2052 bp fragment amplified from to module 3 (including KS and AT domains). Construct pIR001_4 contains a 2002 bp fragment amplified from module 5 (including KR and ER/ACP domains). Construct pIR001_6 contains a 2091 bp fragment amplified from module 1 (including KR and ACP domains). Construct pIR001_7 contains a 2091 bp fragment amplified from module 3 (including KR and ACP domains). Construct pIR001_8 contains a 2070 bp fragment amplified from module 4, including KR and ACP domains. These plasmids were transferred to *S. fradiae* NRRL 2702 by standard conjugation methods as described below.

### AE procedure in *Streptomyces fradiae*

Plates of *S. fradiae* NRRL 2702 transformed^49^ with plasmids generated as described above, were incubated at 37 °C until single exconjugant colonies were visible. Colonies were transferred to MAM agar containing apramycin (50 μg mL^−1^) and nalidixic acid (50 μg mL^−1^), and were incubated at 37 °C for 7 days. Approximately 20 of the strains were then transferred to MAM agar with apramycin and nalidixic acid, and when stabilised were tested for production of tylosin to select primary integrants lacking tylosin production (as described below). The non-producers were transferred to MAM agar lacking antibiotics and incubated at 37 °C for ~7 days before transferring to MAM agar for a further 3 or 4 days. A final round of subculture at 37 °C on MAM agar was performed before transferring again to MAM agar and incubating at 28 °C to allow the strain to sporulate (~10–14 days). Spores were harvested in 20% glycerol and a dilution series prepared in water and spread onto MAM medium before incubating at 37 °C. When only the individual sporulating colonies were visible they were replica plated on MAM agar with and without apramycin to assess loss of plasmid. Strains that had lost the marker were tested by fermentation, extraction and HPLC as described below, to assess whether they still produced tylosin (i.e., had reverted to original strain). Among the strains that no longer produced tylosin, strains producing new compound peaks were identified using HPLC-UV and LCMS.

### Analytical fermentation of genetically engineered strains

Derivatives of *S. rapamycinicus* ISOM-4010 were grown for 10–14 days on ISP3 or MAM agar. An agar cylinder (5 mm in diameter) from each strain was used to inoculate a 50 mL Falcon tube containing RapV7 medium^19^ (7 mL), and incubated at 28 °C, 300 r.p.m. (2.5 cm throw) for 48 h. This seed culture (0.5 mL) was used to inoculate MD6 medium^19^ (7 mL) and the culture was incubated at 26 °C and 300 r.p.m. for 6 days; after 24 h each culture was supplemented with hCHCA (final concentration 2 mM).

Derivatives of *S. fradiae* were grown for at least 7 days on MAM agar and used to inoculate 7 mL modified TylB medium (beet molasses, 20 g L^−1^; corn meal, 15 g L^−1^; vegetable peptone, 9 g L^−1^; NaCl, 1 g L^−1^; soy flour, 13.5 g L^−1^; (NH_4_)_2_PO_4_, 0.4 g L^−1^; CaCO_3_, 2 g L^−1^; soybean oil, 30 g L^−1^; in deionised water) and incubated at 28 °C, 300 r.p.m. (2.5 cm throw) for 7 days.

### Chemical analysis of rapalogs

An aliquot of whole culture broth (0.5–0.8 mL) was mixed 1:1 with methanol and shaken vigorously for 30 min. The sample was centrifuged at 16,100×*g*, and the supernatant was analysed by HPLC with diode array detection. The HPLC system comprised an Agilent HP1100 equipped with a Phenomenex Gemini-NX C18 3 μm 110 Å reversed-phase column (150 × 4.6 mm) or Phenomenex Hyperclone 3 μm BDS C18 130 Å column (150 mm × 4.6 mm) heated to 50 °C. The gradient elution was from 55% mobile phase B to 95% mobile phase B over 10 min followed by isocratic elution at 95% mobile phase B for 2 min; flow rate 1 mL min^−1^. Mobile phase A was 10% acetonitrile:90% water, containing 10 mM ammonium acetate and 0.1% trifluoroacetic acid; mobile phase B was 90% acetonitrile:10% water, containing 10 mM ammonium acetate and 0.1% trifluoroacetic acid. Rapalogs were identified by the presence of the characteristic rapamycin triene chromophore centred on *λ* = 278 nm, and by LCMS (see below).

### LCMS

The HPLC system described above was coupled to a Bruker Daltonics Esquire 3000 electrospray mass spectrometer. Chromatography was achieved over a Phenomenex HyperClone C18-BDS column (150 × 4.6 mm, 3 μm particle size) or Phenomenex Gemini-NX C18 110 Å reversed-phase column (150 × 4.6 mm, 3 μm particle size). Samples were eluted at a flow rate of 1 mL min^−1^ with either gradient A: mobile phase A: mobile phase B (90:10) to (0:100) over 20 min; or gradient B:50 % mobile phase B to 100% mobile phase B over 10 min followed by an isocratic hold at 100% mobile phase B for 3 min. Mobile phase A was water containing 0.1% formic acid and mobile phase B was acetonitrile containing 0.1% formic acid. Positive/negative ion mode switching was used and spectra were acquired between *m/z* values of 500−1000.

### Larger-scale fermentation and rapalog isolation

Seed cultures were prepared by inoculating spore stocks at 0.05% (w/v) inoculum into 400 mL medium RapV7 in 2 L Erlenmeyer flasks fitted with foam plugs. After 48 h at 28 °C, 250 r.p.m. (2.5 cm throw) each seed culture was transferred into 15 L of medium MD6/5–1, pre-adjusted to pH 6.0–7.0 in a V7 Braun 22 L fermenter^19^. Fermentation was carried out for 6 days at 26 °C with initial agitation at 200 r.p.m., aeration rate at 0.5 V/V/M and dissolved oxygen (DO) level controlled with the agitation cascade at 30% air saturation. For production hCHCA dissolved in methanol (3–5 mL) was added to the production medium 24 h after inoculation to give a final concentration of 2 mM. After five further days the whole broth was centrifuged at 3300×*g* for 25 min. The cell pellet was removed from the centrifuge pots by mixing with acetonitrile and decanting into a 10 L glass Duran. Further acetonitrile was added to give a ratio of 2 vol. of solvent to 1 vol. of cells. The mixture was then stirred for 1 h using an overhead electric paddle stirrer at 600 r.p.m. After 1 h stirring was halted and the mixture allowed to settle under gravity for 15 min. The solvent layer was removed and a further 2 vol. of acetonitrile added to the remaining cells. This was stirred again to obtain a second extract. Any target compound in the supernatant fraction was recovered by adding an equal volume of ethyl acetate and stirring for 1 h in a glass container using an overhead electric paddle stirrer at 600 r.p.m. The organic solvent was then separated by centrifugation at 3300×*g* for 15 min. The combined extracts from both the cell pellet and, if required, clarified broth, were concentrated in vacuo to give a residual aqueous extract which was then back extracted into an equal volume of ethyl acetate. A second ethyl acetate extraction was performed if required. The ethyl acetate extract containing the target rapalogs was then concentrated in vacuo to yield a final crude extract sample. The crude extract was dissolved in 1:1 methanol/acetonitrile (v/v) and C18 reverse-phase silica added. The solvent was removed in vacuo and the silica added to a C18 reverse-phase silica open column (70 × 50 mm diameter) and the column eluted step-wise with 3:2 water/acetonitrile (600 mL), 1:1 water/acetonitrile (400 mL), 2:3 water/acetonitrile (v/v) (1000 mL). Fractions containing rapalogs were pooled and taken to dryness under reduced pressure.

Rapalogs were isolated by dissolving the enriched extract in methanol (5 mL) and separating the mixture by size-exclusion chromatography over Sephadex LH-20 (column dimensions 1000 × 30 mm diameter) eluting with methanol. Fractions containing rapalogs were combined and the solvent removed under reduced pressure. The residue was dissolved in a minimum volume of acetonitrile and adsorbed onto C18 reverse-phase silica. This was then added to a C18 silica column (100 × 30 mm diameter) and eluted with 3:2 (v/v) acetonitrile/water. The fractions containing compounds of interest were combined and taken to dryness under reduced pressure.

Alternatively, the dried crude extract was dissolved in ethyl acetate and normal phase silica gel 60 added. The solvent was removed in vacuo and the silica added to a silica gel 60 open column (300 × 25 mm diameter) and the column eluted with chloroform followed by an increasing methanol concentration to 5% in 1% steps. Fractions containing rapalogs were pooled and the solvent removed under reduced pressure. Final purification was effected by prep HPLC using a Gilson system with a Waters X Terra C18 column (19 × 250 mm, 10 μm particle size). Samples were chromatographed using a gradient of water and acetonitrile (50:50) to (0:100) over 25 min at a flow rate of 21 ml min^−1^. Fractions containing rapalogs were pooled and the solvent removed under reduced pressure.

### Mutasynthesis of rapalogs

A variety of carboxylic acid feeds were added to the fermentation broths^19^. The starter units tested were: cyclohexanecarboxylic acid, cyclohex-1-enecarboxylic acid, cyclohex-3-enecarboxylic acid, 3-methoxycyclohexanecarboxylic acid, ethyl 5-hydroxycyclohex-3-enecarboxylic acid, (1s*,3S*,4R*)−4-fluoro-3-hydroxycyclohexanecarboxylic acid, 4-methylcyclohexanecarboxylic acid, cyclopentanecarboxylic acid, cycloheptanecarboxylic acid, (1R*,2S*,4S*)-bicyclo[2.2.1]heptane-2-carboxylic acid, (1S*,2R*,5R*,6S*)−2-hydroxybicyclo[3.2.1]octane-6-carboxylic acid, tetrahydro-2H-pyran-4-carboxylic acid, tetrahydro-2H-thiopyran-4-carboxylic acid, 3-hydroxybenzoic acid, 4-methylthiophene-2-carboxylic acid, 3-amino-5-hydroxybenzoic acid, 4-hydroxy-3,3-dimethylcyclohexanecarboxylic acid, 4-methylenecyclohexanecarboxylic acid, 4-methylcyclohex-3-enecarboxylic acid, (1S*,4S*)−4-methylcyclohexanecarboxylic acid, (1S*,3S*,4S*)−3,4-dihydroxycyclohexanecarboxylic acid, 3-methylcyclohexanecarboxylic acid, isonicotinic acid, 5-methylthiophene-2-carboxylic acid, cyclohexanecarboxylic acid, (1R*,4R*)−4-hydroxycyclohexanecarboxylic acid, (2S*)-bicyclo[2.2.1]heptane-2-carboxylic acid, (1S*,3S*)−3-hydroxycyclohexanecarboxylic acid, (1S*,3R*,4S*)-methyl 3-fluoro-4-hydroxycyclohexanecarboxylate, (1S*,3R*,4S*)−3-ethyl-4-hydroxycyclohexanecarboxylic acid, methyl 3,3-difluoro-4-hydroxycyclohexanecarboxylate, (1S*,3R*)−3-hydroxycyclohexanecarboxylic acid. These were either purchased from Sigma, Alfa Aesar, Fisher Scientific or synthesised using standard methods (Supplementary Note 2).

### Analysis of temperature sensitive replicon importance for AE

To test the importance of the temperature sensitive replicon for recombination, conjugative plasmids containing identical regions of homology to the tylosin PKS genes (a 2035 bp fragment covering the KR and ACP domains of module 2) were generated for pKC1139 and the closely related suicide vector pKC1132 which lacks the pSG5 replicon^21^ and both were used for AE of the tylosin PKS. Following the AE procedure with the pSG5-based plasmid, 67% (20) of the isolated progeny produced new peaks assessed to be new tylosin analogues by LCMS/UV methods described above, and 33% (10) lacked tylosin or analogue production. When the control plasmid was used, no strains produced new tylosin analogues. Instead, 70% (21) reverted to tylosin production (wild-type) and 30% (9) were non-producers; none produced any tylosin analogues.

### FKBP12 PPIase inhibition assay

The assay was conducted at 10 °C in 50 mM Tris buffer at pH 8.0, 50 µM DTT, 100 mM NaCl, 0.005% NP40 with 6 nM FKBP12 and 60 µM substrate (SUC-ALPF-pNA, diluted from 20 mg mL^−1^ stock in 0.5 M LiCl/TFE). The *K*_m_ for the substrate was determined to be ~188 µM. The first order rate equation was fitted to the absorbance data to obtain a rate constant. A catalytic rate (*K*_enz_) was calculated from the enzymatic rate minus the background rate. *K*_enz_ vs. inhibitor concentration was plotted to obtain the *K*_i_ value. All compounds were assayed at 6 concentrations in duplicate. The assay was conducted at Selcia Ltd, Ongar UK (http://www.selcia.com/sites/default/files/Selcia_Prolyl_Isomerases_Paper_2015.pdf).

### mTORC1 inhibition measurement

A commercially available Cellular RpS6 phospho-S6RP (Ser 235/236) HTRF assay (Homogeneous Time Resolved Fluorescence or TR-FRET assay; Cisbio, Codolet, France) was used according to the manufacturer’s instructions. Briefly, genome-edited human TSC2^(−/−)^ embryonic neural stem cells, were grown in 384-well plates, coated with p-Ornithin and laminin, at 6000 cells per well. Plates were incubated overnight at 37 °C and 5% CO_2_, serum starved for 5 h, and treated with compounds for 1 h at 37 °C and 5% CO_2_. Compounds were serially diluted 2.5-fold, for a 16 point dose-response with a range from 100 µM to 671 pM. Cells were lysed using 10 μL of 4X assay lysis buffer for 1 h at room temperature, and 8 μL of the total lysate was transferred to a white 384-well assay plate (Costar 3825). After the addition of both d2-labelled anti-S6 and cryptate labelled anti-phospho-S6 antibody to each well, the mixtures were incubated at room temperature for 3 h. Emission levels at 620 and 665 nm were measured using a Paradigm multilabel plate reader (Molecular Devices). FRET ratios were determined by calculation of emission ratios (665 nm/620 nm).

### *Burkholderia pseudomallei* PPIase inhibition assay

The PPIase activity of recombinant BPSS1823 protein was determined by a protease-coupled assay^28^. For inhibition measurements, recombinant BPSS1823 protein was preincubated with various concentrations of test article from 100 to 1 nM for 6 min prior to the addition of substrate. At least three independent readings were taken at each data point.

### *Saccharomyces cerevisiae* MIC assay

Minimum Inhibitory Concentration (MIC) values for each compound were determined using the microdilution method. Briefly, stock solutions of the 6 test rapalogs (6.4 mg mL^−1^ in methanol) were diluted 1:50 in RPMI 1640 broth (Sigma-Aldrich) to a working concentration of 128 µg ml^−1^, and serially diluted in broth (1:2) in sterile, polypropylene 96-well plates, 100 µL per well. *Saccharomyces cerevisiae* was cultured in RPMI 1640 broth, 37 °C, 250 r.p.m., 18 h. The culture was diluted in broth to achieve a cell density of approx. 106 CFU/mL. A volume of 100 µL aliquots of this dilution were added to each well and mixed by gentle trituration. Plates were incubated at 37 °C for 24 h. The concentration of compound at which no visible growth was apparent was determined to be the MIC. For concentrations at which growth was markedly reduced but not completely inhibited the microorganism was recorded as being susceptible.

### Assessment of purity for isolated rapalogs

Purified compounds were analysed using HPLC-UV/MS and two independent chromatography systems. Purity was assessed by LCMS and by UV absorbance at multiple wavelengths (210, 254 and 278 nm). All compounds were >95% pure at all wavelengths. Purity was finally confirmed by inspection of the ^1^H and ^13^C NMR spectra.

### NMR structure elucidation methods

NMR spectra were recorded on a Bruker Advance 500 spectrometer at 298 K operating at 500 MHz and 125 MHz for ^1^H and ^13^C, respectively. Standard Bruker pulse sequences were used to acquire ^1^H−^1^H COSY, APT, HMBC and HMQC spectra. NMR spectra were referenced to the residual proton or standard carbon resonances of the solvents in which they were run.

### Sequencing and analysis of recombinant PKS clusters

*S. rapamycinicus* strains were cultured in TSB medium and *S. fradiae* were cultured in CSM medium (30 g/L tryptic soy broth, 3 g/L yeast extract, 2 g/L MgSO_4_, 5 g/L glucose and 4 g/L maltose). The high molecular weight total genomic DNA was isolated using the salting out procedure^49^.

PacBio RSII sequencing was carried out as a service by the Earlham Institute (Norwich Research Park, Norwich, UK). Libraries were prepared using Pacific Biosciences DNA Template Prep Kit 3.0. Library size selection was carried out using MagBead OneCellPerWell v1. Sequencing was carried out on the PacBio RSII platform, using P6-C4 chemistry, and using 4 SMRT cells for each strain.

TruSeq PCR-free shotgun and Nextera matepair libraries were constructed from high molecular weight genomic DNA, using the manufacturer’s protocol. Sequencing was carried out on an Illumina MiSeq platform using the Illumina V3 600 cycle kit run in 2 × 300 bp mode. Eight libraries were multiplexed in a single run. Reads were processed using an in-house Illumina adapter trimming tool (fastq_miseq_trimmer). Read pairs were then pre-assembled using FLASH v1.2.11 (https://ccb.jhu.edu/software/FLASH/). Nextera matepair linker sequence was used for splitting of matepair reads (fastq_miseq_trimmer).

### Recombinant cluster assembly from Illumina reads

Both mapping and de novo assemblies were used to obtain a reference sequence for the recombinant PKS clusters, using the reads from Illumina platform. Processed reads were assembled de novo using Newbler v3 (Roche). Several assemblies were carried out using either all or subsets of the input data set, and the best assembly was selected using a score calculated from scaffold N50, edge and total number of contigs. Mapping to the cluster reference was carried out using Newbler v3 and the results were saved in ace and bam formats. At least two mapping projects (PCR-free and PCR-free + Nextera matepair reads) were created for each consensus version.

The mapping results were post-processed by an in-house ballfix program (to fix the paired reads metadata in the phd.ball file) and visualised using consed v29 software [www.phrap.org]. Copy number variations were visually detected using consed’s assembly view as changes in the coverage of PCR-free reads. Each consensus was reconfirmed by a final round of mapping.

### Recombinant cluster assembly from PacBio reads

PacBio reads ware assembled de novo using SMRT analysis pipeline v.2.2 (https://github.com/PacificBiosciences/SMRT-Analysis) with a single pass of consensus polishing using Quiver.

### Annotation of the recombinant clusters

Prediction of PKS/NRPS domains was carried out using an in-house version of the secmetdb pipeline (https://sourceforge.net/projects/secmetdb/). The DNA sequence of the domains was exported into the NCBI BLAST database and used for annotation of the domains in the recombinant strains using (annot_blast_mapper) and NCBI blastall v2.2.17. Open reading frame annotation was transferred from the reference parent cluster using the RATT pipeline (http://www.sanger.ac.uk/science/tools/pagit).

All sequences have been submitted to Genbank and are available under the accession numbers KY457762, KY457763, KY457764, KY457765, KY457766, KY457768, KY457769, KY457770, KY457771, KY457772, KY457773, KY457774, KY457775, KY457776, KY457777, KY457778, KY457779, KY457780 KY650706, KY650707, KY650708, KY650709, KY650710, KY650711, KY650712, KY650713, KY650714 and KY650715.

### Detection of recombination hotspots

Detection of potential recombination sites was carried out using the (annot_recomb_mapper) pipeline based on NCBI blastn v.2.2.17, run in master/slave mode. The results were saved in the Artemis feature format (*.tab) and combined with all other annotation information. Final junctions were defined as regions of the translated hybrid rapamycin or tylosin PKS genes where the amino acid sequence upstream of the junction had identity to one module but the downstream sequence had at least two amino acids which were not identical to that module, but were identical to the amino acid sequence from another non-sequential module of the PKS. Annotation results were combined in EMBL format (tab2embl) and visualised and manually curated in Artemis (http://www.sanger.ac.uk/science/tools/artemis).

### Data availability

The authors declare that the data supporting the findings reported in this study are available within the article and the Supplementary Information, or are available from the authors upon reasonable request. New nucleotide sequence data have been deposited in NCBI GenBank under the accession codes: KY457762, KY457763, KY457764, KY457765, KY457766, KY457768, KY457769, KY457770, KY457771, KY457772, KY457773, KY457774, KY457775, KY457776, KY457777, KY457778, KY457779, KY457780 KY650706, KY650707, KY650708, KY650709, KY650710, KY650711, KY650712, KY650713, KY650714 and KY650715.

## Electronic supplementary material

Supplementary Information
