# Supplementary material for: Diversity oriented biosynthesis via accelerated evolution of modular gene clusters
Source: Nat Commun. 2017 Oct 31;8:1206. doi: 10.1038/s41467-017-01344-3 (PMC5663706; doi:10.1038/s41467-017-01344-3)
Supplement: Supplementary file 1 — Supplementary Information [file 41467_2017_1344_MOESM1_ESM.pdf]

Supplementary Figure 1 | Genetic architecture of recombined tylosin PKS genes after AE process.

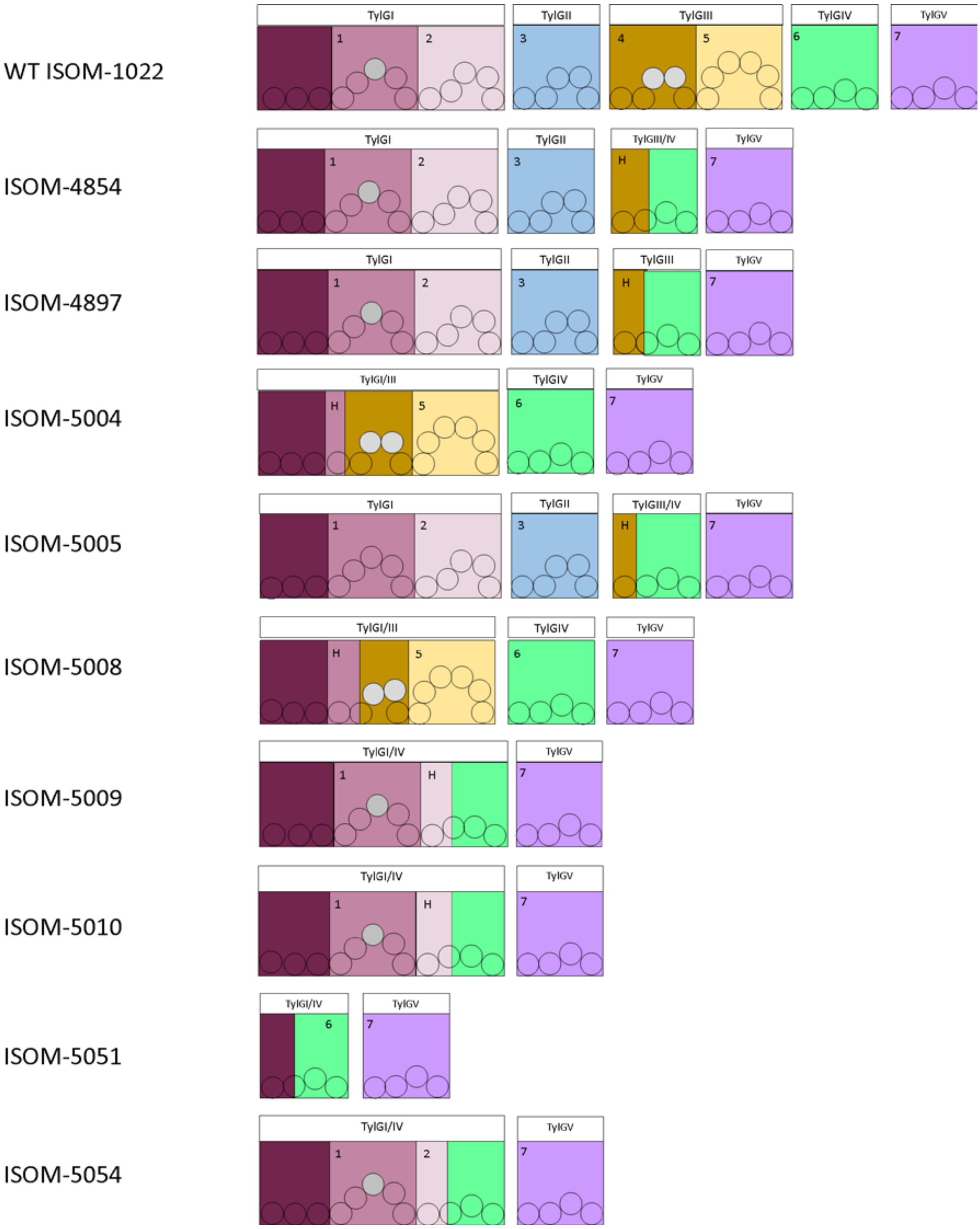

Supplementary Table 1 | Rapalog fermentation titres. The mean titre for production of **1** by strain ISOM-4010 fed with hCHCA (2 mM) was determined by analysing the raw data from 396 individual analytical fermentations carried out in Falcon tubes (see Methods section). Outliers were rejected based on a Jarque-Bera test. This analysis gave a mean value of 193.4 ± 49.2 μM. All raw data (titres) were calculated using HPLC-UV analysis with compound **1** as a standard.

**Strain**	**Rapalog produced**	**Module genotype**	**Rapalog titre (μM)**
ISOM-4010	**1**	Wild-type	193
ISOM-4309	**8**	Plus 1	9
ISOM-4141	**7**	Minus 1	121
ISOM-4280	**7**	Minus 1	177
ISOM-4291	**7**	Minus 1	197
ISOM-4144	**6**	Minus 2	87
ISOM-4185	**5**	Minus 3	73
ISOM-4192	**5**	Minus 3	77
ISOM-4142	**5**	Minus 3	70
ISOM-4146	**5**	Minus 3	7
ISOM-4172	**5**	Minus 3	63
ISOM-4279	**4**	Minus 4	84
ISOM-4178	**4**	Minus 4	37
ISOM-4359	**3**	Minus 5	109
ISOM-4193	**2**	Minus 6	126
ISOM-4184	**2**	Minus 6	135
ISOM-4180	**2**	Minus 6	149
ISOM-4186	**2**	Minus 6	126

Supplementary Table 2 | Oligonucleotides used in this study

Oligonucleotide sequence	PCR Product
CGACGAATTCCATCGCGCCCCGGCCCGCCAGG	1
TTGTCCGGCCGGGTGTCGTACGTCTTCGG	1
CCAGGGACGAGGAGCACGCCGTGTCCATCG	2
GGGGTGTAGAGGCTAGCCGCCCTGGCACCGGCCGAGC	2
GTATCTAGAAAGATCTAGTACCCGGGTTGTGGCGGTGCCGAGG	3
TCAGGCCGCCTCGGGCGTGTCGGTTGTCATCAAGATGG	3
GACGGCTCATCCACGTGCAGGGTGCGGGGAACC	4
GTCTAAGCTTTCCCCACCGACCGTGGCTGGGACGTCG	4
CGCGAATTCGGAGAAACCGGCACCGTCCGCACTGTCCGC	5
GGGGTGTAGAGGCTAGCCGCCCTGGCACCGGCCGAGC	5
CGTAAAGCTTGGAGACGACACCGTCACCGGCACCGCTGTG	6
GTATCTAGAAAGATCTAGTACCCGGGTTGTGGCGGTGCCGAGG	6
GGCCAGTGCCAAGCTTCCTTCATCGAGGAGCAGCTTTG	IR001_3
ACATGATTACGAATTCGGTGAAGATGCGGTTCGAG	IR001_3
GGCCAGTGCCAAGCTTCTGTCGCTCGCGCCC	IR001_4.F
ACATGATTACGAATTCGGGGTCGGGTGGT	IR001_4.R
GGCCAGTGCCAAGCTTCCGCGACGACCACCCA	IR001_6
ACATGATTACGAATTCCCGGTCGAGCAGCAGTCT	IR001_6
GGCCAGTGCCAAGCTTCCTGCACACCGCGG	IR001_7
ACATGATTACGAATTCTTCGGCGCCGGGGA	IR001_7
GGCCAGTGCCAAGCTTGAGGTCAGTGCCCACCCG	IR001_8
ACATGATTACGAATTCGCAGATGCCGGGCGAGG	IR001_8

Supplementary Note 1 | Rapalog isolation and structure elucidation

General. NMR spectra were recorded on a Bruker AVANCE 500 spectrometer at 298 K operating at 500 MHz and 125 MHz for ^{1}H and ^{13}C respectively. Standard Bruker pulse sequences were used to acquire ^{1}H-^{1}H COSY, APT, HMBC and HMQC spectra. Chemical shifts are reported in parts per million (ppm) relative to the solvent residual peak of chloroform-d_1 (^{1}H: 7.24 ppm, singlet; ^{13}C: 77.00 ppm, triplet) or acetonitrile-d_3 (^{1}H: 1.94 ppm, quintet; ^{13}C: 118.26 ppm, singlet).

COMPOUND 1

Compound **1** was reported previously[1] and authentic standards were used for LCMS analysis including quantification.

COMPOUND 2

The fermentation was as described in the Methods section except that the vegetative cultures (seed cultures) were prepared using 12 x 5 mm plugs from an agar plate and inoculating into 400 mL RapV7 medium in 2 L Erlenmeyer flasks with foam plugs. Cultivation was carried out for 48 h at 28°C, 250 rpm (2.5 cm throw), before fermentation as set out in the text.

Strain: ISOM-4180

Exogenous feed: 4-*trans*-hydroxylcyclohexanecarboxylic acid (final concentration 2 mM)

Volume: 1 x 15 L

DSP was as described in the text, both the cell mass and clarified broth were extracted and the crude extracts combined.

The crude extract (13.8 g) was dissolved in 1:1 methanol/acetonitrile and C18 reverse-phase silica added (26 g). The solvent was removed under reduced pressure and the silica added to a C18 reverse-phase silica open column (70 mm x 50 mm diameter) and the column eluted with 3:2 water/acetonitrile (600 mL), 1:1 water/acetonitrile (400 mL), 2:3 water/acetonitrile (1000 mL). Fractions containing **2** were combined and taken to dryness (6.0 g).

Compound **2** was then purified by dissolving the 6.0 g enriched extract in methanol (5 mL) and separating the mixture by size-exclusion chromatography over sephadex LH-20 resin (column dimensions 1000 mm x 30 mm diameter) eluted with methanol. Fractions containing **2** were combined and the solvent evaporated under reduced pressure. The material (210 mg) was adsorbed onto C18 reverse-phase silica (dissolved in 20 ml methanol, 2 g C18 silica added and the solvent removed under reduced pressure). This was then added to a C18 silica column (100 mm x 30 mm diameter) and eluted with 3:2 acetonitrile/water. The fractions containing **2** as adjudged by analytical HPLC (see methods section) were combined and the solvent evaporated under reduced pressure to yield **2** as a white, amorphous solid.

LC-MS analysis. RT = 10.7 min, *m/z* = 584.2 ($[M+Na]^{+}$) and 560.2 ($[M-H]^{-}$).

HRMS (*m/z*):, calcd for $C_{33}H_{51}NO_8$, Observed: 598.3731; calculated: 598.3714; Δ = 2.84 ppm.

Supplementary Figure 2 | Chemical structure and 2D NMR correlations for compound 2.

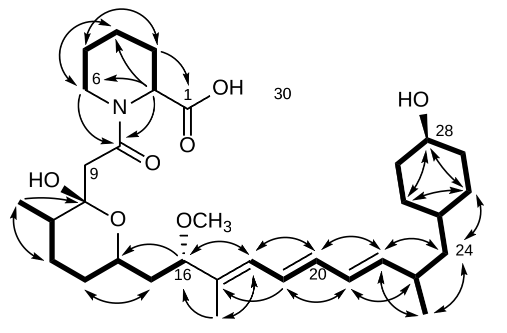

2

Supplementary Table 3 | NMR data for compound 2 in $CDCl_3$ at 500 MHz for ^{1}H and 125 MHz for ^{13}C. For methylene groups the axial and equatorial ^{1}H has not been assigned.

Position	^{1}H-NMR			^{13}C-NMR	HMBC correlations
	δ ppm	Multiplicity, *J* in Hz	COSY	*δ* ppm	^{1}H to ^{13}C
1	-	-	-	174.0	-
2	5.40	br. d, 4	H-3	51.5	C-1,C-3,C-4,C-6, C-8
3	1.66<br>1.22	m<br>m	H-2,<br>H-4	27.4	C-1,C-2,C-4, C-5
4	1.69<br>1.15	m<br>m	H-3,<br>H-5	21.1	C-2,C-3,C-5, C-6
5	1.69<br>1.45	m<br>m	H-4,<br>H-6	25.3	C-3,C-4, C-6
6	3.77<br>3.26	br.d, 12.8<br>ddd, 12.8, 9.5, 6	H-5	44.1	C-2,C-4,C-5, C-8
7	-	-	-		-
8	-	-	-	172.8	-
9	1.19<br>1.09	d, 16.5<br>d, 16.5	-	44.2	C-8, C-10, C-11
10	-	-	-	98.0	-
11	2.26	ddq, 11.5, 4, 6.5	11-CH_3,<br>H-12	34.3	C-9,C-10,C-12,C-13, 11-CH_3
11-CH_3	0.93	d, 6.5	H-11	16.8	C-10,C-11, C-12
12	1.46	m	H-11,<br>H-13	26.7	C-10,C-11,C-13,C-14, 11-CH_3
13	2.14<br>1.75	m<br>m	H-12,<br>H-14	30.8	C-11,C-14,C-15
14	4.13	m	H-13,<br>H-15	71.2	-
15	2.70<br>1.47	ddd, 16, 9.2, 11<br>ddd, 16,5.5, 6	H-14,<br>H-16	39.2	C-13,C-14,C-16, C-17

16	4.26	dd, 9.2, 5.5,	H-15, H-17	66.0	C-14,C-15,C-17,C-18
16-OCH_3	3.14	s	-	50.3	-
17	-	-	-	134.6	-
17-CH_3	1.72	s	-	10.2	C-16, C-17, C-18
18	6.07	d, 14.5	H-17, H-19	126.2	C-16,C-17,C-19,C-20, 17-CH_3
19	6.32	dd, 14.5, 11.4	H-18, H-20	133.4	C-17,C-18,C-20, C-21
20	6.15	dd, 14.5, 11.4	H-19, H-21	128.9	C-18,C-19,C-21, C-22
21	6.06	dd, 14.5, 14.4	H-20, H-22	133.5	C-19,C-20,C-22, C-23
22	5.50	dd, 14.4, 8	H-21, H-23	141.0	C-20,C-21,C-23, 23-CH_3, C-24
23	1.91	m	H-22, 23-CH_3, H-24	34.0	C-21,C-22,C-24,C-25, 23-CH_3
23-CH_3	0.93	d, 6.5	H-23	21.0	C-22,C-23, C-24
24	1.55	m	H-23, H-25	44.0	C-22,C-23,C-25,C-26, 23-CH_3, C-30
25	2.57	m	H-24, H-26, H-30	39.1	C-23,C-24,C-26,C-27, C-29, C-30
26	1.74	m	H-25, H-27	31.6	C-24,C-25,C-27,C-28, C-30
27	1.19	m	H-26, H-28	35.1	C-25,C-26,C-28, C-29
28	3.50	dddd, 10.8, 8.4, 6.8, 6.8	H-27, H-29	71.1	-
29	1.31	m	H-28, H-30	35.2	C-25, C-27, C-28, C-30
30	1.32	m	H-29, H-25	31.7	C-24,C-25,C-26,C-28, C-29

Supplementary Figure 3 | ^{1}H NMR spectrum ($CDCl_3$, 500 MHz) of compound 2.

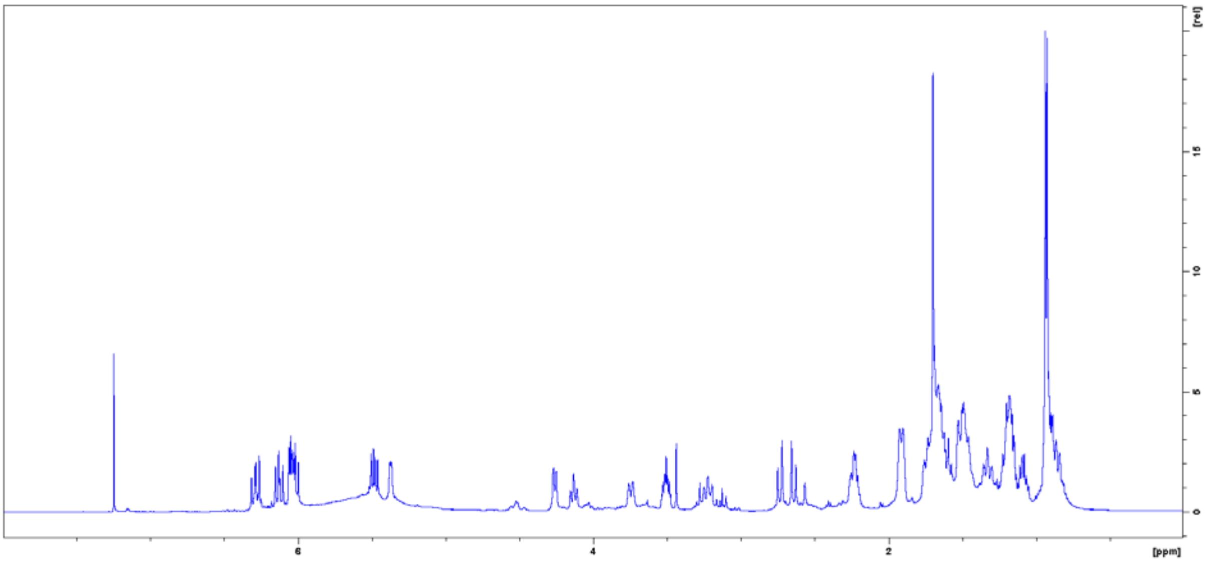

Supplementary Figure 4 | ^{13}C NMR spectrum ($CDCl_3$, 125 MHz) of compound 2.

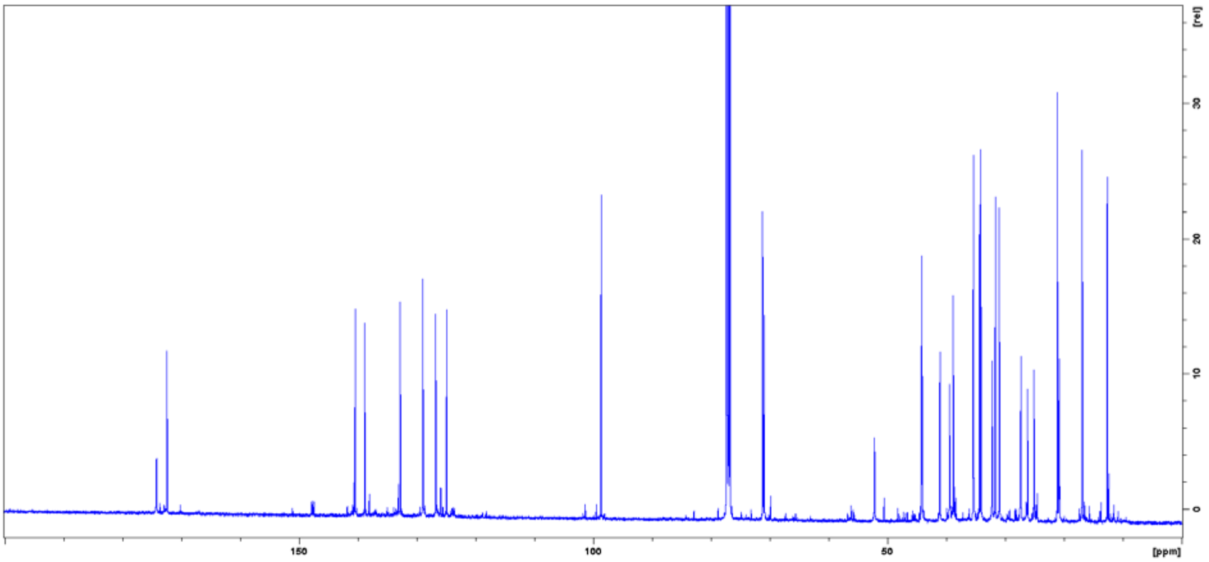

Supplementary Figure 5 | H,H-COSY spectrum of compound 2.

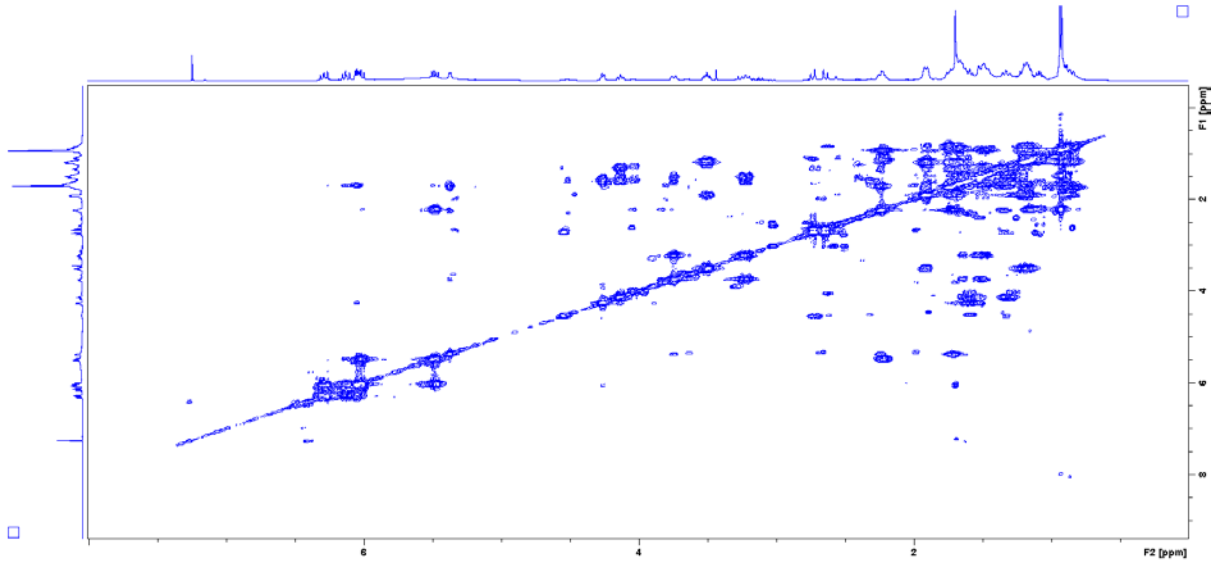

Supplementary Figure 6 | HSQC spectrum of compound 2.

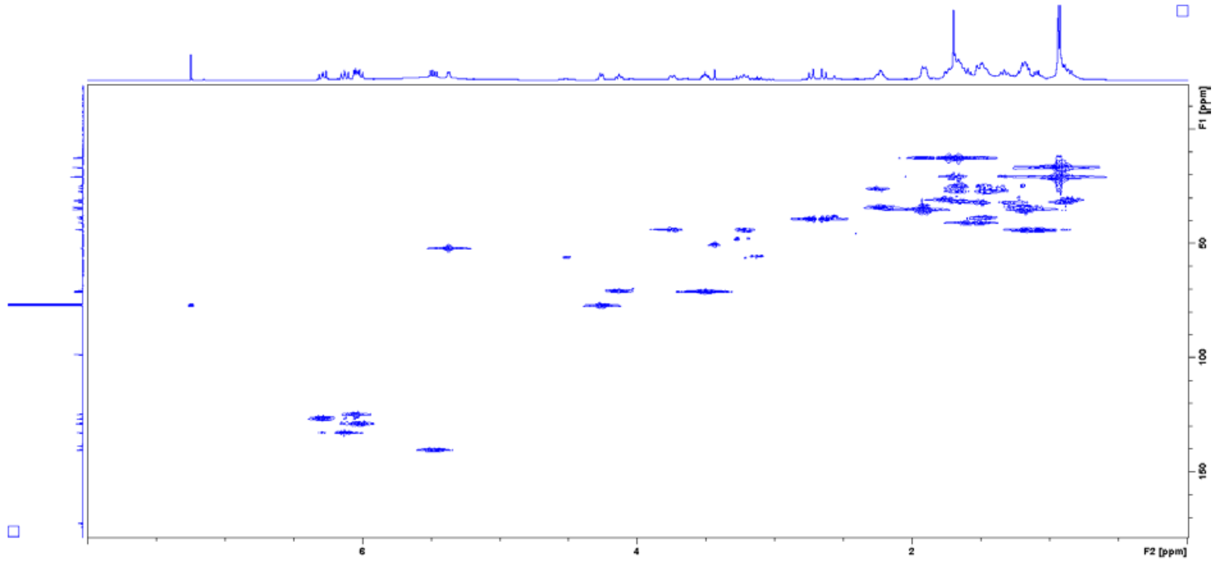

Supplementary Figure 7 | HMBC spectrum of compound 2.

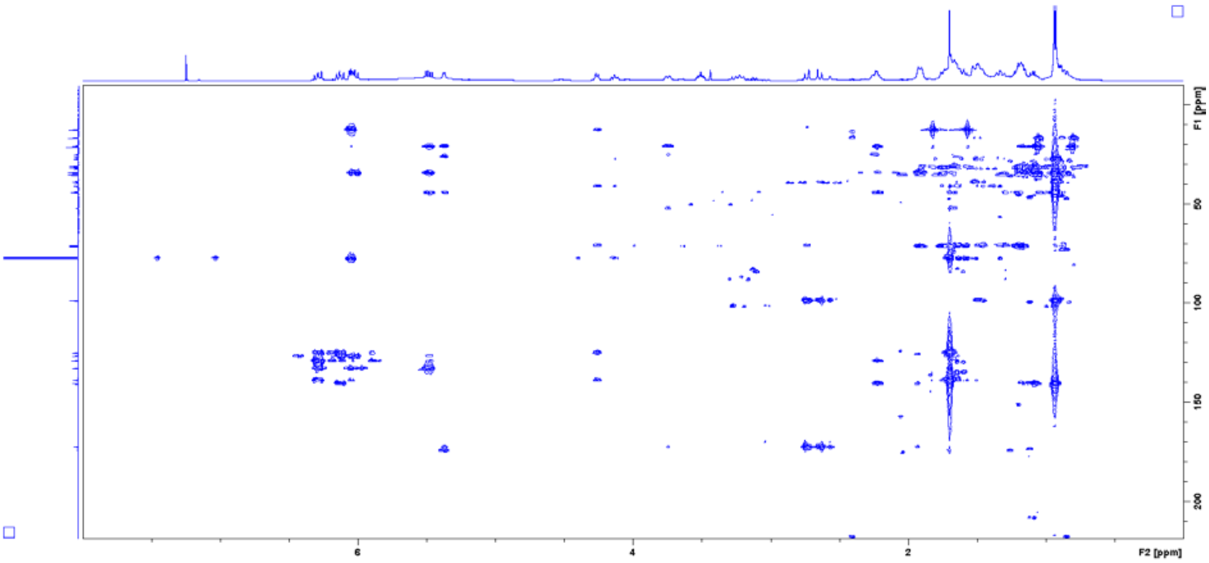

COMPOUND 3

The seed and production fermentations were performed as detailed in the methods section.

Strain: ISOM-4359

Feed: 4-*trans*-hydroxycyclohexanecarboxylic acid (hCHCA) (final concentration 2 mM)

Volume: 1 x 15 L fermentation

The resulting fermentation broth was separated by centrifugation and the cell paste and clarified broth processed as described for **2**.

The crude extract (20.7 g) was dissolved in methanol and silica added. The solvent was removed under reduced pressure and the adsorbed silica added to a flash silica column (20 cm x 5 cm diameter) and eluted with the 40:60 ethyl acetate/hexanes and then 50:50, 60:40, 70:30, 80:20, 90:10 mixtures followed by 100% ethyl acetate. Fractions containing **3** were combined and the solvent evaporated under reduced pressure to yield an enriched extract of 0.96 g.

Compound **3** was further purified by preparative HPLC on a Phenomenex Gemini NX C18 column: 10 µm, 21 mm diameter x 150 mm. Mobile phase A = water, B = acetonitrile; t = 0 min, B = 60%; t = 30 min, B = 100%). Flow rate 20 mL min^{-1}. Fractions containing **3** were defatted (dissolved in 80% aqueous methanol and extracted into hexanes; the solvent was then removed from the aqueous methanol layer to reveal the target compound) to yield a white amorphous solid (164 mg).

LC-MS analysis: RT = 14.9 min; *m/z* = 636.2 ($[M+Na]^+$) and 612.2 ($[M-H]^-$).

HRMS (*m/z*):, calcd for $C_{35}H_{51}NO_8Na$, 636.3512; found, 636.3516; Δ = 0.6 ppm.

Supplementary Figure 8 | Chemical structure and 2D NMR correlations for compound 3.

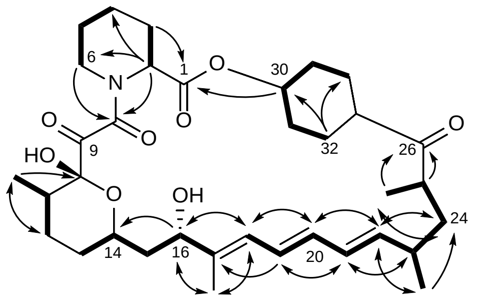

3

Supplementary Table 4 | NMR data for compound 3 in $CDCl_3$ at 500 MHz for ^1H and 125 MHz for ^{13}C.

Position	^1H-NMR			^{13}C-NMR	HMBC correlations
	δ ppm	Multiplicity, *J* in Hz	COSY	*δ* ppm	^1H to ^{13}C
1	-	-	-	168.7	-
2	5.16	d, 5.1	H-3	51.8	C-1, C-3, C-4, C-6, C-8
3	1.61<br>2.26	m<br>m	H-2	25.8	C-1, C-2, C-5
4	1.25<br>1.73	m<br>m	H-5	21.4	C-2, C-6
5	1.45<br>1.56	m<br>m	H-4, H-6	24.9	C-3
6	3.03<br>3.36	ddd,13.4,13.2, 3.0<br>d, 13.4	H-5	44.9	C-2, C-4, C-5, C-8
7	-	-	-		-
8	-	-	-	167.1	-
9	-	-	-	175.8	-
10	-	-	-	99.3	-
11	2.28	m	H-12	35.2	C-9, 11-CH_3,C-12
11-CH_3	0.84	d, 6.7	H-11, H-12	15.5	C-10, C-11, C-12
12	1.55	m	H-11	26.2	-
13	1.54	m	H-14	31.1	-
14	4.08	m	H-13, H-15	68.8	-
15	1.62<br>1.94	m<br>m	H-14, H-16	41.3	C-14, 17-CH_3
16	4.20	dd, 11.6, 2.2	H-15	74.3	C-14, C-15, C-17, 17-CH_3, C-18
17	-	-	-	140.0	
17-CH_3	1.76	s	-	11.9	C-16, C-17, C-18
18	5.95	d, 10.9	17-CH_3, H-19	124.4	C-16, 17-CH_3, C-19, C-20
19	6.24	dd, 14.5, 10.9	H-18, H-20	127.0	C-17, C-18, C-21
20	6.02	dd, 14.5, 10.7	H-19, H-21	133.0	C-18, C-21, C-22
21	5.92	dd, 14.8, 10.7	H-20, H-22	130.1	C-19, C-20, C-23
22	5.20	dd, 14.8, 9.9	H-21, H-23	140.4	C-20, 23-CH_3, C-24
23	2.06	m	H-22, H-24	39.6	-

23-CH_3	0.98	d, 6.5	H-23	22.2	C-22, C-23, C-24
24	1.97<br>2.07	m<br>m	H-23,<br>H-25	39.5	C-22, 23-CH_3, C-25
25	2.53	m	H-24,<br>25-CH_3	43.0	C-24, 25-CH_3, C-26
25-CH_3	1.02	d, 7.3	H-25	19.3	C-24, C-25, C-26
26	-	-		215.6	-
27	2.31	m	-	48.5	-
28	1.36<br>1.95	m<br>m	H-29	30.6	C-25, C-26
29	1.16<br>1.36	m<br>m	H-28,<br>H-30	29.0	-
30	4.54	m	H-29,<br>H-31	74.4	C-1
31	1.24<br>1.91	m<br>m	H-30,<br>H-32	29.0	-
32	1.71<br>1.96	m<br>m	H-31	31.7	C-30, C-28,

Supplementary Figure 9 | ^{1}H NMR spectrum ($CDCl_3$, 500 MHz) of compound 3.

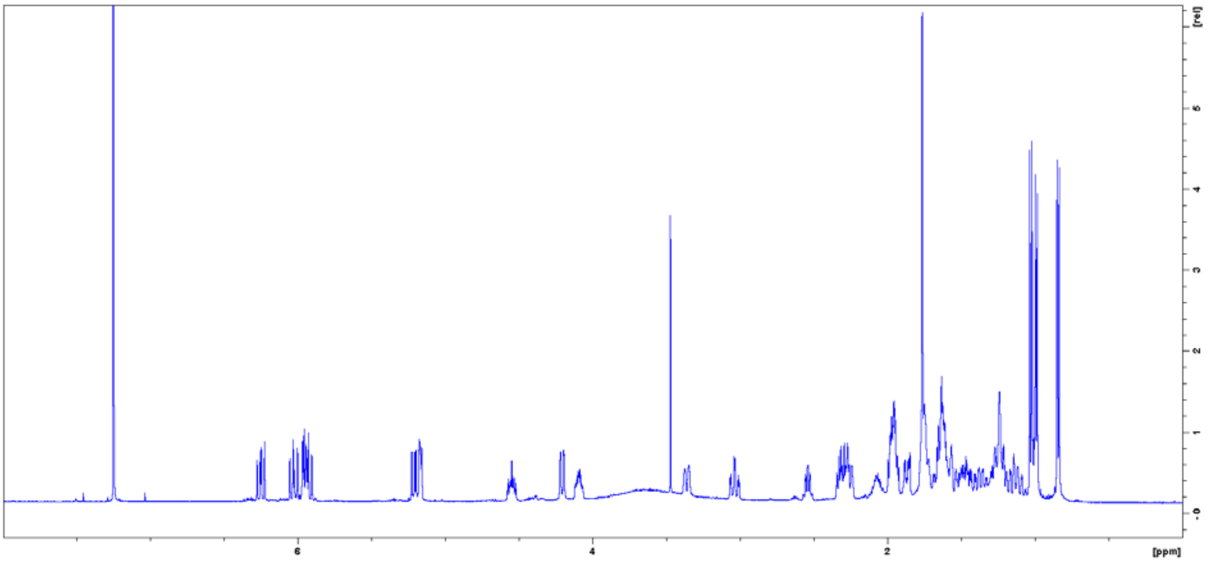

Supplementary Figure 10 | ^{13}C NMR spectrum ($CDCl_3$, 125 MHz) of compound 3.

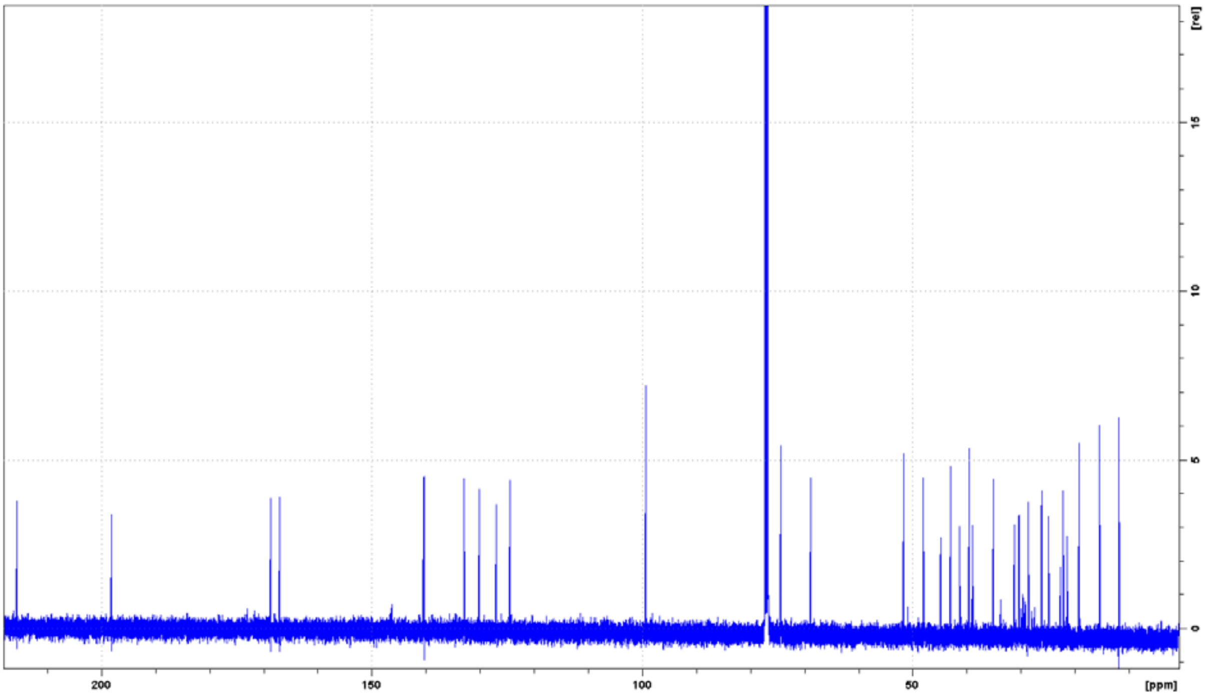

Supplementary Figure 11 | H,H-COSY spectrum ($CDCl_3$, 500 MHz) of compound 3.

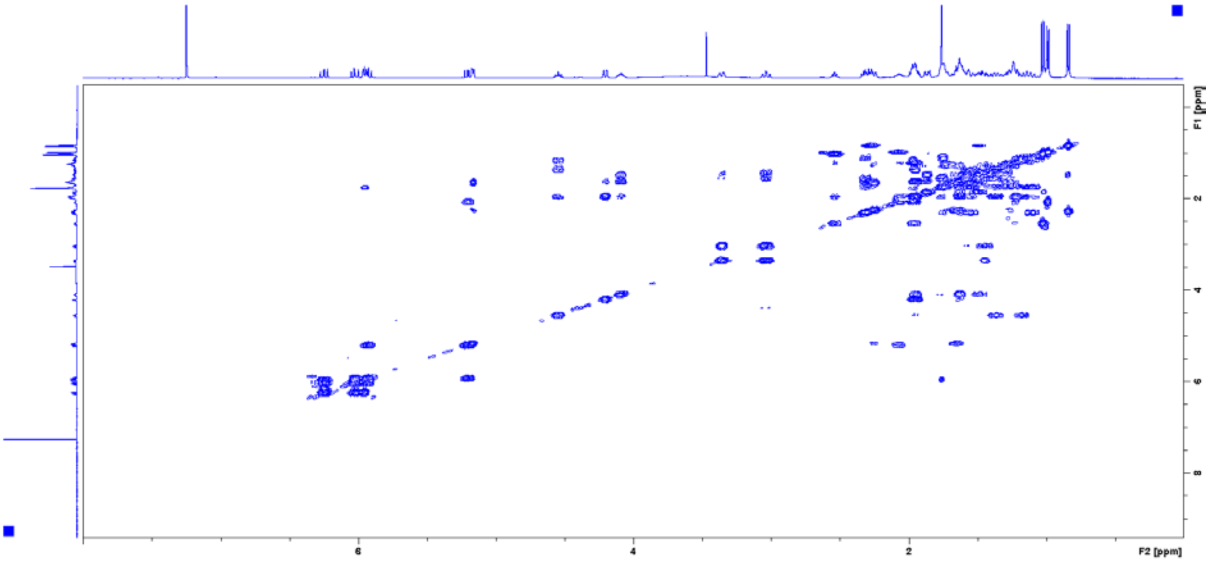

Supplementary Figure 12 | HSQC spectrum ($CDCl_3$, 500 MHz) of compound 3.

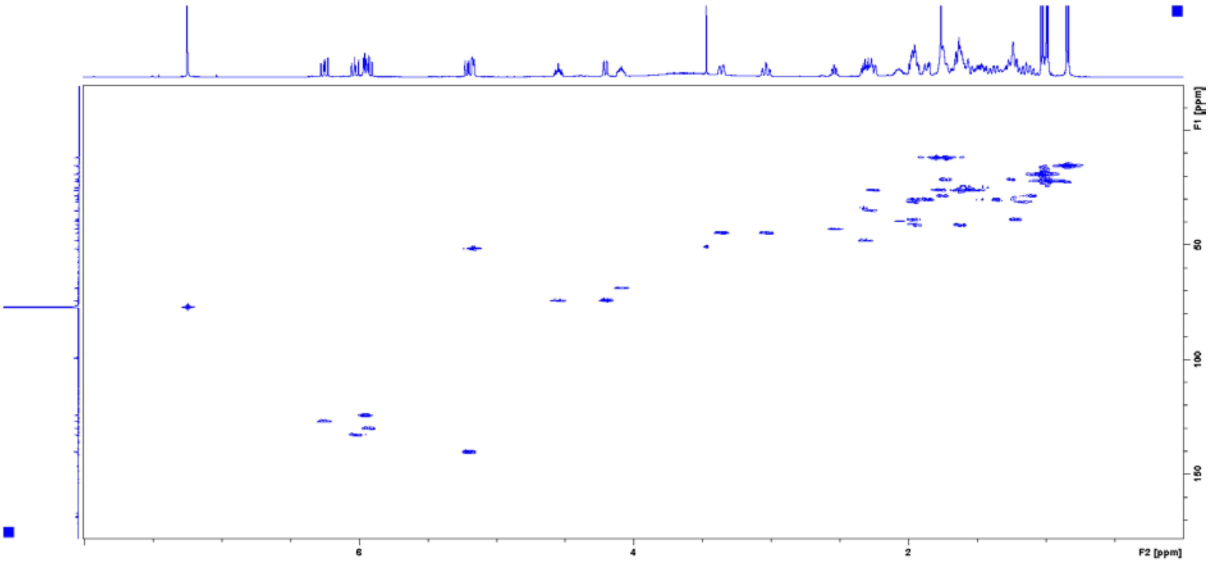

Supplementary Figure 13 | HMBC spectrum ($CDCl_3$, 500 MHz) of compound 3.

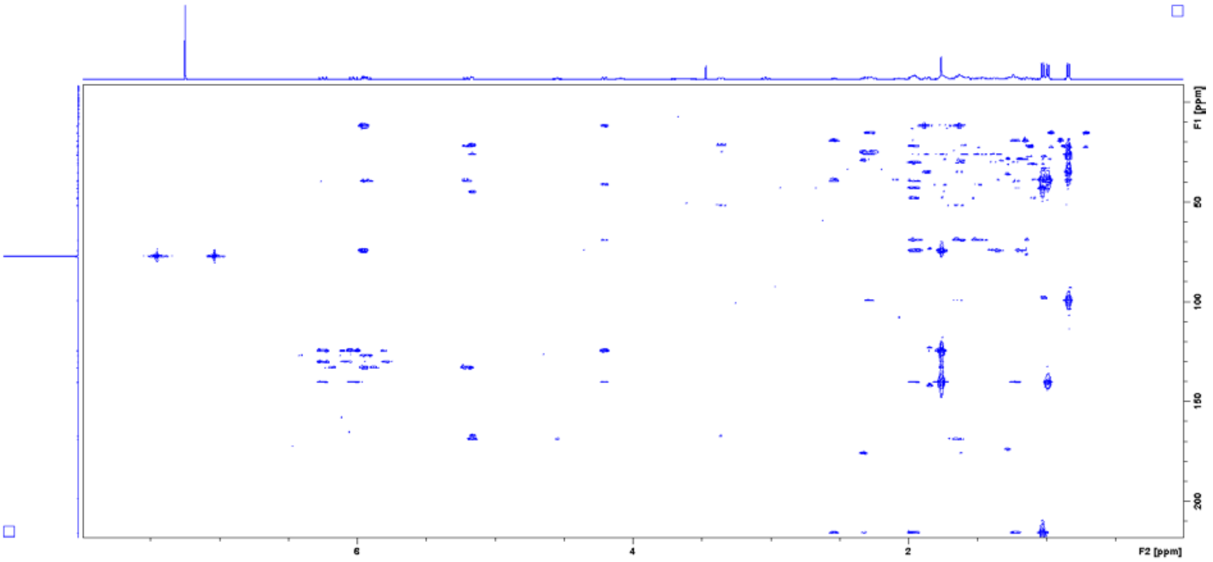

COMPOUND 4

The seed and production fermentations were performed as detailed in the methods section.

Strain: ISOM-4178

Feed: 4-*trans*-hydroxycyclohexanecarboxylic acid (hCHCA) (final concentration 2 mM)

Volume: 1 x 15 L fermentation

The fermentation broth at the end of the fermentation was separated by centrifugation and the cell paste processed as described for **2**.

The crude extract (9.8 g) was dissolved in 1:1 methanol/acetonitrile and C18 reverse-phase silica gel added. The solvent was removed under reduced pressure and the adsorbed silica was applied to a flash reverse-phase C18 silica column (70 mm x 55 mm diameter). The column was eluted with 6:4 water / acetonitrile (800 mL), 1:1 water / acetonitrile (200 mL), 4:6 water / acetonitrile (800 mL), and then 3:7 water / acetonitrile (200 L). Fractions containing **7** were combined and the solvent removed under reduced pressure to yield an enriched fraction (404 mg). This material was further purified by reverse-phase HPLC over a Phenomenex Xterra C18 column: 10 µm, 19 mm diameter x 250 mm. Mobile phase A = water, B = acetonitrile; gradient, t = 0 min, B = 30%; t = 30 min, B = 80%). Flow rate 20 mL min^{-1}. Fractions containing compound **4** were combined and the solvent evaporated under reduced pressure. This material was purified by size-exclusion chromatography over sephadex LH-20 resin (column dimensions 1000 mm x 30 mm diameter) and eluted with methanol. Fractions containing **4** were combined and the solvent evaporated under reduced pressure to yield **4** as a white amorphous solid (47 mg).

LC-MS analysis: RT = 12.7 min, *m/z* = 652.6 ([M+Na]$^+$) and 628.5 ([M-H]$^-$)

HRMS (*m/z*):, calcd for $C_{37}H_{59}NO_7Na$, 652.4189; found, 652.4190; Δ = 0.2 ppm.

Supplementary Figure 14 | Chemical structure and 2D NMR correlations for compound 4.

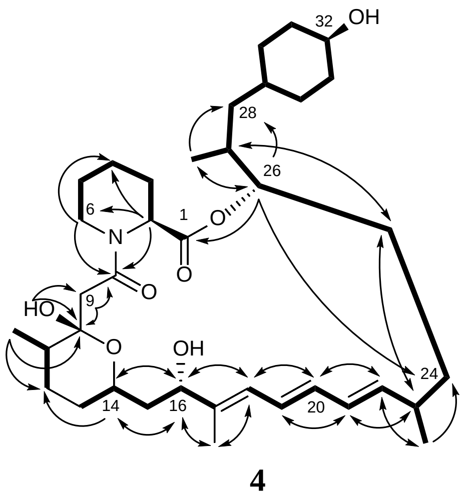

4

Supplementary Table 5 | NMR data for compound 4 in $CDCl_3$ at 500 MHz for ^1H and 125 MHz for ^{13}C.

Position	^1H-NMR			^{13}C-NMR	HMBC correlations
	δ ppm	Multiplicity, *J* in Hz	COSY	*δ* ppm	^1H to ^{13}C
1	-	-	-	170.6	-
2	5.17	d, 4.6, 1.7	H-3	52.6	C-1, C-3, C- C-4, C-6, C-8
3	1.72<br>2.30	m<br>m	H-2, H-4	26.4	-
4	1.22<br>1.72	m<br>m	H-3, H-5	20.8	-
5	1.47	m	H-4, H-6	29.0	-
6	3.19<br>3.68	ddd,13.1,12.9, 3.0<br>m	H-5	42.8	C-2, C-4, C-8
7	-	-	-		-
8	-	-	-	173.2	-
9	2.42<br>2.58	d, 16.1<br>d, 16.1	--	38.3	C-8, C-10
10	-	-	-	98.3	
10-OH	6.48	bs	-		C-10, C-9
11	1.33	m	11-CH_3, H-12	38.8	-
11-CH_3	0.85	d, 6.7	H-11	17.2	C-10, C-11, C-12
12	1.45<br>1.71	m<br>m	H-11	24.9	-
13	1.29<br>1.44	m<br>m	H-14	33.2	-
14	3.91	m	H-13, H-15	64.4	C-12, C-15, C-16
15	1.64<br>1.87	m<br>m	H-14, H-16	39.9	-
16	4.23	m	H-15	74.1	C-14, C-15, C-17, 17-CH_3, C-18
17	-	-	-	137.3	-
17-CH_3	1.59	s	-	13.3	C-16, C-17, C-18
18	6.11	d, 9.8	H-19	124.6	C-16, 17-CH_3, C-19, C-20
19	6.30	dd, 14.8, 10.6	H-18, H-20	127.5	C-18, C-21
20	6.22	dd, 14.8, 9.8	H-19, H-21	132.2	C-18, C-21, C-22
21	6.08	dd, 15.4, 9.9	H-20, H-22	129.1	C-19, C-20, C-23

22	5.59	dd, 15.4, 7.3	H-21, H-23	138.1	C-20, C-23, 23-CH_3, C-24,
23	2.02	m	H-22, 23-CH_3, H-24	35.5	C-21, C-22, 23-CH_3, C-25
23-CH_3	0.98	d, 6.8	H-23	22.9	C-22, C-23, C-24
24	1.50	m	H-23, H-25	28.1	-
25	1.50<br>1.63	m<br>m	H-24, H-26	27.9	C-23, C-22, C-26, C-27
26	4.72	ddd, 9.9, 3.1, 2.9	H-25, H-27	78.6	C-1, C-24, C-25, C-27-CH_3, C-28
27	1.78	m	H-26, 27-CH_3, H-28	31.4	C-25, C-26
27-CH_3	0.83	d, 6.7	H-27	15.6	C-26, C-27, C-28
28	0.91<br>1.07	m<br>m	H-27, H-29	40.2	-

Supplementary Figure 15 | ^1H NMR spectrum ($CDCl_3$, 500 MHz) of compound 4.

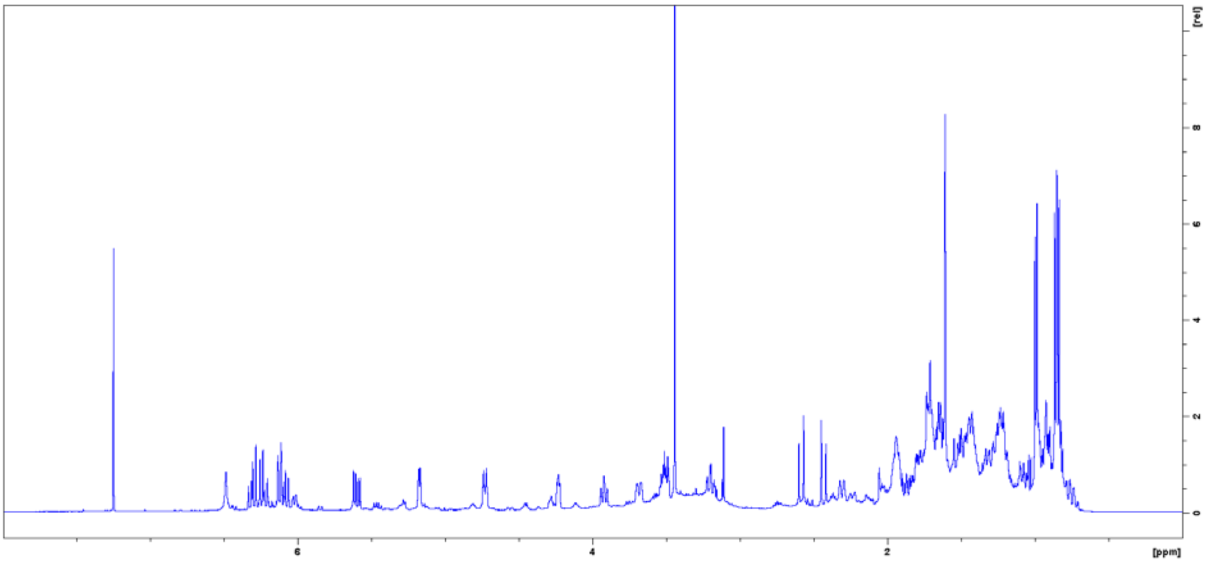

Supplementary Figure 16 | ^{13}C NMR spectrum ($CDCl_3$, 500 MHz) of compound 4.

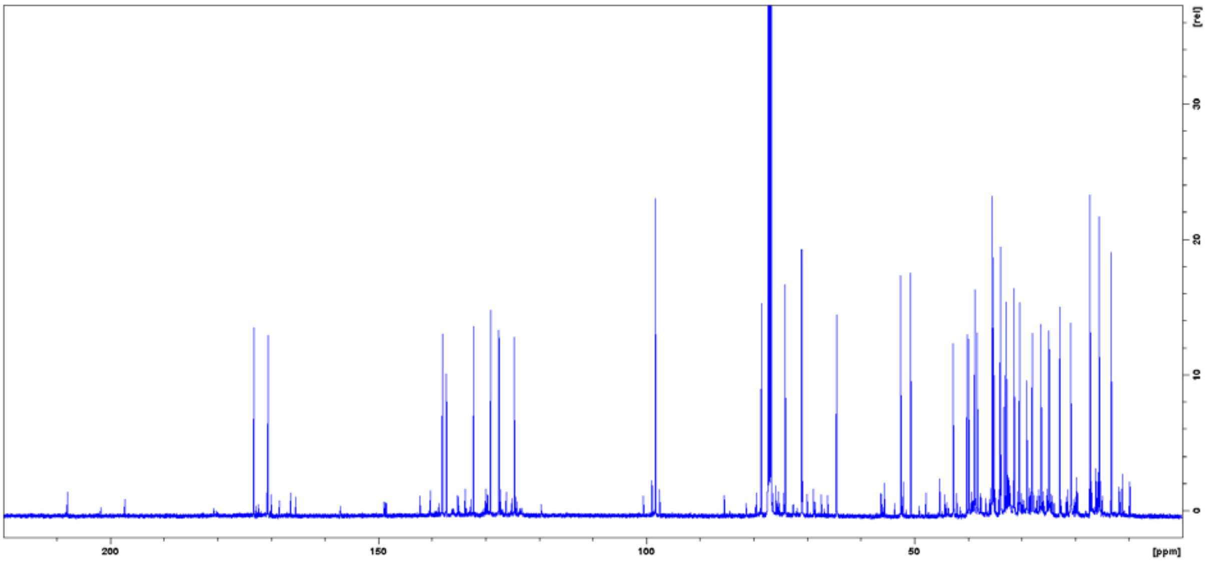

Supplementary Figure 17 | H,H-COSY NMR spectrum ($CDCl_3$, 500 MHz) of compound 4.

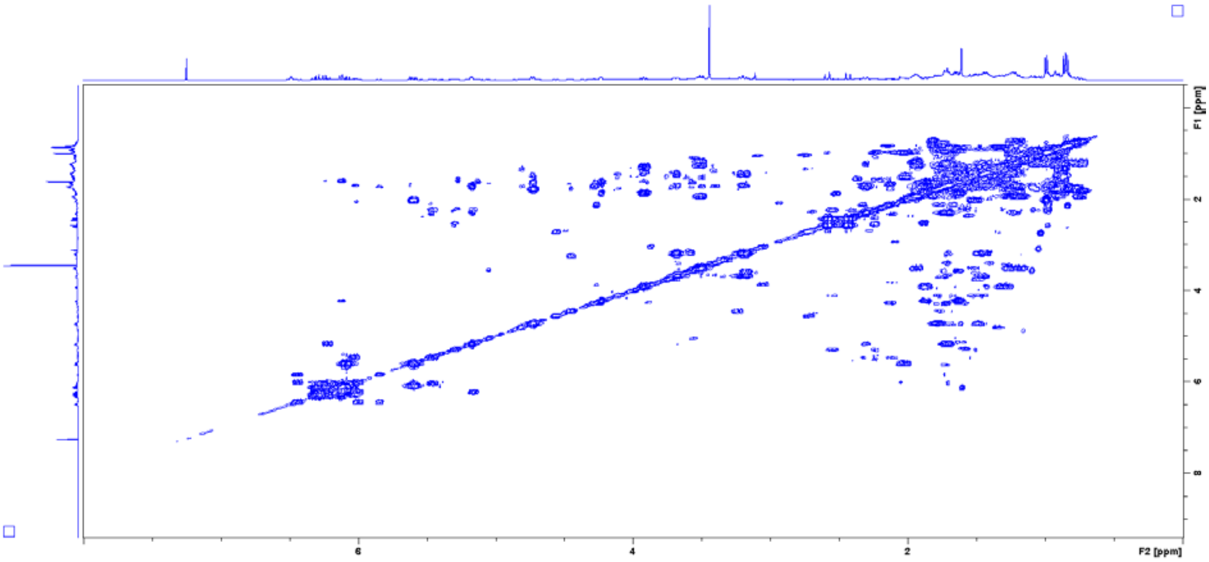

Supplementary Figure 18 | HSQC NMR spectrum ($CDCl_3$, 500 MHz) of compound 4.

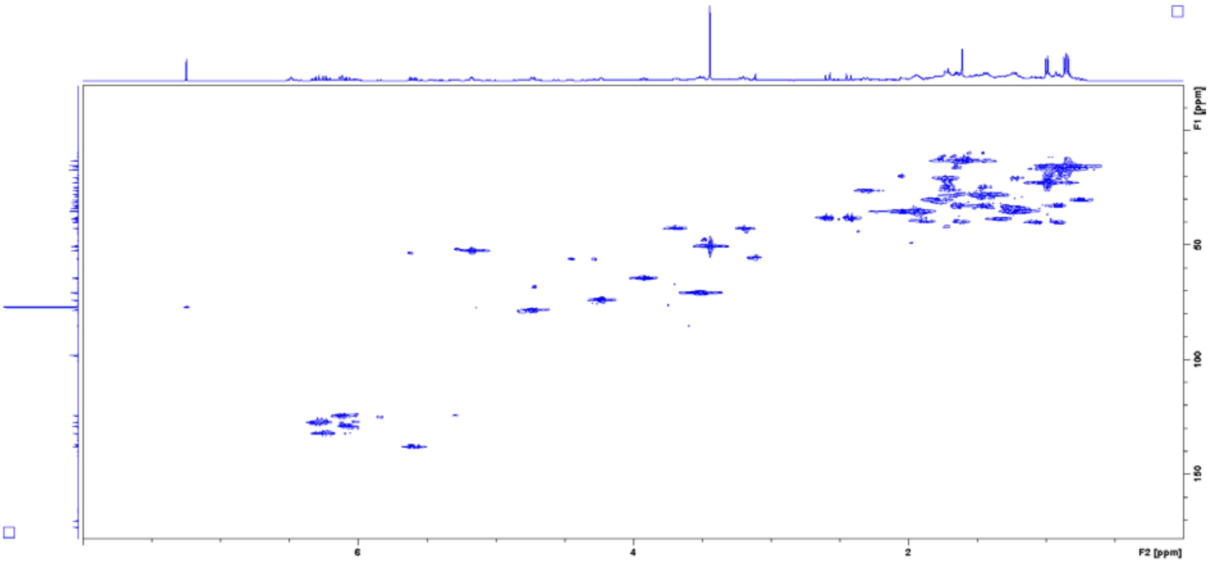

Supplementary Figure 19 | HMBC NMR spectrum ($CDCl_3$, 500 MHz) of compound 4.

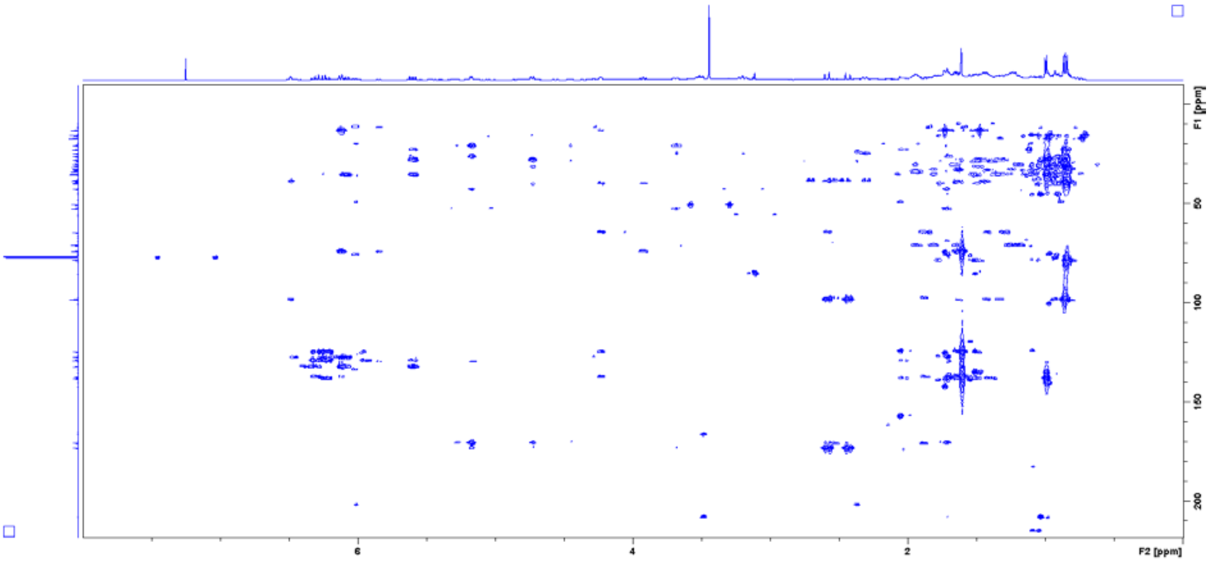

COMPOUND 5

The seed and production fermentations were performed as detailed in the Methods section.

Strain: ISOM-4185

Feed: 4-*trans*-hydroxycyclohexanecarboxylic acid (hCHCA) (final concentration 2 mM)

Volume: 1 x 15 L fermentation

The fermentation broth at the end of the fermentation was separated by centrifugation and the cell paste processed as described for **2**.

The crude extract (8 g) was dissolved in ethyl acetate and silica gel added (15 g). The solvent was removed under reduced pressure and the adsorbed silica was applied to a flash silica column (200 mm x 55 mm diameter). The column was eluted with 1:2 ethyl acetate / hexanes (900 mL), 4:5 ethyl acetate / hexanes (1.8 L), 1:1 ethyl acetate / hexanes (3 L), 3:2 ethyl acetate / hexanes (1 L), 2:1 ethyl acetate / hexanes (1.8 L) and then 100% ethyl acetate (400 mL). Fractions containing **5** were combined and reduced the solvent removed under reducer pressure. This material was dissolved in 2:3 acetonitrile/water and applied to a C18 SPE cartridge (20 g). This was eluted with 100 mL each of 2:3, acetonitrile/water, 1:1, 3:2, 7:3, 4:1. Fractions containing **5** were combined and the solvent removed under reduced pressure to yield an enriched fraction (150 mg). This material was further purified by reverse-phase HPLC over a Phenomenex Xterra C18 column: 10 µm, 19 mm diameter x 250 mm. Mobile phase: A = water, B = acetonitrile: t = 0 min, B = 50%; t = 22 min, B = 80%). Flow rate 20 mL min^{-1}. Fractions containing **5** were combined and the solvent evaporated under reduced pressure to yield **5** as a white amorphous solid (93 mg).

LC-MS data: RT = 14.3 min, *m/z* = 722.6 ($[M+Na]^+$) and 698.5 ($[M-H]^-$).

HRMS (*m/z*):, calcd for $C_{41}H_{65}NO_8Na$, 722.4608; found, 722.4601; Δ = -1.0 ppm.

Supplementary Figure 20 | Chemical structure and 2D NMR correlations for compound 5.

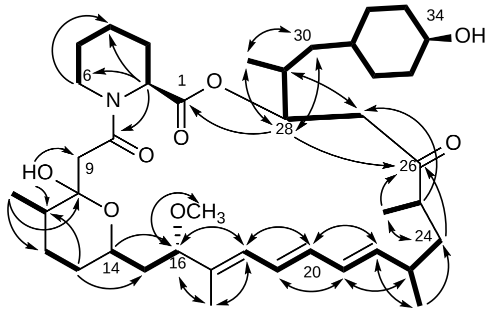

5

Supplementary Table 6 | NMR data for compound 5 in $CDCl_3$ at 500 MHz for ^1H and 125 MHz for ^{13}C.

Position	^1H-NMR *δ* ppm	^1H-NMR Multiplicity, *J* in Hz	^1H-NMR COSY	^{13}C-NMR *δ* ppm	HMBC correlations ^1H to ^{13}C
1	-	-	-	170.7	-
2	5.23	dd, 6.1, 2.0	H-3	52.2	C-1, C-3, C-4, C-6, C-8
3	1.75<br>2.26	m<br>m	H-2,<br>H-4	26.7	-
4	1.25<br>1.73	m<br>m	H-3,<br>H-5	20.6	-
5	1.51<br>1.73	m<br>m	H-4,<br>H-6	24.8	-
6	3.26<br>3.72	ddd, 13.2, 13.0, 3.0<br>m	H-5	42.7	C-2, C-4, C-5
7	-	-	-		-
8	-	-	-	173.4	-
9	2.55	s	-	38.0	-
10	-	-	-	98.2	-
10-OH	6.68	d, 1.2	-		C-9, C-11
11	1.13	m	11-CH_3	38.8	-
11-CH_3	0.86	d, 6.5	H-11	17.3	C-10, C-11, C-12
12	1.41<br>1.61	m<br>m	H-13	27.6	-
13	1.69<br>2.09	m<br>m	H-12	32.7	C-11, C-15
14	3.71	m	H-15	65.6	C-15, C-16
15	1.59<br>1.73	m<br>m	H-14,<br>H-16	38.7	C-13, C-14, C-16, C-17, 17-CH_3
16	3.68	dd, 10.9, 5.1	H-15	84.8	C-15, 16-OCH_3, 17-CH_3, C-18
16-OCH_3	3.10	s	-	55.6	C-16
17	-	-	-	133.3	-
17-CH_3	1.58	d, 0.7	-	10.1	C-16, C-17, C-18
18	6.06	dd, 9.8, 8.8	H-19	131.6	17-CH_3, C-16, C-19, C-20
19	6.31	dd, 14.9, 10.3	H-18,<br>H-20	126.3	C-18, C-20, C-21
20	6.38	dd, 14.9, 10.2	H-19,<br>H-21	134.3	C-18, C-21, C-22
21	6.11	dd, 15.0, 10.0	H-20,<br>H-22	130.0	C-19, C-20, C-23
22	5.45	dd, 15.0, 7.6	H-21,<br>H-23	138.9	C-20, C-23, 23-CH_3, C-24

23	2.23	m	H-22, 23-CH_3, H-24	36.0	C-21, C-22, 23-CH_3
23-CH_3	1.02	d, 4.4	H-23	21.3	C-22, C-23, C-24
24	1.27<br>1.58	m<br>m	H-23	40.4	C-23, C-25, 25-CH_3, C-26
25	2.44	m	25-CH_3	45.6	C-23, C-24, 25-CH_3, C-27
25-CH_3	1.00	d, 4.7	H-25	16.4	C-24, C-25, C-26
26	-	-	-	210.8	-
27	2.66<br>2.73	dd, 16.8, 5.4<br>dd, 16.8, 4.9	H-28	41.1	C-26, C-28, C-29
28	4.97	ddd, 5.3, 5.3, 5.3	H-27, H-29	75.6	C-1, C-26, C-27, C-29, C-30,
29	2.08	m	H-28, 29-CH_3, H-30	32.4	C-27, C-28, 29-CH_3, C-30, C-31
29-CH_3	0.84	d, 6.8	H-29	15.8	C-28, C-29, C-30
30	0.98	m	H-29, H-31	39.0	29-CH_3, C-32
31	1.27	m	H-30, H-32, H-36	33.9	-
32	0.95<br>1.47	m<br>m	H-31, H-33	32.6	-
33	1.24<br>1.92	m<br>m	H-32, H-34	35.5	C-31, C-32, C-34, C-35, C-36
34	3.50	m	H-33, H-35	71.0	-
35	1.17<br>1.94	m<br>m	H-34, H-36	35.2	-
36	0.78<br>1.78	m<br>m	H-31, H-35	30.5	-

Supplementary Figure 21 | ^{1}H NMR spectrum ($CDCl_3$, 500 MHz) of compound 5.

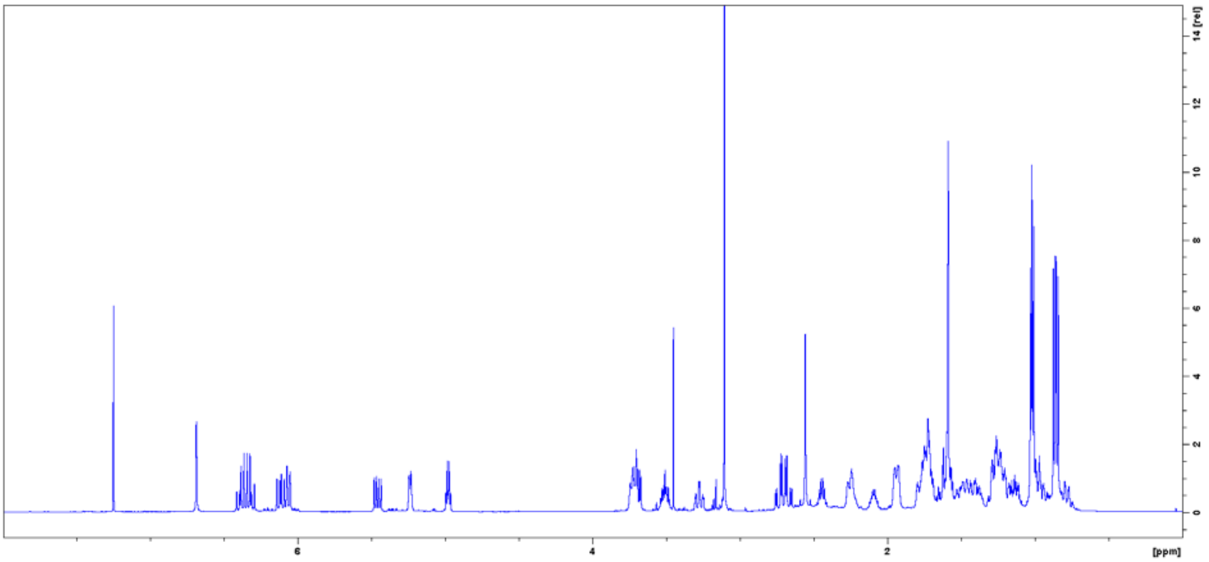

Supplementary Figure 22 | ^{13}C NMR spectrum ($CDCl_3$, 500 MHz) of compound 5.

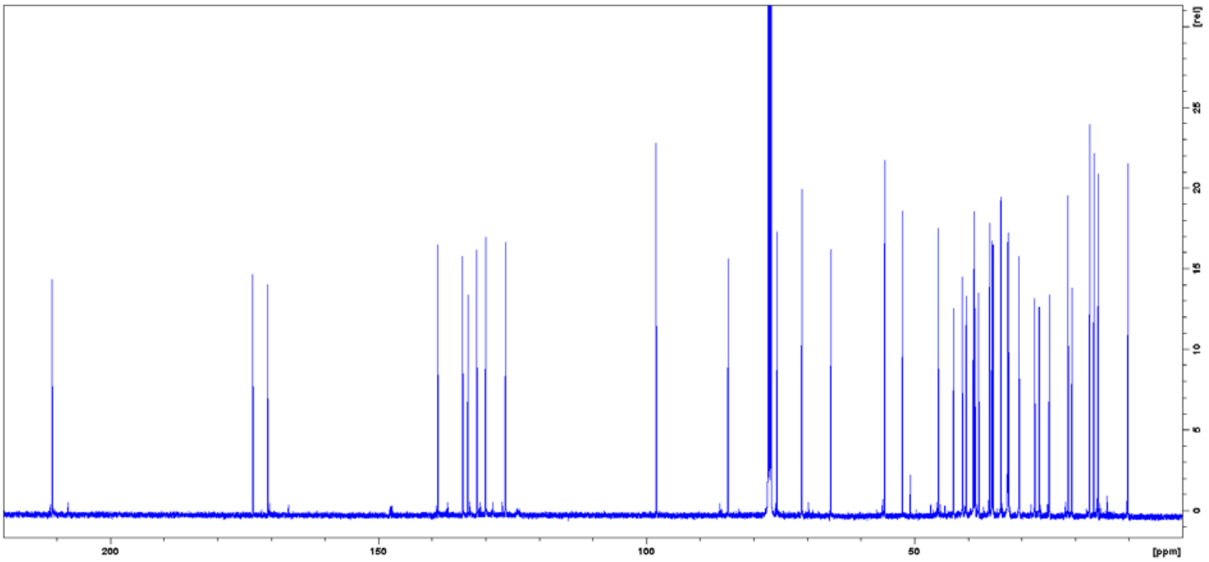

Supplementary Figure 23 | H,H-COSY NMR spectrum ($CDCl_3$, 500 MHz) of compound 5.

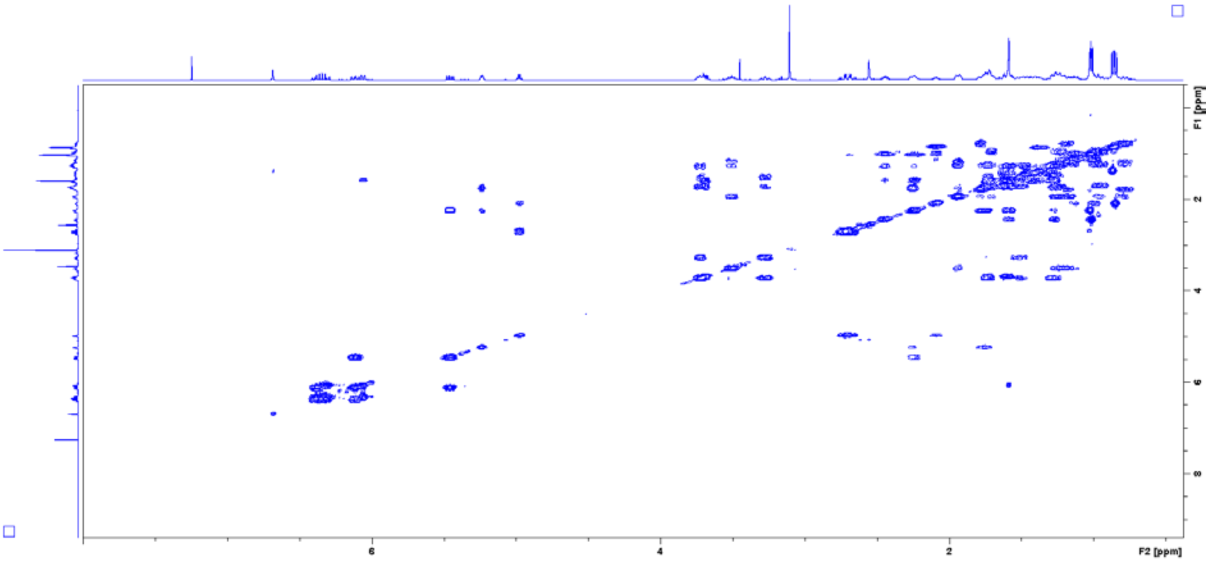

Supplementary Figure 24 | HSQC NMR spectrum ($CDCl_3$, 500 MHz) of compound 5.

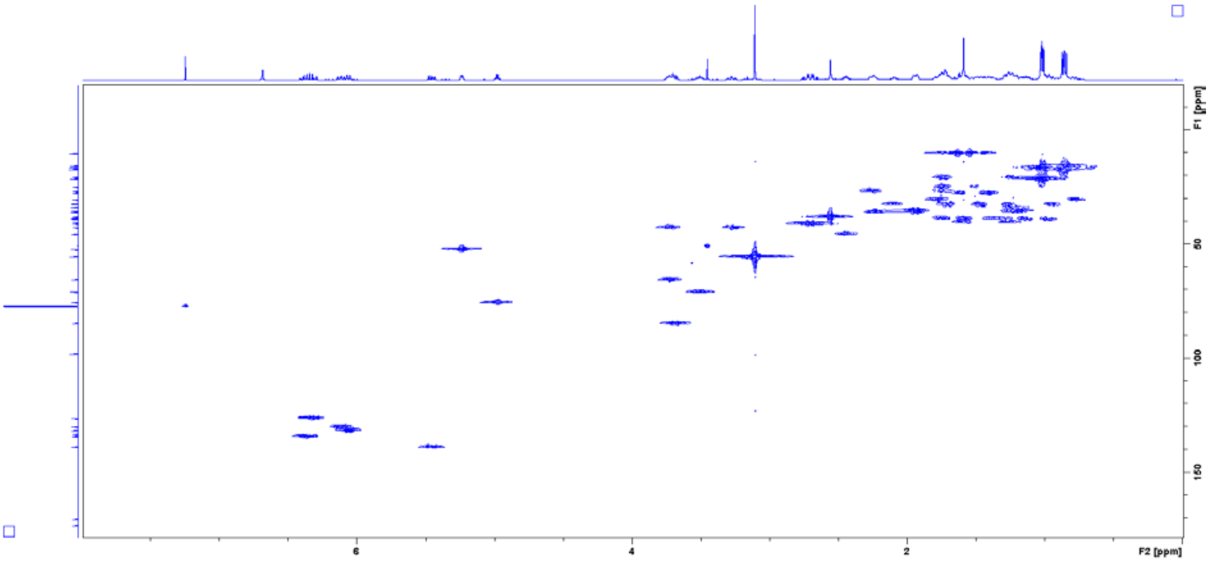

Supplementary Figure 25 | HMBC NMR spectrum ($CDCl_3$, 500 MHz) of compound 5.

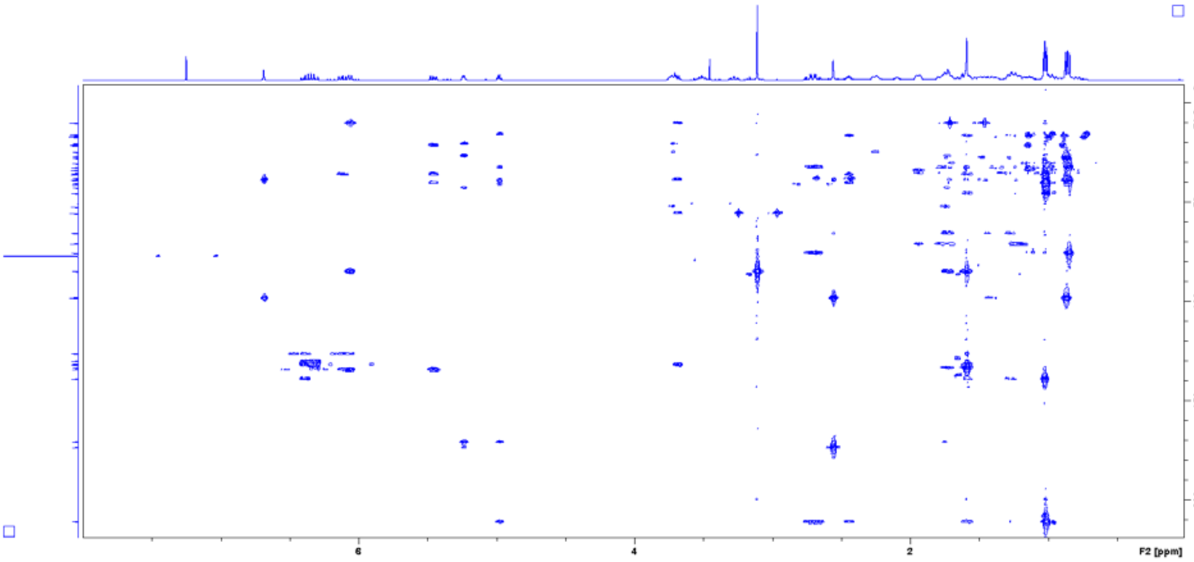

COMPOUND 6

The seed and production fermentations were performed as detailed in the methods section.

Strain: ISOM-4144

Feed: 4-*trans*-hydroxycyclohexanecarboxylic acid (hCHCA) (final concentration 2 mM)

Volume: 80 x 7 mL Falcon tubes; 560 mL.

The individual falcons were combined and the cell mass separated by centrifugation. Only the cell mass extracted using the method describe for **2**.

The crude extract (1.2 g) was dissolved in ethyl acetate and silica gel added (10 g). The solvent was removed under reduced pressure and the adsorbed silica was applied to a flash silica column (180 mm x 55 mm diameter). The column was eluted with 4:5 ethyl acetate / hexanes (1.3 L) then 1:1 ethyl acetate / hexanes (3.4 L). Fractions containing **6** were combined and the solvent removed under reduced pressure to yield an enriched fraction (65 mg). This was further purified by reverse-phase HPLC over a Phenomenex Xterra C18 column: 10 µm, 19 mm diameter x 250 mm. Mobile phase: A = water, B = acetonitrile; t = 0 min, B = 50%; t = 22 min, B = 80%). Flow rate 20 mL min^{-1}. Fractions containing **6** were combined and the solvent evaporated under reduced pressure to yield **6** as a white amorphous solid (47 mg).

LC-MS: RT = 15.5 min, *m/z* = 780.3 ($[M+Na]^+$) and 756.2 ($[M-H]^-$).

HRMS (*m/z*):, calcd for $C_{43}H_{67}NO_{10}Na$, 780.4663; found, 780.4648; Δ = -1.9 ppm.

Supplementary Figure 26 | Chemical structure and 2D NMR correlations for compound 6.

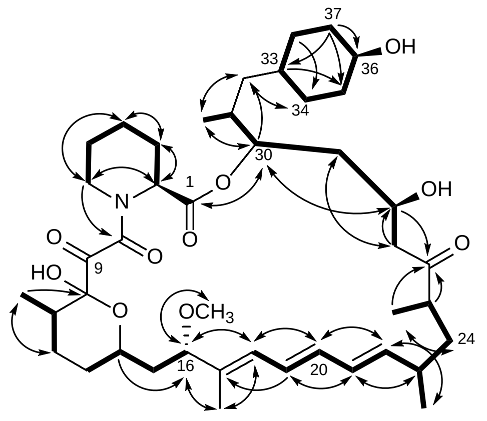

6

Supplementary Table 7 | NMR data for compound 6 in $CDCl_3$ at 500 MHz for ^{1}H and 125 MHz for ^{13}C.

Position	^{1}H-NMR			^{13}C-NMR	HMBC correlations
	δ ppm	Multiplicity, *J* in Hz	COSY	*δ* ppm	^{1}H to ^{13}C
1	-	-	-	169.6	-
2	5.26	dd, 5.4, 1.2	H-3	52.2	C-1, C-3, C-4, C-6, C-8
3	1.75<br>2.35	m<br>m	H-2,<br>H-4	27.0	C-2, C-4, C-5
4	1.33<br>1.76	m<br>m	H-3,<br>H-5	21.1	C-3, C-6
5	1.55<br>1.68	m<br>m	H-3,<br>H-6	25.1	-
6	3.30<br>3.71	ddd, 13.4, 13.2, 3.1<br>m	H-5	44.1	C-2, C-4, C-8
7	-	-	-	-	-
8	-	-	-	165.9	-
9	-	-	-	194.4	-
10	-	-	-	98.8	-
11	2.00	m	11-CH_3, H-12	33.8*	C-10, 11-CH_3, C-12,
11-CH_3	0.86	d, 6.7	H-11	16.1	C-10, C-12
12	1.55	m	H-11	27.2	-
13	1.54	m	H-14	31.1	-
14	3.73	m	H-13,<br>H-15	66.9	C-16
15	1.53<br>1.82	m<br>m	H-14,<br>H-16	38.4	C-14, C-16, C-17
16	3.70	dd, 9.7, 6.0	H-15	84.1	C15, 16-OCH_3, 17-CH_3, C-18

16-OCH_3	3.09	s	-	55.8	C-16
17	-	-	-	135.3	-
17-CH_3	1.61	s		10.1	C-16, C-17, C-18
18	6.04	m	H-19	129.7	C-16, 17-CH_3, C-20
19	6.32	dd, 14.9, 10.5	H-18, H-20	126.6	C-17, C-21
20	6.25	dd, 14.9, 10.0	H-19, H-21	133.4	C-18, C-22
21	6.07	m	H-20, H-22	130.1	C-19, C-23
22	5.39	dd, 15.0, 8.3	H-21, H-23	139.3	C-20, C-23, 23-CH_3, C-40
23	2.20	m	H-22, 23-CH_3, H-24	36.5	C-21, C-22, 23-CH3, C-24,
23-CH_3	0.99	d, 3.76	H-23	21.4	C-22, C-23, C-24, C-25
24	1.30<br>1.65	m<br>m	H-23, H-25	40.2	C-23, C-25
25	2.48	q, 6.7	H-24, 25-CH_3	46.0	C-24, 25-CH_3, C-26
25-CH_3	1.01	d, 3.94	H-25	16.8	C-24, C-25, C-26
26				215.1	
27	2.53	d, 6.0	H-28	47.2	C-26, C-28, C-29
28	4.12	m	H-27, H-29	65.7	C-26, C-29, C-30
29	1.64<br>1.73	m<br>m	H-28, H-30	36.1	C-28, C-
30	5.00	m	H-29, H-31	77.5	C-1, C-28, C-29, 31-CH_3, C-32, C-33
31	2.02	m	H-30, 31-CH_3	34.3	31-CH_3
31-CH_3	0.87	d, 6.7	H-31	15.2	C-30, C-32, C-33
32	1.52	m		39.4	C-34
33	1.25	m	H-34, H-38	33.8*	-
34	0.83<br>1.76	m<br>m	H-33, H-35	30.6	C-32
35	1.18<br>1.95	m<br>m	H-36, H-34	35.3	C-33, C-36
36	3.51	m	H-35, H-37	71.0	C-35, C-37
37	1.26<br>1.92	m<br>m	H-36, H-38	35.6	C-33, C-35, C-36
38	0.95<br>1.71	m<br>m	H-37, H-33	32.4	C-34

Supplementary Figure 27 | ^{1}H NMR spectrum ($CDCl_3$, 500 MHz) of compound 6.

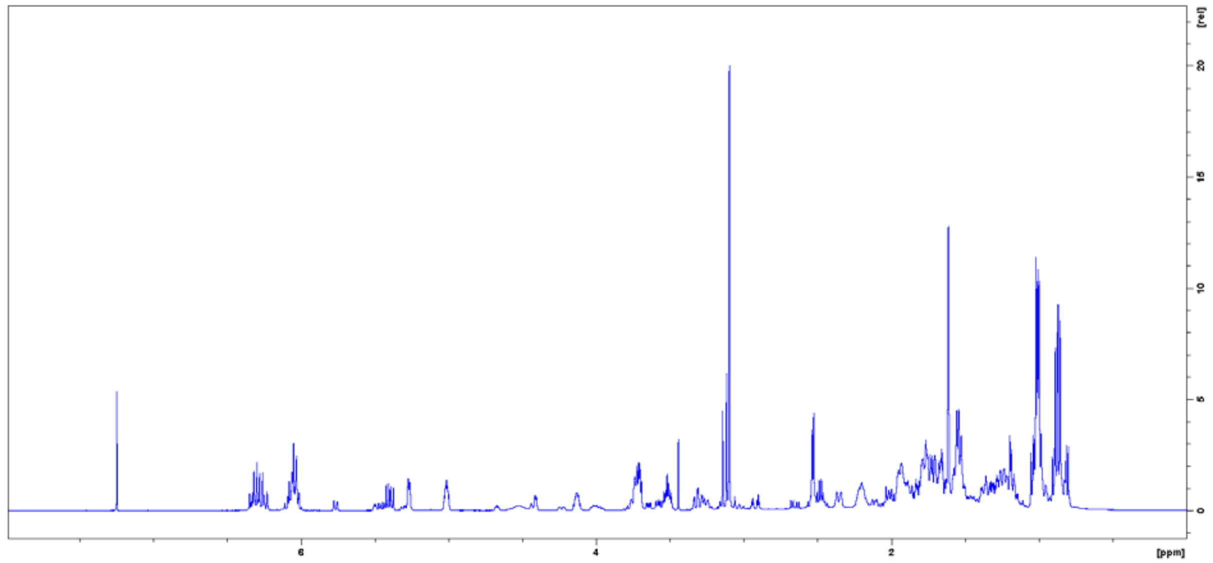

Supplementary Figure 28 | ^{13}C NMR spectrum ($CDCl_3$, 500 MHz) of compound 6.

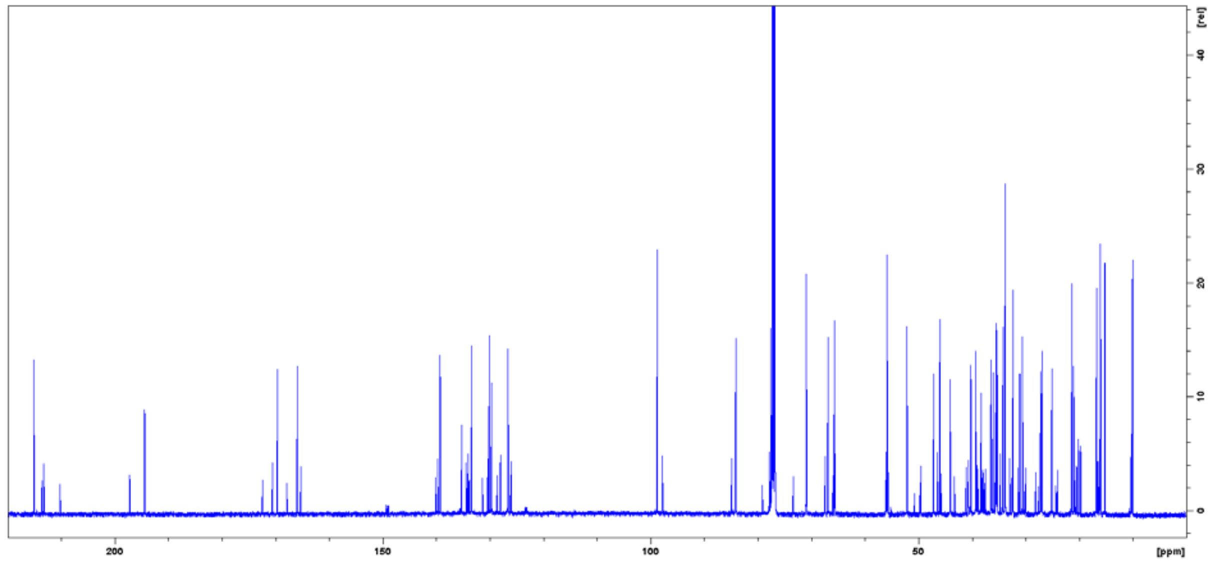

Supplementary Figure 29 | H,H-COSY NMR spectrum ($CDCl_3$, 500 MHz) of compound 6.

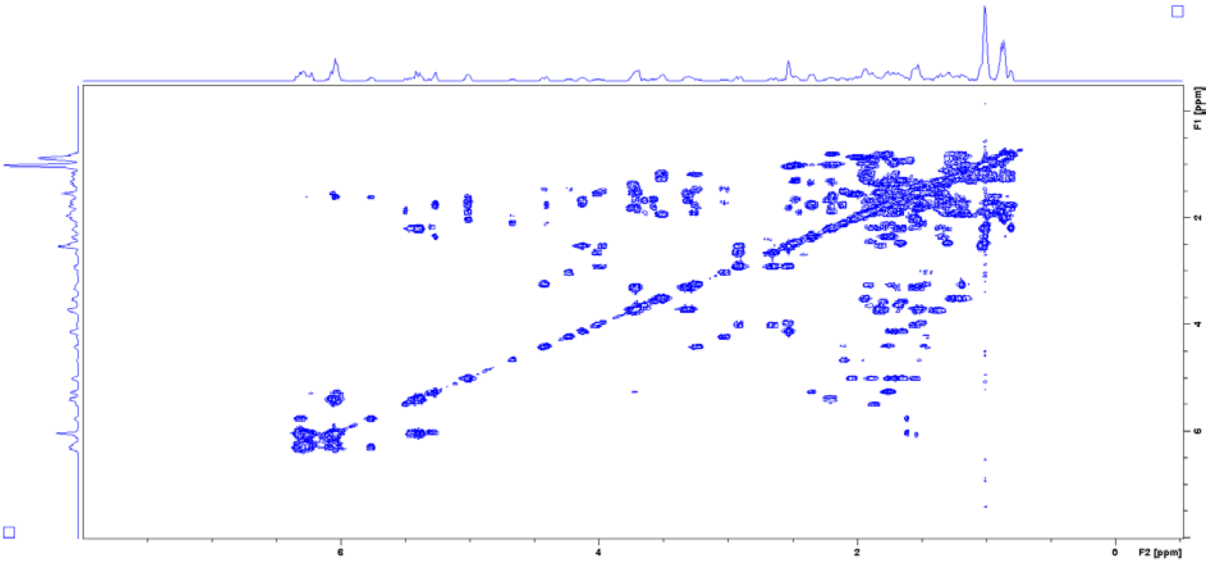

Supplementary Figure 30 | HSQC NMR spectrum ($CDCl_3$, 500 MHz) of compound 6.

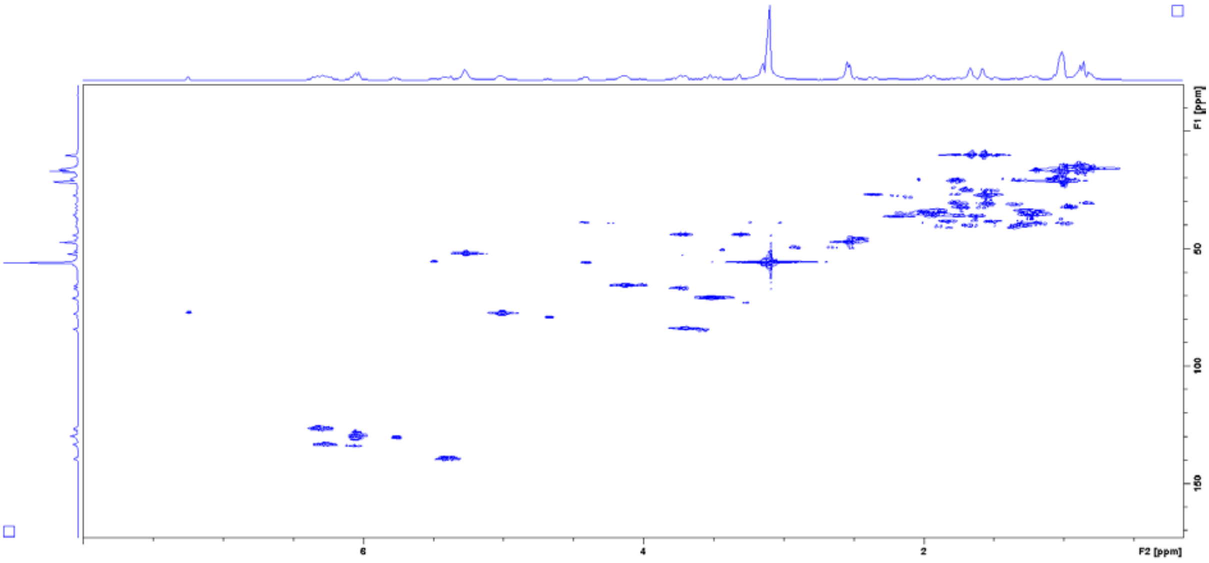

Supplementary Figure 31 | HMBC NMR spectrum ($CDCl_3$, 500 MHz) of compound 6.

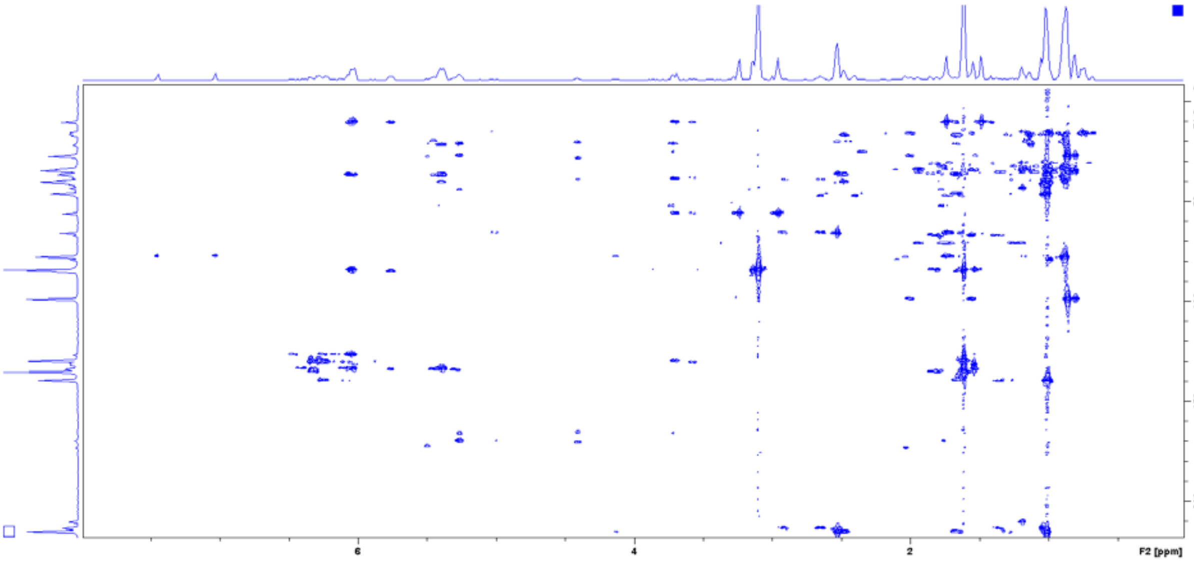

COMPOUND 7

The seed and production fermentations were performed as detailed in the methods section.

Strain: ISOM-4141

Feed: 4-*trans*-hydroxycyclohexanecarboxylic acid (hCHCA) (final concentration 2 mM)

Volume: 1 x 15 L fermentation

The fermentation broth at the end of the fermentation was separated by centrifugation and the cell paste processed as described for **2**.

The crude extract was dissolved in ethyl acetate and silica gel added (7.5 g). The solvent was removed under reduced pressure and the adsorbed silica was applied to a flash silica column (200 mm x 55 mm diameter). The column was eluted with 4:5 ethyl acetate / hexanes (1.8 L), 1:1 ethyl acetate / hexanes (3.0 L), 3:2 ethyl acetate / hexanes (1.5 L) and then 2:1 ethyl acetate / hexanes (0.9 L). Fractions containing **7** were combined and the solvent removed under reduced pressure (44 mg). This material was further purified by reverse-phase HPLC over a Phenomenex Xterra C18 column, 10 µm, 19 mm diameter x 250 mm. Mobile phase: A = water, B = acetonitrile; t = 0 min, B = 50%; t = 22 min, B = 80%). Flow rate at 20 mL min^{-1}. Fractions containing **7** were combined and the solvent evaporated under reduced pressure to yield the **7** as a white amorphous solid (32.6 mg).

LC-MS: RT = 14.8 min, *m/z* = 820.7 ($[M+Na]^+$) and 796.4 ($[M-H]^-$).

HRMS (*m/z*):, calcd for $C_{46}H_{71}NO_{10}Na$, 820.4976; found, 820.4964; Δ = -1.5 ppm.

Supplementary Figure 32 | Chemical structure and 2D NMR correlations for compound 7.

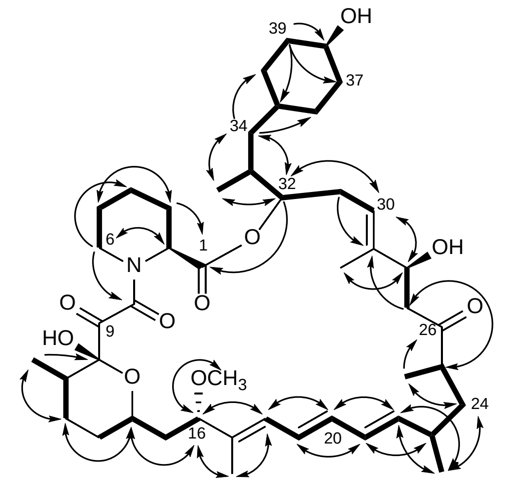

7

Supplementary Table 8 | NMR data for compound 7 in $CDCl_3$ at 500 MHz for ^{1}H and 125 MHz for ^{13}C.

Position	^{1}H-NMR			^{13}C-NMR	HMBC correlations
	δ ppm	Multiplicity, *J* in Hz	COSY	*δ* ppm	^{1}H to ^{13}C
1	-	-	-	169.2	-
2	5.20	dd, 5.5, 5.2	H-3	51.3	C-1, C-3, C-4, C-6, C-8
3	1.68<br>2.27	m<br>m	H-2, H-4	26.7	C-5
4	1.39<br>1.75	m<br>m	H-3, H-5	21.0	-
5	1.46<br>1.65	m<br>m	H-5, H-6	25.1	C-3
6	3.27<br>3.47	m<br>m	H-5	44.4	C-2, C-4, C-5, C-8
7	-	-	-	-	-
8	-	-	-	166.6	-
9	-	-	-	195.1	-
10	-	-	-	98.8	-
11	2.08	m	11-CH_3,<br>H-12	34.4	C-10, 11-CH_3, C-12
11-CH_3	0.88	-	H-11	16.0	C-10, C-11, C-12
12	1.58	m	H-11	26.9	C-14
13	1.30	m	H-14	30.7	C-14
14	3.95	m	H-13,<br>H-15	67.5	C-12, C-15, C-16
15	1.47	m	H-14,	39.5	C-13, C-14, C-16, C-17

	1.89	m	H-16		
16	3.58	dd, 8.7, 5.8	H-15	83.8	C-14, C-15, 16-OCH_3, 17-CH_3, C-18
16-OCH_3	3.09	s	-	55.9	C-16
17	-	-	-	136.8	-
17-CH_3	1.63	s	-	10.1	C-16, C-17, C-18
18	5.88	dd, 19.9, 11.0	H-19	128.5	C-16, 17-CH_3, C-19, C-20
19	6.32	dd, 14.7 11.0	H-18,<br>H-20	126.9	C-20, C-21,
20	6.16	dd, 14.7, 10.6	H-19,<br>H-21	133.0	C-18, C-19, C-22
21	6.05	dd, 15.0, 10.6	H-20,<br>H-22	129.7	C-19, C-23
22	5.43	dd, 15.0, 8.8	H-21,<br>H- 23	140.3	C-20, C-23, 23-CH_3, C-24
23	2.16	m	23-CH_3,<br>H-24	37.8	-
23-CH_3	1.00	d, 9.6	H-23	21.3	C-22, C-23, C-24
24	1.33<br>1.81	m<br>m	H-23,<br>H-25	40.5	C-22, C-23, C-25, 25-CH_3
25	2.52	m	H-24,<br>25-CH_3	46.2	C-23, C-24, 25-CH_3, C-27
25-CH_3	1.02	d, 7.0		17.9	C-24, C-25, C-26
26	-	-	-	215.2	-
27	2.43<br>2.57	dd, 16.8, 1.9<br>dd, 16.8, 9.8	H-28	47.3	C-25, C-28, C-29
28	4.35	m	H-27	71.8	29-CH_3, C-27, C-30, C-29
29	-	-	-	139.6	-
29-CH_3	1.56	s	H-30	13.1	C-28, C-29, C-30
30	5.36	dd, 7.1, 6.9	29-CH_3,<br>H-31	118.8	C-28, 29-CH_3, C-31, C-32, C-29
31	2.18<br>2.28	m<br>m	H-30,<br>H-32	28.9	C-29, C-30, C-32, C-33
32	4.73	m	H-31,<br>H-33	79.3	C-1, C-30, C-33, 33-CH_3, C-34,
33	1.79	m	H-32,<br>33-CH_3,<br>H-34	32.6	-
33-CH_3	0.88	dd 6.5, 4.4	H-33	16.4	C-32, C-33, C-34
34	1.07<br>1.22	dd, 9.6, 9.2<br>m	H-33,<br>H-35,<br>H-40	38.1	33-CH_3, C-36, C-40
35	1.21	m	H-34,<br>H-36	33.9	-
36	0.81	m	H-35,	30.3	-

	1.75	m	H-37		
37	1.19<br>1.96	m<br>m	H-36,<br>H-38	35.4	-
38	3.53	m	H-37,<br>H-39	71.0	-
39	1.26<br>1.93	m<br>m	H-38,<br>H-40	35.6	C-35, C-37, C-38
40	0.98<br>1.68	m<br>m	H-34,<br>H-39	32.6	-

Supplementary Figure 33 | ^{1}H NMR spectrum ($CDCl_3$, 500 MHz) of compound 7.

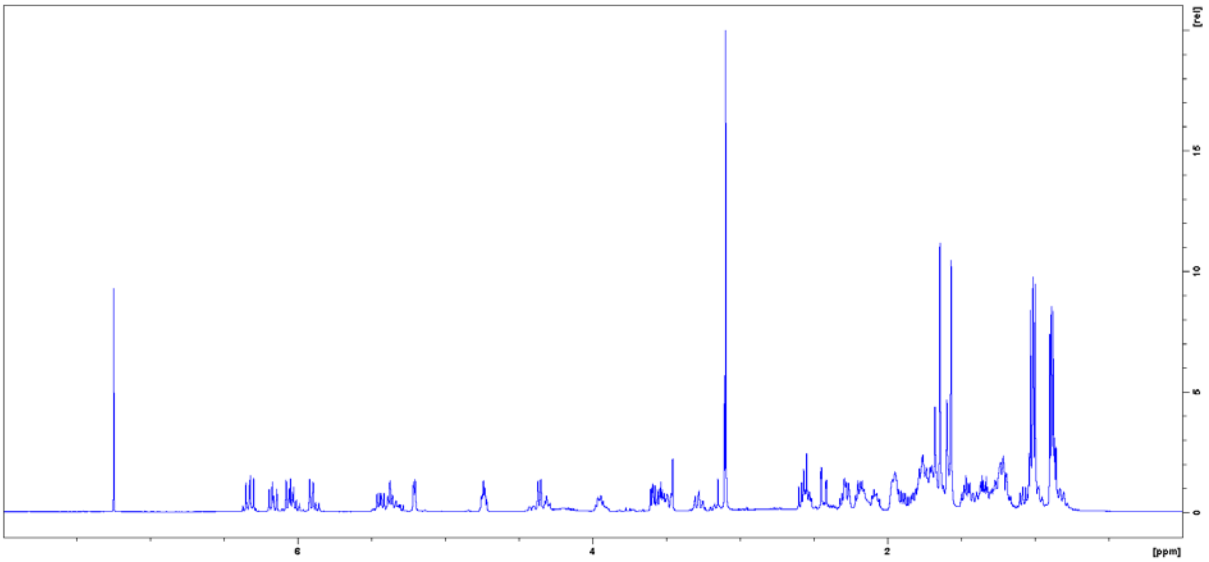

Supplementary Figure 34 | ^{13}C NMR spectrum ($CDCl_3$, 500 MHz) of compound 7.

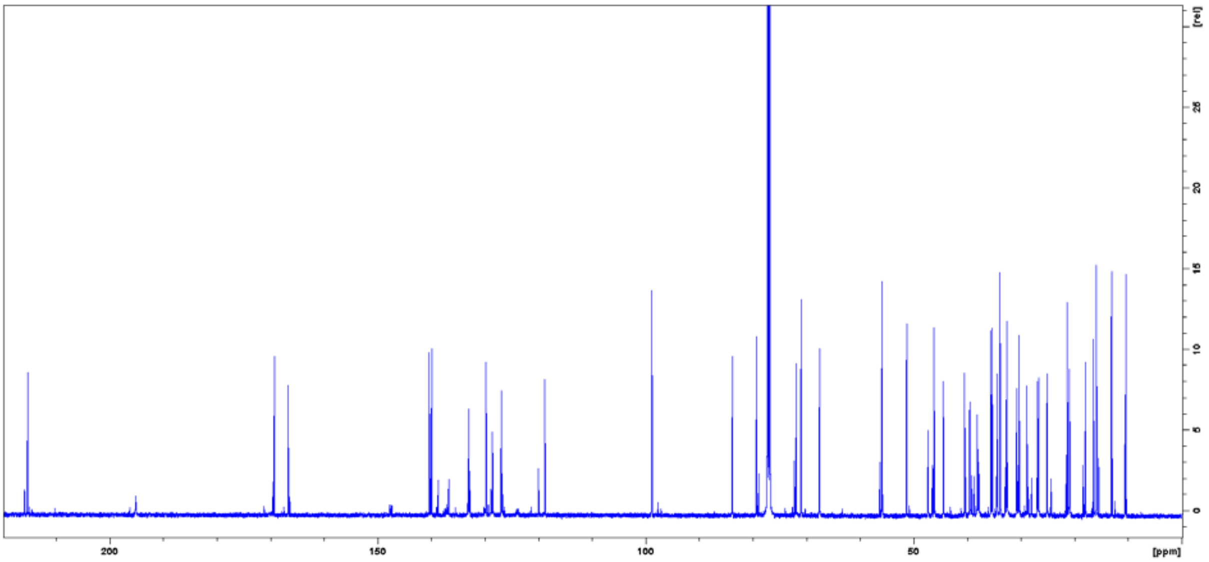

Supplementary Figure 35 | H,H-COSY NMR spectrum ($CDCl_3$, 500 MHz) of compound 7.

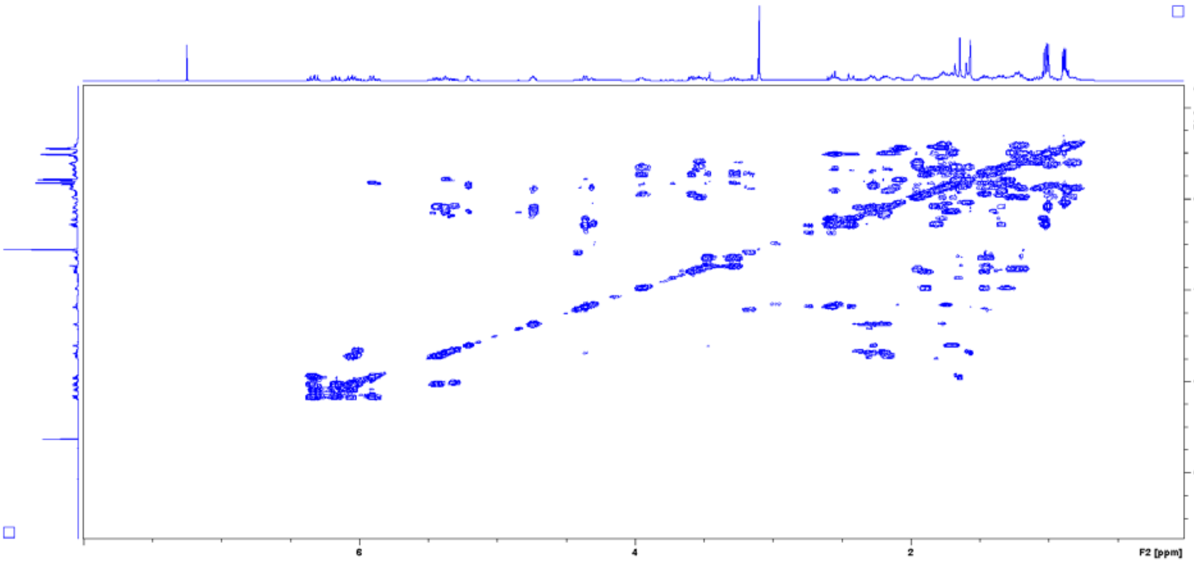

Supplementary Figure 36 | HSQC NMR spectrum ($CDCl_3$, 500 MHz) of compound 7.

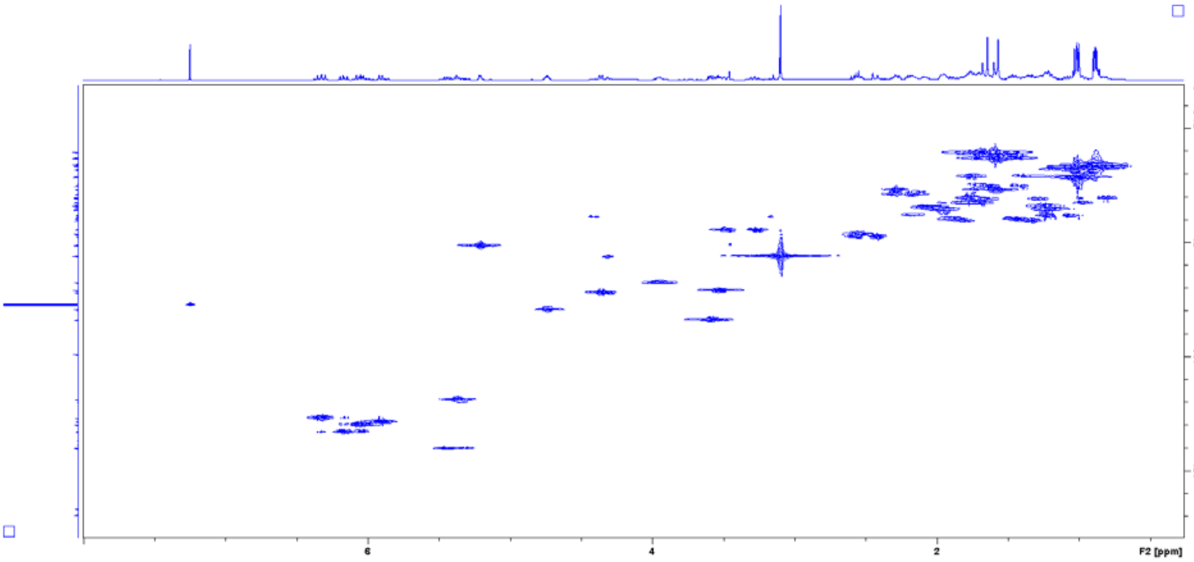

Supplementary Figure 37 | HMBC NMR spectrum ($CDCl_3$, 500 MHz) of compound 7.

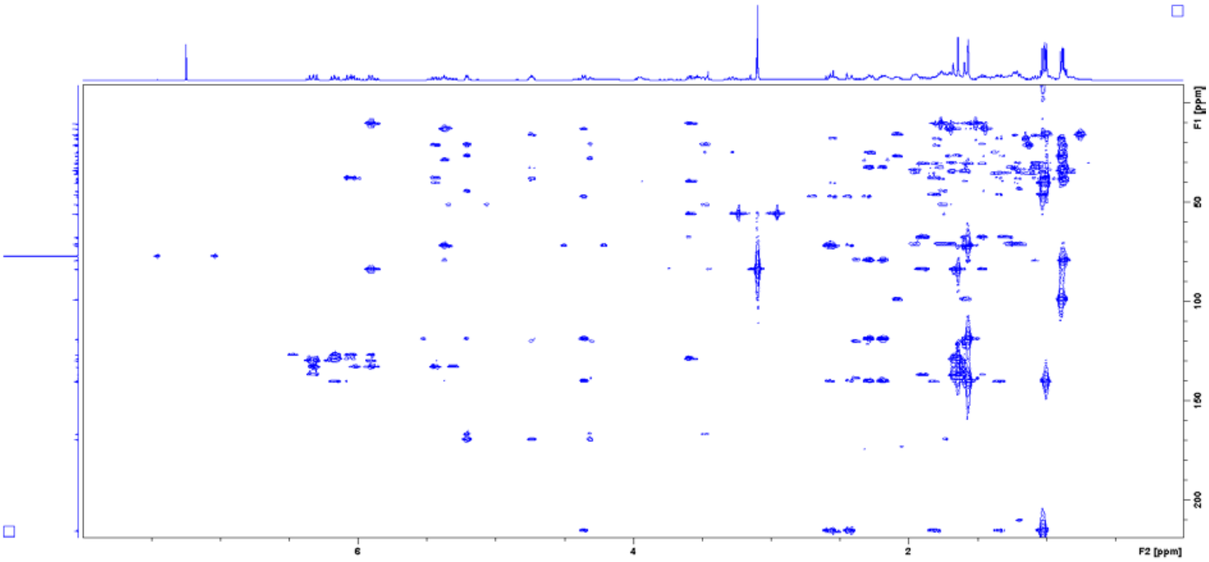

COMPOUND 8

The seed and production fermentations were performed as detailed in the methods section.

Strain: ISOM-4309

Feed: 4-*trans*-hydroxycyclohexanecarboxylic acid (hCHCA) (final concentration 2 mM)

Volume: 1 x 15 L fermentation

The fermentation broth at the end of the fermentation was separated by centrifugation and the cell paste processed as described for **2**.

The crude extract (5.3 g) was dissolved in methanol and silica added. The solvent was removed under reduced pressure and the adsorbed silica added to a flash silica column (20 cm x 5 cm diameter) and eluted with the 50:50 ethyl acetate/hexanes and then 60:40, 70:30, 80:20, 90:10 mixtures followed by 100% ethyl acetate. Fractions containing **8** were combined and dried under reduced pressure to yield an enriched extract (50 mg). This was loaded onto a second silica column (10 cm x 2 cm) and eluted with 45% ethyl acetate / 55% hexanes. Fractions containing **8** were combined and the solvent removed (32 mg). This was further purified by reverse-phase HPLC over a Phenomenex Xterra C18 column, 10 µm, 19 mm diameter x 250 mm. Mobile phase: A = water, B = acetonitrile; t = 0 min, B = 50%, t = 30 min, B = 100%). Flow rate at 20 mL min^{-1}. Fractions containing **8** were combined, concentrated, and subjected to a second round of reverse-phase HPLC over a Phenomenex Gemini NX C18 column, 10 µm, 21 mm diameter x 150 mm. Mobile phase: A = water, B = acetonitrile; gradient t = 0 min, B = 50%, t = 30 min, B = 100%). Flow rate 20 mL min^{-1}. Fractions containing **8** were combined and the solvent removed under reduced pressure to yield **8** as a white amorphous solid (14.5 mg).

LC-MS analysis: RT = 15.2 min, *m/z* = 920.6 ($[M+Na]^+$) and 896.5 ($[M-H]^-$).

HRMS (*m/z*):, calcd for $C_{51}H_{79}NO_{12}Na$, 920.5500; found, 920.5479; Δ = -2.3 ppm.

Supplementary Figure 38 | Chemical structure and 2D NMR correlations for compound 8.

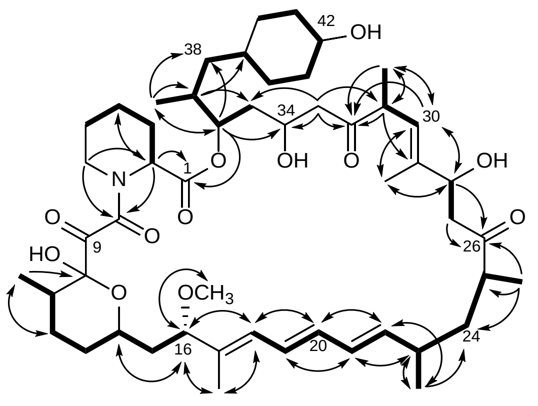

8

Supplementary Table 9 | NMR data for compound 8 in $CDCl_3$ at 500 MHz for ^{1}H and 125 MHz for ^{13}C.

Position	^{1}H-NMR δ ppm	^{1}H-NMR Multiplicity, *J* in Hz	^{1}H-NMR COSY	^{13}C-NMR δ ppm	HMBC correlations ^{1}H to ^{13}C
1	-	-	-	170.5, 170.3	-
2	5.20	d, 5.5	H-3	56.7, 52.1	C-1, C-3, C-4, C-6, C-8
3	1.72<br>2.28	m<br>m	H-2,<br>H-4	27.0, 26.9	C-1, C-3, C-4, C-5, C-8
4	1.44<br>1.77	m<br>m	H-3,<br>H-5	21.1, 20.9	-
5	1.52<br>1.67	m<br>m	H-4	25.2, 24.4	-
6	3.35<br>3.53	m<br>m	-	44.7, 40.0	C-1, C-8
7	-	-	-	-	-
8	-	-	-	167.7, 166.5	-
9	-	-	-	198.8, 195.6	-
10	-	-	-	99.5, 99.0	-
11	2.12	m	-	34.1, 34.1	-
11-CH_3	0.76	d, 7.0	H-11,<br>H-12	16.2, 15.9	C-10, C-11, C-12
12	1.73<br>1.52	m<br>m	H-11,<br>H-13	28.0, 27.4	-
13	1.73<br>1.23	m<br>m	H-12,<br>H-14	30.7, 30.7	-
14	4.06	m	H-13,<br>H-15	67.7, 67.7	-

15	1.66<br>1.72	m<br>m	H-14,<br>H-16	39.0, 38.5	-
16	3.66	m	H-15	84.7, 84.3	C-14, C-15, 16-OCH_3, 17-CH_3, C-18
16-OCH_3	3.09	s	-	55.5, 55.4	C-16
17	-	-	-	136.6, 135.6	-
17-CH_3	1.62	s	-	10.0, 9.9	-
18	6.00	d, 11.0	H-19	130.3, 129.4	C-16, 17-CH_3, C-19, C-20
19	6.42	dd, 15.0, 11.0	H-18,<br>H-20	126.9, 126.8	C-17, C-20, C-21
20	6.28	dd, 15.0, 11.0	H-19,<br>H-21	134.1, 133.8	C-18, C-19, C-22
21	6.16	dd, 15.0, 11.0	H-20,<br>H-22	130.7, 130.6	C-19, C-20, C-22, C-23
22	5.38	dd, 15.0, 8.5	H-21,<br>H-23	140.8, 140.6	C-20, C-23, 23-CH_3, C-24
23	2.23<br>2.44	m<br>m	H-22,<br>23-CH_3,<br>H-24	35.6, 35.6	C-21, 23-CH_3
23-CH_3	0.99	d, 7.0	H-23	21.9, 21.6	C-22, C-23, C-24
24	1.80<br>1.49	m<br>m	H-23,<br>H-25	39.3, 39.3	-
25	2.45	m	H-24,<br>25-CH_3	45.2, 45.0	-
25-CH_3	0.99	d, 7.0	H-25	15.4, 15.2	C-24, C-25, C-26
26	-	-	-	214.4, 214.0	-
27	2.63<br>2.46	m<br>m	H-28	47.4, 47.3	C-26
28	4.28<br>4.33	m<br>m	H-27	73.7, 73.4	C-26, C-27, C-29, 29-CH3, C-30
29	-	-		140.2, 139.3	-
29-CH_3	1.66	s	H-30	11.8, 11.6	C-28, C-29, C-30
30	5.27	dd, 7.5, 7.5	29-CH_3,<br>H-31	125.3, 124.8	C-28, 29-CH_3, C-31, 31-CH_3, C-32
31	3.40<br>3.49	dd, 17.0, 6.0<br>m	H-30,<br>31-CH_3	46.8, 46.0	C-29, C-30, 31-CH_3, C-32
31-CH_3	1.02<br>1.13	m<br>m	H-31	16.2, 16.5	C-30, C-31, C-32
32	-	-	-	212.0, 211.3	-
33	2.50<br>2.58	m<br>m	-	47.5, 47.7	C-31, C-32, C-34, C-35
34	2.94	dd, 17.5, 3.5	-	65.6, 65.1	-
35	1.68<br>1.73	m<br>m	H-36	37.7, 37.5	-
36	5.01	m	H-35,	77.4, 76.9	C-1, C-34, 37-CH_3, C-38

37	1.81	m	H-37<br>37-CH_3,<br>H-38	31.7, 31.1	C-35, C-39
37-CH_3	0.87	d, 6.7	H-37	16.1, 16.2	C-36, C-37, C-38
38	1.12<br>1.18	m<br>m	H-37	38.7, 38.5	C-39
39	1.16	-	H-38,<br>H-40,<br>H-44	36.4, 35.3	-
40	0.82<br>1.70	-	H-39,<br>H-41	32.7, 32.7	-
41	1.14<br>1.87	m<br>m	H-40,<br>H-42	35.8, 35.8	-
42	3.38	m	H-41,<br>H-43	70.5, 70.4	-
43	1.19<br>1.83	m<br>m	H-42,<br>H-44	35.8, 35.8	-
44	0.89<br>1.66	m<br>m	H-39,<br>H-43	32.7, 32.7	-

Supplementary Figure 39 | ^{1}H NMR spectrum ($CDCl_3$, 500 MHz) of compound 8.

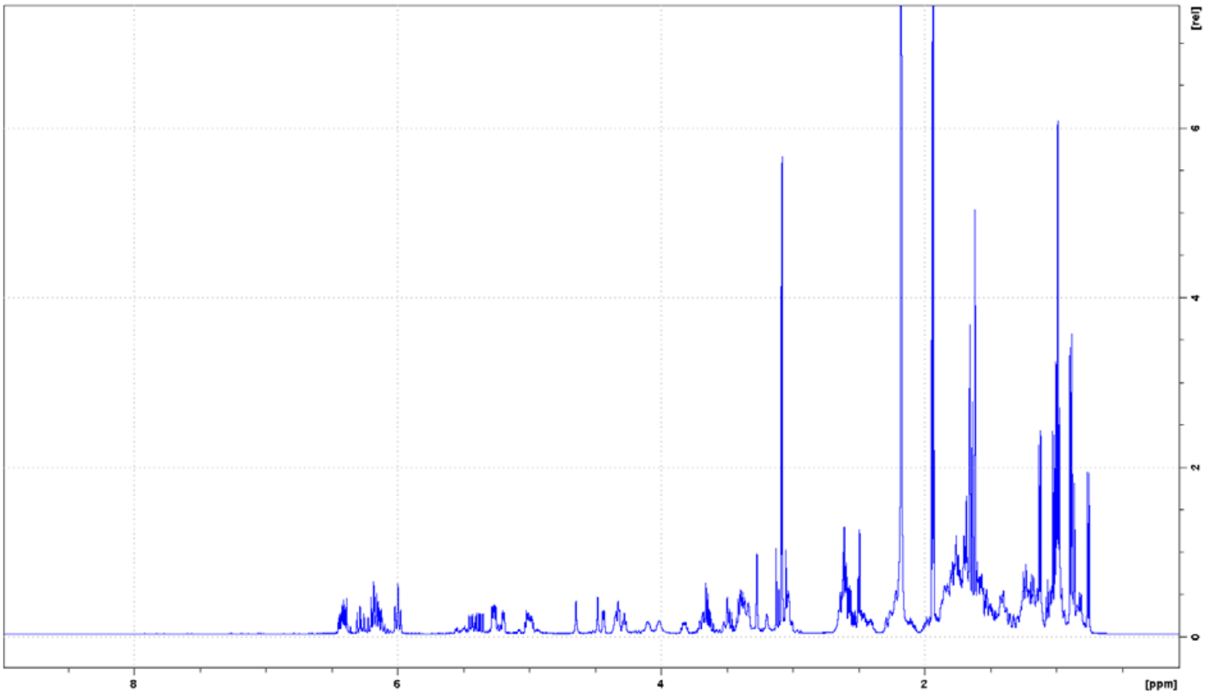

Supplementary Figure 40 | ^{13}C NMR spectrum ($CDCl_3$, 500 MHz) of compound 8.

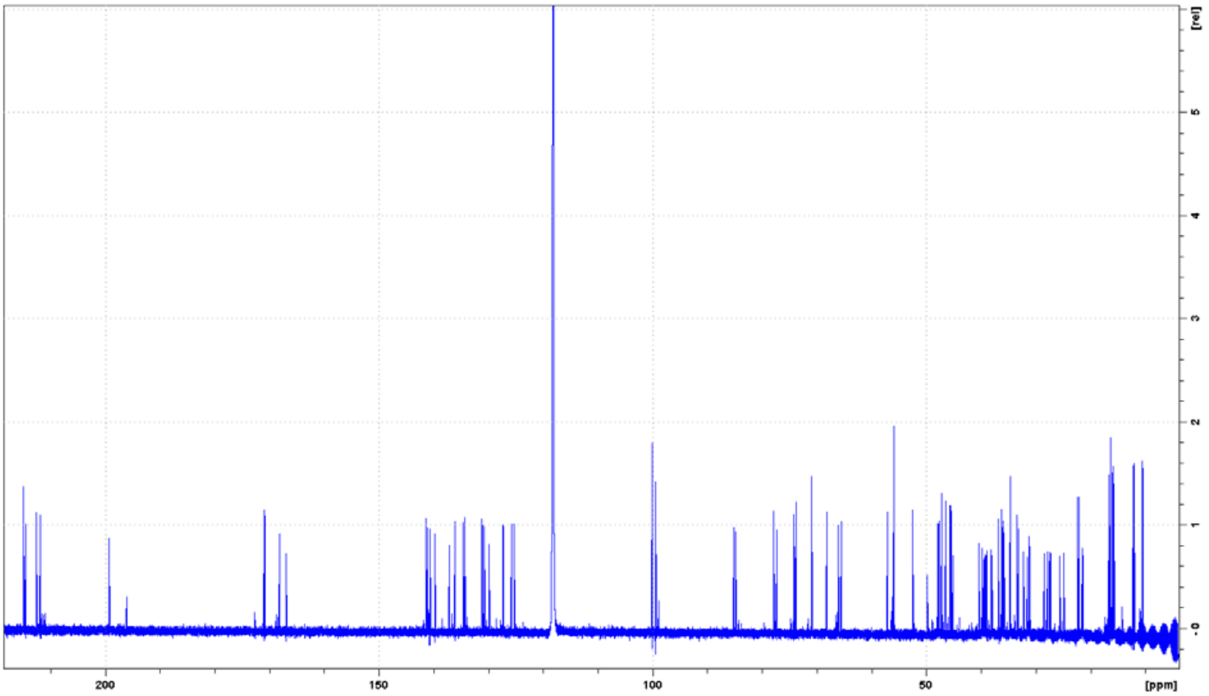

Supplementary Figure 41 | H,H-COSY NMR spectrum ($CDCl_3$, 500 MHz) of compound 8.

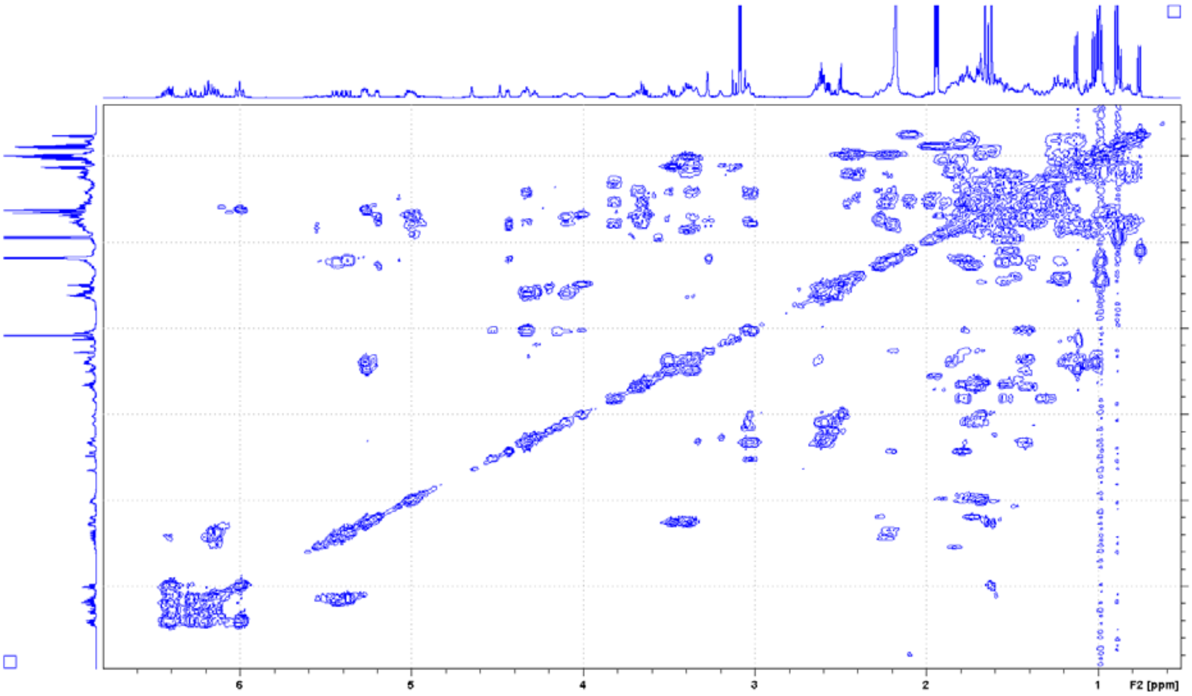

Supplementary Figure 42 | HSQC NMR spectrum ($CDCl_3$, 500 MHz) of compound 8.

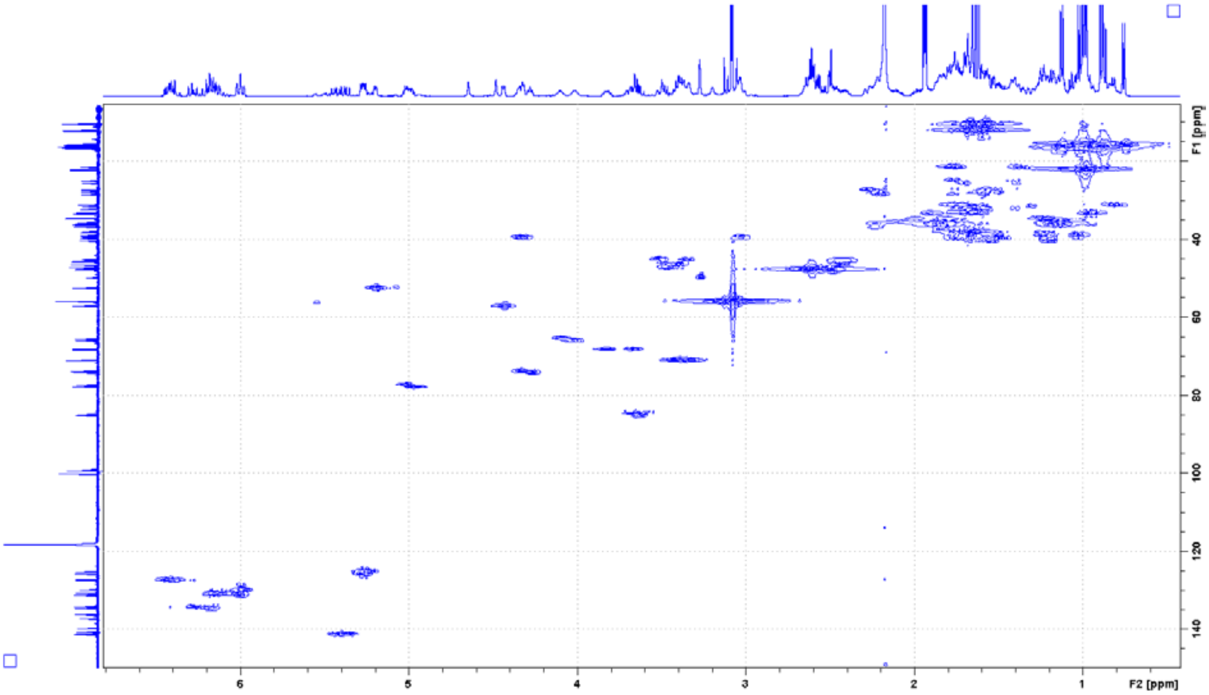

Supplementary Figure 43 | HMBC NMR spectrum ($CDCl_3$, 500 MHz) of compound 8.

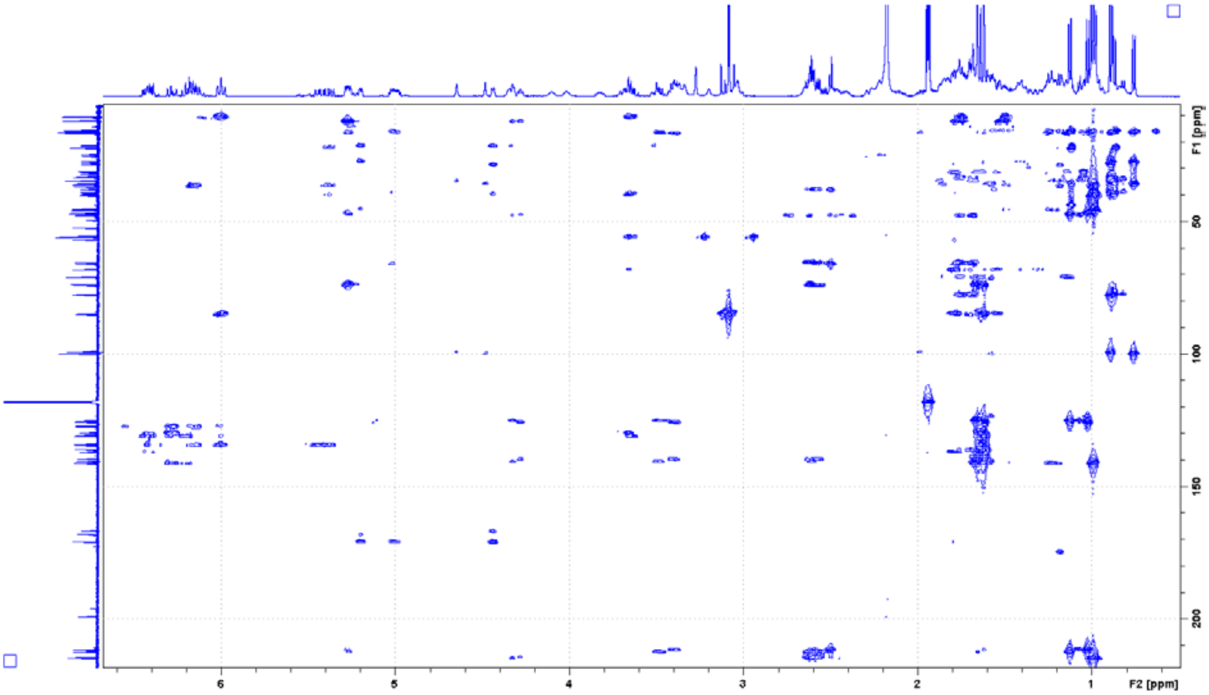

Supplementary Note 2 | Rapalog diversity generated through mutasynthesis. Five new rapalog producing strains were few exogenously with up to 32 different starter unit analogues, leading to a plethora of new structures as determined by HPLC-UV and LCMS/MS analysis,. No molecules were produced in the unfed controls and each new compound displayed the characteristic rapamycin UV chromophore. Markush structures are given based on the combination of: strain used, carboxylic acid fed, LCMS/MS fragmentation analysis and biosynthetic considerations.

Supplementary Figure 44 | Markush structure for rapalogs produced by feeding exogenous starter acids to strain ISOM-4141.

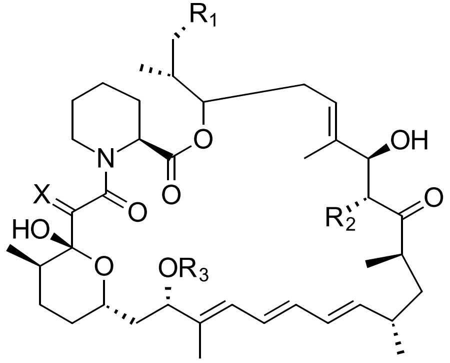

Supplementary Table 10 | LCMS data for rapalogs produced by feeding exogenous starter acids to strain ISOM-4141.

Strain	Feed	Feed structure	R_1	R_2	R_3	X	major product peaks RT (mins)	molecular weight
ISOM-4141	cyclohexanecarboxylic acid	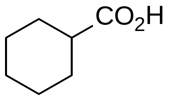	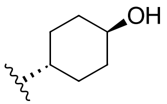	H	Me	O	10.1	797.5
ISOM-4141	cyclohex-1-enecarboxylic acid	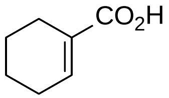	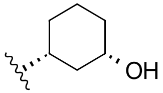	OH	Me	O	8.7	813.5
			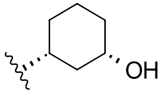	H	Me	O	9.6	797.5
			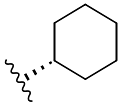	H	Me	O	10.6	781.5
ISOM-4141	cyclohex-3-enecarboxylic acid	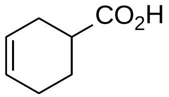	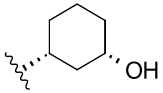	OH	Me	O	8.2	813.5
			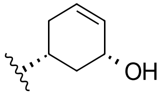	H	Me	O	9.8	795.5
			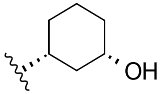	OH	Me	O	10.1	813.5

Strain	Feed	Feed structure	R_1	R_2	R_3	X	major product peaks RT (mins)	molecular weight
ISOM-4141	3-methoxycyclohexanecarboxylic acid	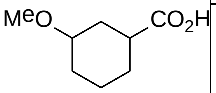	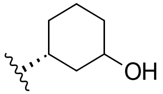	H	Me	O	10.6	797.5
			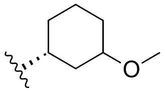	H	Me	O	10.8	813.5
			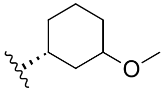	H	Me	H, H	11.3	797.5
ISOM-4141	Ethyl 5-hydroxycyclohex-3-enecarboxylic acid	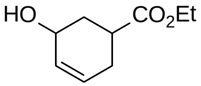	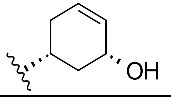	OH	Me	O	8.3	811.6
			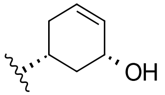	H	Me	O	10.2	795.5
ISOM-4141	(1S*,3S*,4R*)-4-fluoro-3-hydroxycyclohexanecarboxylic acid	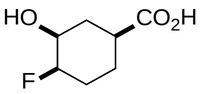	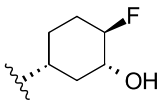	OH	Me	O	9.7	831.6
ISOM-4141	4-methylcyclohexanecarboxylic acid	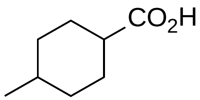	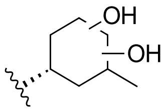	OH	Me	O	8.9	843.5
			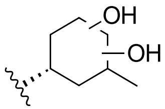	H	Me	O	9.5	827.5
			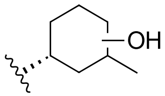	H	Me	O	10.1	811.5

Strain	Feed	Feed structure	R_1	R_2	R_3	X	major product peaks RT (mins)	molecular weight
ISOM-4141	cyclopentanecarboxylic acid	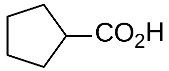	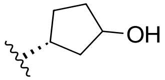	H	Me	O	10.1	783.5
ISOM-4141	cycloheptanecarboxylic acid	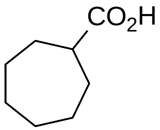	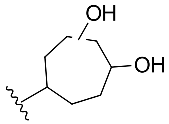	H	Me	O	10.1	827.2
			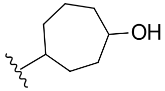	OH	Me	O	10.4	827.2
			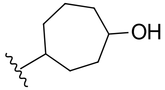	H	Me	O	10.9	811.6
ISOM-4141	(1R*,2S*,4S*)-bicyclo[2.2.1]heptane-2-carboxylic acid	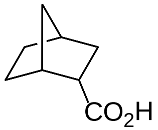	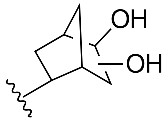	H	Me	O	9.3	825.6
			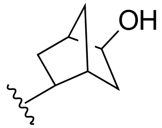	OH	Me	O	9.8	825.6
			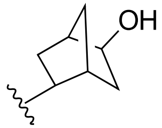	H	Me	O	10.5	809.5

Strain	Feed	Feed structure	R1	R2	R3	X	major product peaks RT (mins)	molecular weight
ISOM-4141	(1S*,2R*,5R*,6S*)-2-hydroxybicyclo[3.2.1] octane-6-carboxylic acid	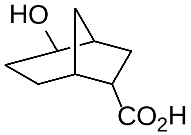	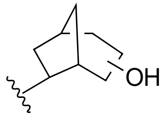	OH	H	O	8.9	839.5
			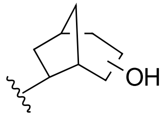	OH	Me	H, H	9.9	839.5
			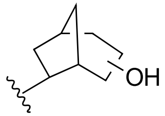	OMe	H	H, H	10.4	839.5
			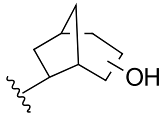	H	H	O	11.1	823.5
ISOM-4141	tetrahydro-2H-pyran-4-carboxylic acid	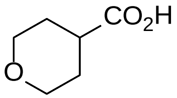	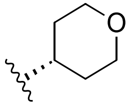	H	H	O	9.9	769.5
			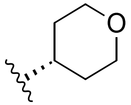	H	Me	O	11.7	783.4
ISOM-4141	tetrahydro-2H-thiopyran-4-carboxylic acid	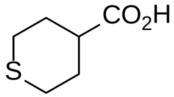	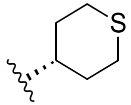	OH	Me	O	10.4	815.5
ISOM-4141	3-hydroxybenzoic acid	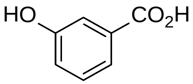	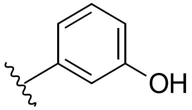	H	Me	O	10.4	791.5

Strain	Feed	Feed structure	R_1	R_2	R_3	X	major product peaks RT (mins)	molecular weight
ISOM-4141	4-methylthiophene-2-carboxylic acid	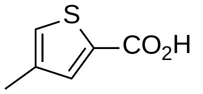	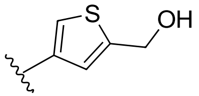	H	Me	O	9.9	811.5
ISOM-4141	4-hydroxy-3,3-dimethylcyclohexane carboxylic acid	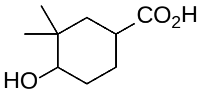	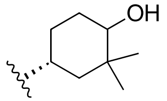	OH	Me	H, H	10.4	827.5
			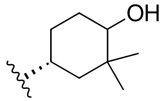	H	Me	H, H	10.9	811.5
			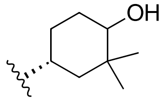	H	Me	O	11.6	825.5
ISOM-4141	4-methylenecyclohexanecarboxylic acid	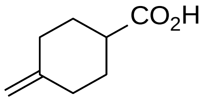	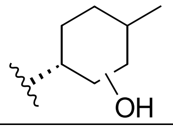	OH	Me	O	10.5	827.5
			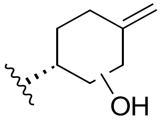	OH	Me	O	11.2	825.5
			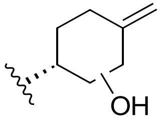	H	Me	O	11.7	809.5
ISOM-4141	4-methylcyclohex-3-enecarboxylic acid	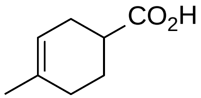	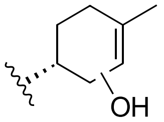	OH	Me	O	10.4	825.7
			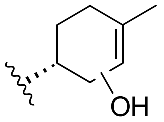	H	Me	O	10.7	809.5

Strain	Feed	Feed structure	R_1	R_2	R_3	X	major product peaks RT (mins)	molecular weight
ISOM-4141	(1S*,4S*)-4-methylcyclohexanecarboxylic acid	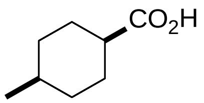	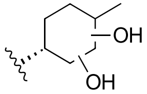	H	Me	O	8.9	827.6
			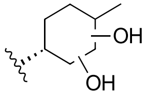	OH	Me	O	9.5	843.5
			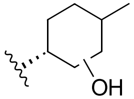	OH	Me	O	10.1	827.6
			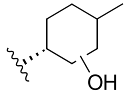	H	Me	O	10.7	811.5
ISOM-4141	(1R*,4R*)-4-hydroxycyclohexanecarboxylic acid	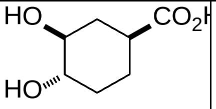	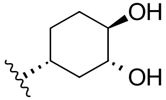	H	Me		10.1	797.9
ISOM-4141	methyl 3,3-difluoro-4-hydroxycyclohexanecarboxylate	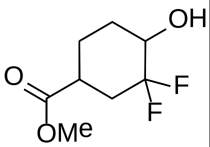	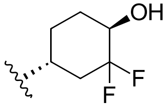	H	Me	O	9.9	833.9
			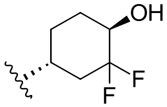	H	Me	H, H	10.9	819.9

Supplementary Figure 45 | Markush structure for rapalogs produced by feeding exogenous starter acids to strain ISOM-4144.

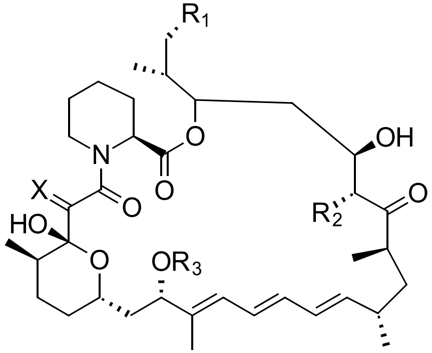

Supplementary Table 11 | LCMS data for rapalogs produced by feeding exogenous starter acids to strain ISOM-4144.

Strain	Feed	Feed structure	R_1	R_2	R_3	X	major product peaks RT (mins)	molecular weight
ISOM-4144	cyclohexanecarboxylic acid	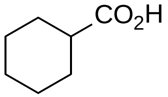	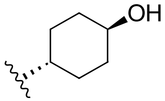	H	Me	O	9.6	757.5
ISOM-4144	cyclohex-1-enecarboxylic acid	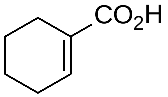	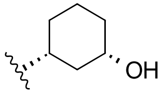	H	Me	H, H	7.8	743.6
			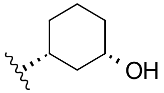	H	Me	O	9.6	757.5
ISOM-4144	cyclohex-3-enecarboxylic acid	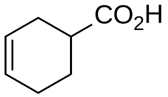	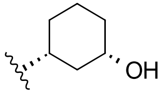	OH	Me	O	5.4	773.6
			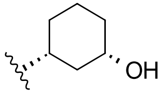	H	Me	O	7.5	757.6
ISOM-4144	3-methoxycyclohexanecarboxylic acid	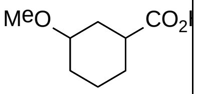	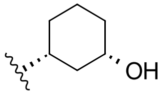	H	Me	H, H	7.9	743.5

Strain	Feed	Feed structure	R_1	R_2	R_3	X	major product peaks RT (mins)	molecular weight
ISOM-4144	Ethyl 5-hydroxycyclohex-3-enecarboxylic acid	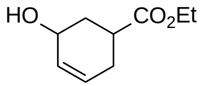	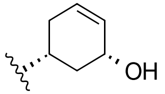	H	Me	H, H	7.5	741.6
			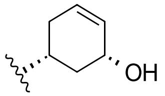	H	Me	O	9.5	755.6
ISOM-4144	(1S*,3S*,4R*)-4-fluoro-3-hydroxycyclohexanecarboxylic acid	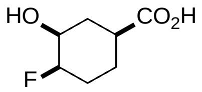	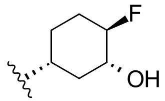	H	Me	O	9.2	775.6
ISOM-4144	4-methylcyclohexanecarboxylic acid	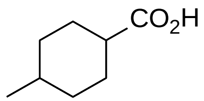	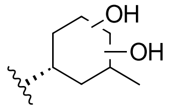	OH	Me	O	6.6	787.8
			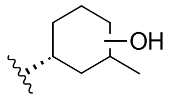	H	Me	H, H	8.9	757.8
			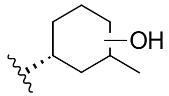	H	Me	O	10.2	771.6
			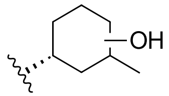	H	Me	O	11.3	771.6
ISOM-4144	cycloheptanecarboxylic acid	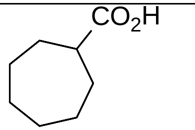	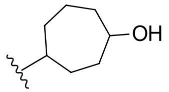	H	Me	O	10.6	771.7

Strain	Feed	Feed structure	R_1	R_2	R_3	X	major product peaks RT (mins)	molecular weight
ISOM-4144	(1R*,2S*,4S*)-bicyclo[2.2.1]heptane-2-carboxylic acid	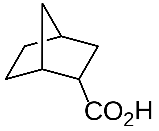	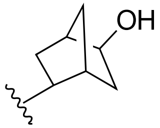	H	Me	O	7.8	769.6
			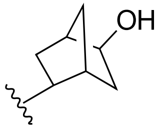	H	Me	H, H	9.9	755.8
ISOM-4144	(1S*,2R*,5R*,6S*)-2-hydroxybicyclo[3.2.1]octane-6-carboxylic acid	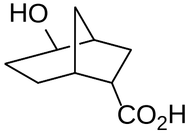	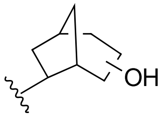	H	Me	H, H	8.5	769.8
			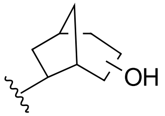	H	Me	O	9.1	783.5
			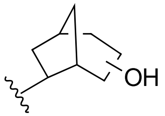	H	Me	O	10.6	783.5
ISOM-4144	4-methylthiophene-2-carboxylic acid	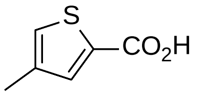	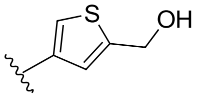	H	Me	O	9.3	771.6
ISOM-4144	4-hydroxy-3,3-dimethylcyclohexanecarboxylic acid	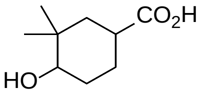	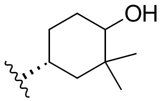	H	H	H, H	9.7	757.4
			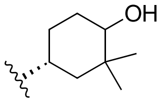	H	Me	H, H	10.5	771.7
ISOM-4144	4-methylenecyclohexanecarboxylic acid	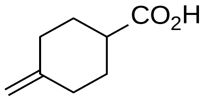	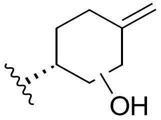	H	Me	O	11.6	769.6

Strain	Feed	structure	R_1	R_2	R_3	X	major product peaks RT (mins)	molecular weight
ISOM-4144	4-methylcyclohex-3-enecarboxylic acid	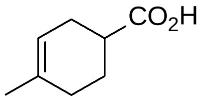	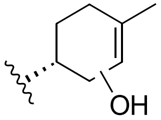	H	Me	O	10.6	769.6
ISOM-4144	(1S*,4S*)-4-methylcyclohexanecarboxylic acid	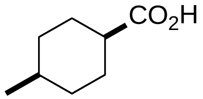	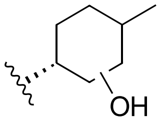	OH	Me	O	6.6	787.6
			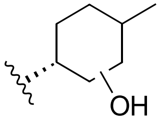	H	Me	O	10.3	771.7
ISOM-4144	(1S*,3S*,4S*)-3,4-dihydroxycyclohexanecarboxylic acid	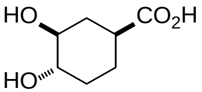	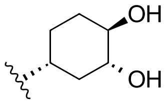	H	Me	H, H	7.2	759.7
			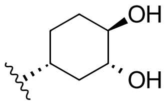	H	Me	O	7.5	773.7
ISOM-4144	3-methylcyclohexanecarboxylic acid	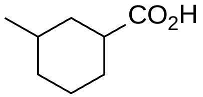	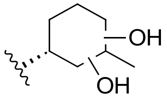	H	Me	O	6.7	787.6
			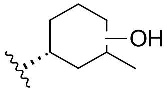	OH	Me	O	8.5	787.6
			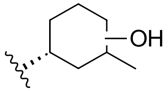	H	Me	O	10.6	771.7
ISOM-4144	5-methylthiophene-2-carboxylic acid	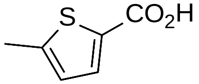	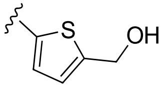	H	Me	O	9.3	771.6

Strain	Feed	Feed structure	R_1	R_2	R_3	X	major product peaks RT (mins)	molecular weight
ISOM-4144	(1*R**,4*R**)-4-hydroxycyclohexane carboxylic acid	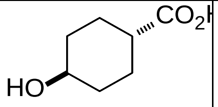	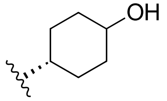	H	Me	O	7.3	757.6
ISOM-4144	(1S*,3S*)-3-hydroxycyclohexane carboxylic acid	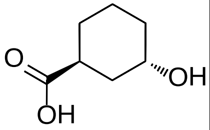	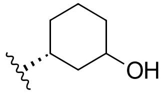	H	Me	O	10.9	757.9
			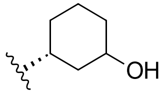	H	Me	O	11.2	757.9
ISOM-4144	methyl 3,3-difluoro-4-hydroxycyclohexane carboxylate	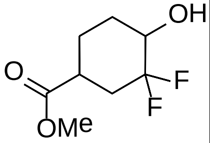	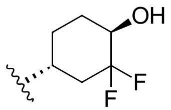	H	Me	O	9.2	793.9

Supplementary Figure 46 | Markush structure for rapalogs produced by feeding exogenous starter acids to strain ISOM-4185.

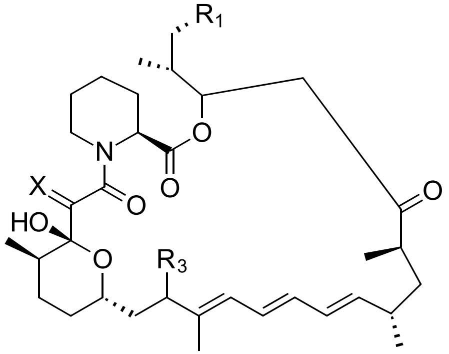

Supplementary Table 12 | LCMS data for rapalogs produced by feeding exogenous starter acids to strain ISOM-4185.

Strain	Feed	Feed structure	R_1	R_2	R_3	X	major product peaks RT (mins)	molecular weight
ISOM-4185	cyclohexanecarboxylic acid	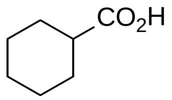	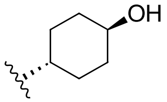		Me	H, H	8.4	699.5
			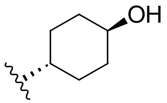		Me	O	10.8	713.6
ISOM-4185	cyclohex-1-enecarboxylic acid	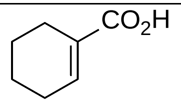	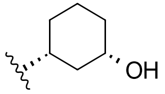		Me	H, H	8.8	699.6
ISOM-4185	cyclohex-3-enecarboxylic acid	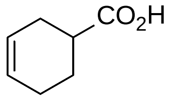	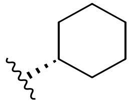		Me	O	8.3	697.6
ISOM-4185	3-methoxycyclohexanecarboxylic acid	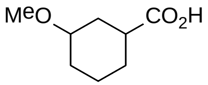	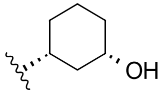		H	O	8.8	699.7
			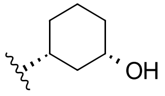		Me	H, H	9.4	699.7

Strain	Feed	Feed structure	R_1	R_2	R_3	X	major product peaks RT (mins)	molecular weight
ISOM-4185	Ethyl 5-hydroxycyclohex-3-enecarboxylic acid	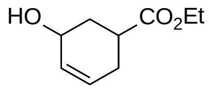	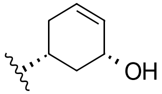		Me	H, H	8.3	697.6
ISOM-4185	(1S*,3S*,4R*)-4-fluoro-3-hydroxycyclohexanecarboxylic acid	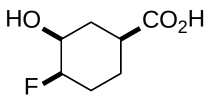	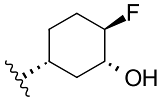		Me	H, H	6.9	717.6
			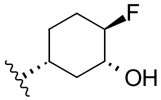		Me	O	7.8	731.6
ISOM-4185	4-methylcyclohexanecarboxylic acid	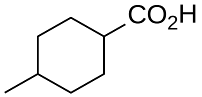	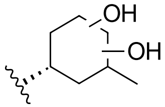		Me	H, H	7.5	729.6
			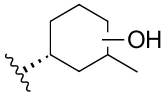		H	O	9.2	713.7
			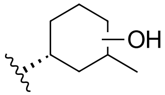		Me	H, H	10.1	713.7
ISOM-4185	cycloheptanecarboxylic acid	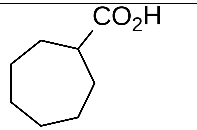	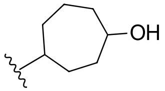		Me	H, H	8.6	713.6
ISOM-4185	(1R*,2S*,4S*)-bicyclo[2.2.1]heptane-2-carboxylic acid	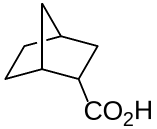	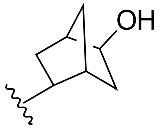		Me	H, H	8.9	711.6

**Strain**	**Feed**	**Feed structure**	**R_1**	**R_2**	**R_3**	**X**	**major product peaks RT (mins)**	**molecular weight**
ISOM-4185	(1S*,2R*,5R*,6S*)-2-hydroxybicyclo[3.2.1]octane-6-carboxylic acid	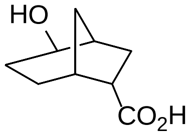	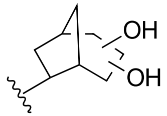		Me	H, H	7	741.6
			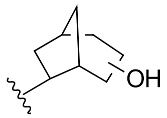		H	O	9.6	725.5
			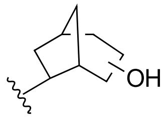		Me	H, H	10.2	725.5
ISOM-4185	3-hydroxybenzoic acid	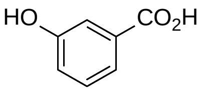	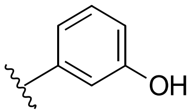		H	O	8.6	693.6
			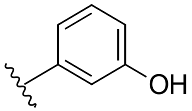		Me	H, H	11.8	693.6
ISOM-4185	4-hydroxy-3,3-dimethylcyclohexane carboxylic acid	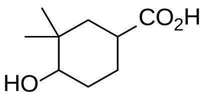	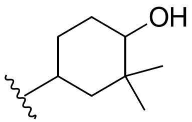		H	H, H	9.4	713.6
ISOM-4185	4-methylenecyclohexanecarboxylic acid	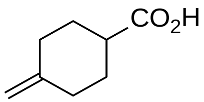	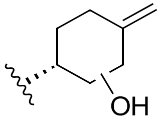		Me	H, H	9.8	711.7

Strain	Feed	Feed structure	R₁	R₂	R₃	X	major product peaks RT (mins)	molecular weight
ISOM-4185	4-methylcyclohex-3-enecarboxylic acid	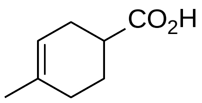	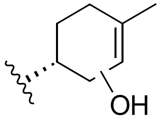		Me	H, H	9.5	711.5
			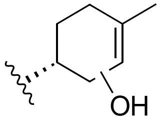		Me	O	12.2	725.5
ISOM-4185	(1S*,4S*)-4-methylcyclohexanecarboxylic acid	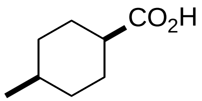	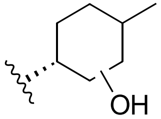		H	O	9.2	713.6
ISOM-4185	(1S*,3S*,4S*)-3,4-dihydroxycyclohexanecarboxylic acid	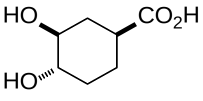	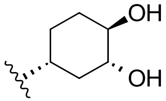		Me	O	7.1	729.7
ISOM-4185	3-methylcyclohexanecarboxylic acid	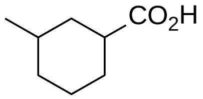	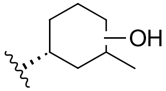		Me	H, H	9.5	713.7
			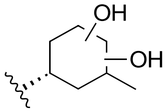		Me	O	9.9	743.6
ISOM-4185	5-methylthiophene-2-carboxylic acid	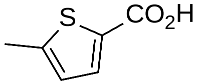	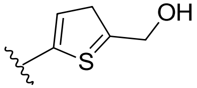		Me	H, H	8.1	713.5
ISOM-4185	(1R*,4R*)-4-hydroxycyclohexanecarboxylic acid	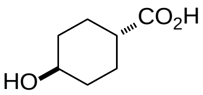	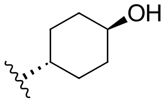		Me	H, H	8.4	699.8
			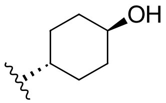		Me	O	10.8	713.7

Strain	Feed	Feed structure	R_1	R_2	R_3	X	major product peaks RT (mins)	molecular weight
ISOM-4185	(1S*,3S*)-3-hydroxycyclohexanecarboxylic acid	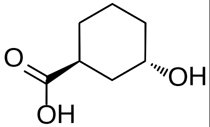	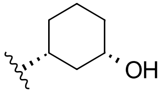		Me	H, H	9.4	699.9
ISOM-4185	(1S*,3R*,4S*)-methyl 3-fluoro-4-hydroxycyclohexanecarboxylate	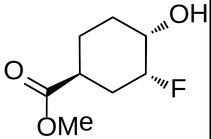	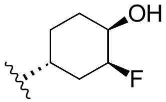		H	O	6.9	717.9
			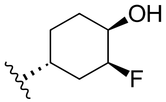		Me	H, H	7.9	717.9
ISOM-4185	(1S*,3R*)-3-hydroxycyclohexanecarboxylic acid	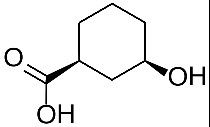	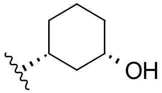		Me	H, H	8.8	699.9
			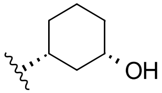		H	O	9.3	699.9
ISOM-4185	methyl 3,3-difluoro-4-hydroxycyclohexanecarboxylate	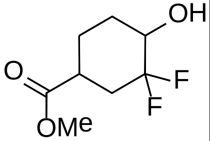	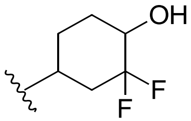		H	O	7.33	735.8
			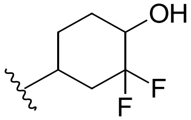		Me	O	8.2	749.9
			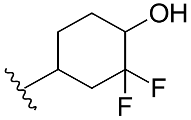		Me	H, H	10.3	735.8

Supplementary Figure 46 | Markush structure for rapalogs produced by feeding exogenous starter acids to strain ISOM-4185.

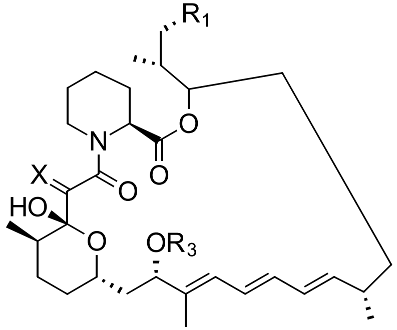

Supplementary Table 12 | LCMS data for rapalogs produced by feeding exogenous starter acids to strain ISOM-4185.

Strain	Feed	Feed structure	R_1	R_2	R_3	X	major product peaks RT (mins)	molecular weight
ISOM-4178	cyclohexanecarboxylic acid	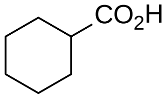	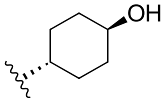		H	H, H	4.6	629.7
ISOM-4178	cyclohex-1-enecarboxylic acid	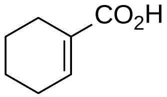	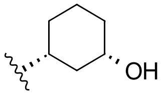		H	H, H	5.3	629.7
ISOM-4178	cyclohex-3-enecarboxylic acid	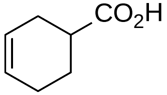	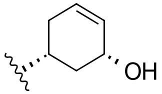		Me	O	8.4	655.8
ISOM-4178	(1S*,3S*,4R*)-4-fluoro-3-hydroxycyclohexanecarboxylic acid	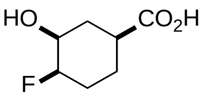	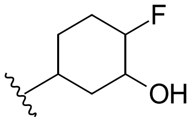		H	O	4.1	647.7
ISOM-4178	4-methylcyclohexanecarboxylic acid	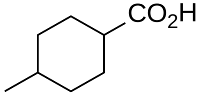	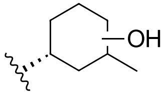		H	H, H	5.2	643.5
ISOM-4178	cycloheptanecarboxylic acid	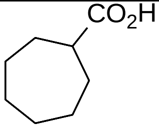	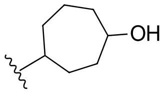		H	H, H	5.5	643.8

Strain	Feed	Feed structure	R_1	R_2	R_3	X	major product peaks RT (mins)	molecular weight
ISOM-4178	(1S*,2R*,5R*,6S*)-2-hydroxybicyclo[3.2.1]octane-6-carboxylic acid	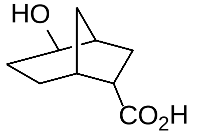	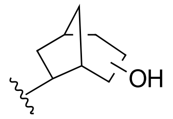		H	H, H	6.02	655.5
ISOM-4178	3-hydroxybenzoic acid	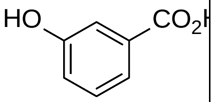	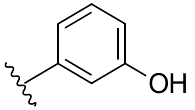		H	H, H	5.1	623.5
ISOM-4178	(1S*,3S*,4S*)-3,4-dihydroxycyclohexanecarboxylic acid	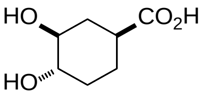	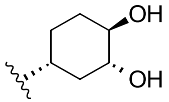		Me	O	8.4	673.6
ISOM-4178	(1S*,3R*,4S*)-methyl 3-fluoro-4-hydroxycyclohexanecarboxylate	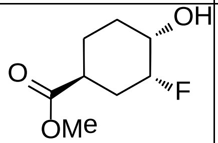	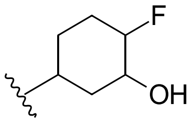		H	H, H	7.7	647.7

Supplementary Figure 47 | Markush structure for rapalogs produced by feeding exogenous starter acids to strain ISOM-4178.

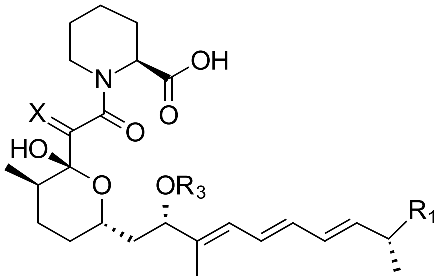

Supplementary Table 13 | LCMS data for rapalogs produced by feeding exogenous starter acids to strain ISOM-4178.

Strain	Feed	Feed structure	R_1	R_2	R_3	X	major product peaks RT (mins)	molecular weight
ISOM-4180	cyclohexanecarboxylic acid	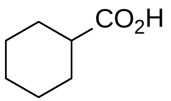	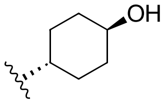		Me	H, H	5.1	561.5
			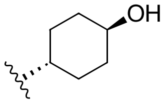		Me	O	6.7	575.5
ISOM-4180	cyclohex-1-enecarboxylic acid	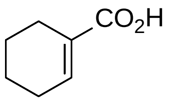	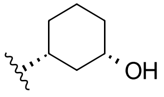		Me	H, H	5.07	561.5
			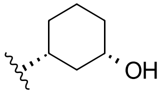		Me	O	6.7	575.5
ISOM-4180	cyclohex-3-enecarboxylic acid	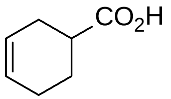	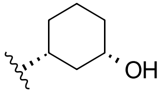		Me	H, H	5.1	561.4
			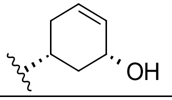		Me	O	6.1	573.7
ISOM-4180	(1S*,3S*,4S*)-3,4-dihydroxycyclohexane carboxylic acid	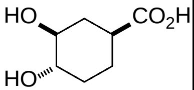	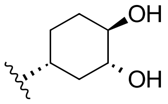		Me	O	3.9	591.9

Strain	Feed	Feed structure	R_1	R_2	R_3	X	major product peaks RT (mins)	molecular weight
ISOM-4180	3-methoxycyclohexanecarboxylic acid	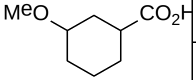	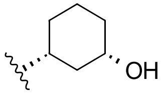		Me	H, H	5	561.4
			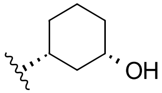		Me	O	6.7	575.5
ISOM-4180	cycloheptanecarboxylic acid	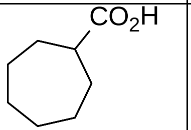	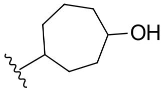		Me	O	7.8	590
ISOM-4180	(1S*,2R*,5R*,6S*)-2-hydroxybicyclo[3.2.1]octane-6-carboxylic acid	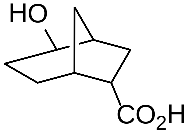	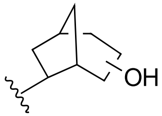		Me	O	7.8	601.8
ISOM-4180	3-hydroxybenzoic acid	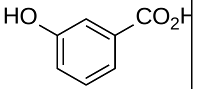	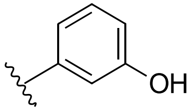		Me	O	6.4	569.9
ISOM-4180	4-hydroxy-3,3-dimethylcyclohexanecarboxylic acid	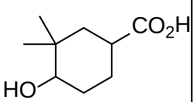	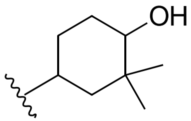		Me	O	8.9	603.8
ISOM-4180	(1S*,4S*)-4-methylcyclohexanecarboxylic acid	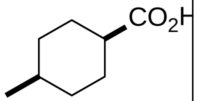	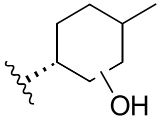		Me	O	7.6	589.9
ISOM-4180	(1S*,3S*)-3-hydroxycyclohexanecarboxylic acid	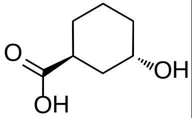	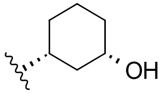		Me	O	7.8	575.6

Strain	Feed	Feed structure	R₁	R₂	R₃	X	major product peaks RT (mins)	molecular weight
ISOM-4180	3-methylcyclohexanecarboxylic acid	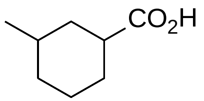	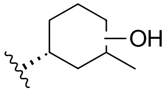		Me	H, H	6.3	575.6
			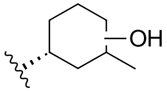		Me	O	8.02	589.9
ISOM-4180	(1R*,4R*)-4-hydroxycyclohexanecarboxylic acid	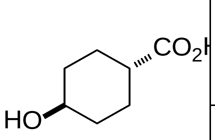	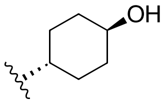		Me	H, H	5.1	561.7
			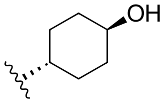		Me	O	6.7	575.8
ISOM-4180	(1S*,3R*)-3-hydroxycyclohexanecarboxylic acid	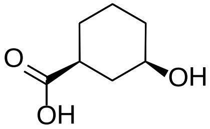	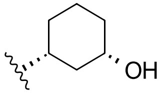		Me	O	6.7	575.8
ISOM-4180	(1S*,3R*,4S*)-methyl 3-fluoro-4-hydroxycyclohexanecarboxylate	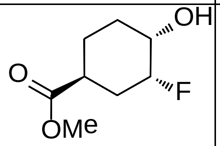	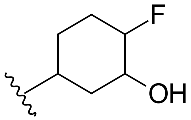		Me	O	5.9	593.7
ISOM-4180	(1S*,3R*,4S*)-3-ethyl-4-hydroxycyclohexanecarboxylic acid	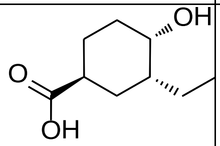	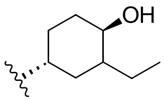		Me	O	8.6	603.7
ISOM-4180	methyl 3,3-difluoro-4-hydroxycyclohexanecarboxylate	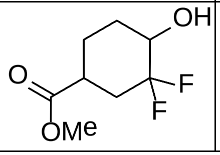	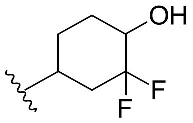		Me	O	6.1	611.8

Supplementary References

1. Kendrew, S. G. *et al*. Recombinant strains for the enhanced production of bioengineered rapalogs. *Metab. Eng*. **15**, 167-173 (2013).
